# GorillaFACS: The Facial Action Coding System for the *Gorilla spp*.

**DOI:** 10.1371/journal.pone.0308790

**Published:** 2025-01-28

**Authors:** Catia Correia-Caeiro, Raquel Costa, Misato Hayashi, Anne Burrows, Jordan Pater, Takako Miyabe-Nishiwaki, Jack L. Richardson, Martha M. Robbins, Bridget Waller, Katja Liebal

**Affiliations:** 1 Human Biology & Primate Cognition Department, Institute of Biology, Leipzig University, Leipzig, Germany; 2 Comparative Cultural Psychology, Max Planck Institute for Evolutionary Anthropology, Leipzig, Germany; 3 Research Department, Japan Monkey Center, Inuyama, Japan; 4 Primate Cognition Research Group, Lisbon, Portugal; 5 Mulheres pela Primatologia, Florianópolis, Brazil; 6 Department of Physical Therapy, Duquesne University, Pittsburgh, PA, United States of America; 7 Department of Anthropology, University of Pittsburgh, Pittsburgh, PA, United States of America; 8 Center for the Evolutionary Origins of Human Behavior (EHuB), Kyoto University, Inuyama, Japan; 9 Center for the Advanced Study of Human Paleobiology, Department of Anthropology, The George Washington University, Washington, DC, United States of America; 10 Department of Primate Behavior and Evolution, Max Planck Institute for Evolutionary Anthropology, Leipzig, Germany; 11 Department of Psychology, Nottingham Trent University, Nottingham, United Kingdom; Ohio State University, UNITED STATES OF AMERICA

## Abstract

The Facial Action Coding System (FACS) is an objective observation tool for measuring human facial behaviour. It avoids subjective attributions of meaning by objectively measuring independent movements linked to facial muscles, called Action Units (AUs). FACS has been adapted to 11 other taxa, including most apes, macaques and domestic animals, but not yet gorillas. To carry out cross species studies of facial expressions within and beyond apes, gorillas need to be included in such studies. Hence, we developed the GorillaFACS for the *Gorilla spp*. We followed similar methodology as previous FACS: First, we examined the facial muscular plan of the gorilla. Second, we analysed gorilla videos in a wide variety of contexts to identify their spontaneous facial movements. Third, we classified the individual facial movements according to appearance changes produced by the corresponding underlying musculature. A diverse repertoire of 42 facial movements was identified in the gorilla, including 28 AUs and 14 Action Descriptors, with several new movements not identified in the HumanFACS. Although some of the movements in gorillas differ from humans, the total number of AUs is comparable to the HumanFACS (32 AUs). Importantly, the gorilla’s range of facial movements was larger than expected, suggesting a more relevant role in social interactions than what was previously assumed. GorillaFACS is a scientific tool to measure facial movements, and thus, will allow us to better understand the gorilla’s expressions and communication. Furthermore, GorillaFACS has the potential be used as an important tool to evaluate this species welfare, particularly in settings of close proximity to humans.

## Introduction

### The Facial Action Coding Systems for humans and animals

The Facial Action Coding System (FACS) has been a widely used tool in human facial expression research since Ekman and colleagues made it available in a training manual [1–3]. FACS is a standardised coding system, which identifies and describes in detail the brief facial movements of the human face based on the corresponding musculature. Whenever a facial muscle contracts, parts of the skin move, leading to noticeable changes in the face’s appearance. These movements are caused by mimetic muscles and are referred to as Action Units (AU). AUs are assigned a distinct numerical code and a descriptive name. For instance, "AU12" corresponds to "Action Unit 12 –Lip Corner Puller" and is coded when the zygomaticus major muscle pulls the lip corners towards the ears. In order to code broad movements or non-mimetic muscle actions, the human FACS manual also includes Action Descriptors (ADs), for example, tongue movements, since these can impact the appearance changes of AUs [2].

By using the Facial Action Coding System (FACS), it becomes possible to systematically and objectively identify facial movements based solely on the visible changes that occur when a muscle underneath the skin contracts. FACS categorises these movements into subunits (i.e., AUs), rather than analysing whole facial expressions, which typically consist of multiple AUs [4]. This tool eliminates the subjective interpretation that can arise from observers’ perceptions, thereby reducing emotional or contextual biases. FACS is a reliable system that considers individual differences in facial structure, such as bone configuration, fat deposits, and permanent wrinkles, by utilizing common facial landmarks and setting minimum criteria for coding an AU. A neutral face, where no facial muscles are contracted, is typically required for accurate FACS coding to differentiate between individual variations and avoid incorrect coding of false appearance changes.

Following the same methodology as used for the human system, FACS has been modified for use with several other primate species: chimpanzees (ChimpFACS [5]), rhesus [6], Barbary [7], Japanese [8], and crested macaques [9] (MaqFACS), hylobatids (GibbonFACS [10]), orangutans (OrangFACS [11]), and common marmosets (CalliFACS [12]) and three domesticated species: dogs (DogFACS [13]), horses (EquiFACS [14]), and cats (CatFACS [15]). The adaptation of FACS for other species is based on the examination of anatomical homologies (e.g., [16–18]) while accounting for species differences in facial morphology. The development of AnimalFACS for different species not only allows new insights into the objective and standardised study of animal communication within each species, but also creates a framework for inter-specific comparative and evolutionary perspectives on facial communication and emotional processes [19, 20].

By applying these AnimalFACS tools, novel insights have been found about the communication systems of animals. For example, AnimalFACS have helped understand the manner in which dogs and cats communicate with humans [13, 15, 21] and how these species react facially in emotional contexts [22–25]. In NHP, AnimalFACS have also revealed the complexities of communication and emotion in a variety of species, including the fact that orangutan [26] and gibbon [27] play faces meet the behavioural criteria for intentionality, that the same facial expression in crested macaques (Silent-Bared Teeth) has different meanings depending on which AUs are included in the facial expression [9], that hylobatids pair-bonding is related to facial expressions [28], or that species previously thought to be less facially expressive, such as common marmosets, have a similar potential for facial movements as other NHP [12]. More recently, a cross-comparison study of macaque species combined AnimalFACS and network analysis to look into the social complexity hypothesis, revealing that social tolerance is associated with higher complexity in facial expressions [29].

These studies demonstrate the highly complex and dynamic nature of facial expressions and highlight the need for FACS tools for the functional understanding of facial expressions. Likewise, for gorillas, GorillaFACS has the potential to identify and measure variation in their known facial expressions and/or reveal the full potentiality for facial movement in gorillas, whilst better framing possible functions of each facial expression. Importantly, because FACS relies solely on anatomy and does not use *a priori* assumptions about meaning, it avoids anthropomorphic judgements that may presume underlying (human) emotions. Hence, FACS are important tools that can be directly applied in the investigation of complex facial displays in humans and other mammals, to not only precisely measure its subunits (i.e., AUs) and tease apart their meaning (e.g., whether certain movements are linked to communication and/or emotion), but also to carry out comparative studies among species, that may be more (e.g., chimpanzees) or less (e.g., dogs) phylogenetically related to humans and ascertain how evolutionary processes may have driven facial behaviour (e.g., where do human’s highly complex facial expressions come from or which species share which facial displays with humans).

### Why adapt FACS for gorillas?

Currently, gorillas are classified into two species: the western gorillas (*Gorilla gorilla*) and the eastern gorillas (*Gorilla beringei*), which are divided into four subspecies, including the Cross River Gorilla (*Gorilla gorilla diehli*), Western Lowland Gorilla (*Gorilla gorilla gorilla*), Mountain Gorilla (*Gorilla beringei beringei*), and Eastern Lowland Gorilla (or Grauer’s gorillas, *Gorilla beringei graueri*). Their habitat ranges from the equatorial rainforests to mountain forests of Central and East Africa, including Uganda, Rwanda, Democratic Republic of Congo, Republic of Congo, Angola, Central African Republic, Equatorial Guinea, Cameroon, Gabon, and Nigeria. Gorillas are primarily herbivorous, consuming a diet of leaves, fruits, shoots, and stems. *G*. *gorilla* and *G*. *b*. *graueri* are more frugivorous, while *G*. *b*. *beringei* lean towards folivory [30, 31]. While gorillas are not territorial, social units are very cohesive, and their movements are influenced by factors such as food availability and the likelihood of interactions with other groups of gorillas or solitary males [32–34]. Group size varies between 2–65 individuals per group, with the Virunga population of *G*. *b*. *beringei* having the larger average groups (12.5 ± 9.1), in comparison to the Bwindi population of *G*. *b*. *beringei* (9.6 ± 6.4), *G*. *b*. *graueri* (10.0 ± 6.3), and *G*. *g*. *gorilla* (8.4 ± 4.3) [31, 35–39].

With some slight variation between different subspecies, gorillas practice a polygynous (lowland) or polygynandrous (mountain) mating system [40, 41]. Both males and females disperse from the natal group, with females transferring to different groups depending on their reproductive status among other factors [40, 42, 43]. To navigate diverse social contexts during integration in new groups, individuals are required to mediate conflicts and maintain group cohesion [44]. Similarly, in captivity, gorillas were shown to have a remarkable capacity to adjust social interactions in function of husbandry conditions and artificial composition of their group [45]. Hence, despite being considered to have weaker social bonds than chimpanzees [46], and low facially expressivity among primates [47, 48] due to their phylogenetic position [49], gorillas often use different communicative signals in intra and inter-group interactions [50–53]. For instance, they often use agonistic signals like prolonged gazing, chest beating, and other visual displays, aimed at managing negative or tense social interactions [54]. Facial expressions, gestures, and body movements are part of these important visual displays, often indicating which interactions are aggressive, sexual, or affiliative [55].

The ability to perceive and mimic facial expressions between gorillas has been demonstrated to synchronise and promote play interactions, highlighting the significance of facial communication in fostering social bonds and understanding emotional cues within their intricate social dynamics [56]. In addition to intra-specific communication, gorillas also need to rely on such signals during interactions with humans, especially in the context of close proximity to tourists [57] or zoo visitors [58, 59]. In fact, gorillas use facial expressions in response to the attentional state of a human experimenter, meeting the criteria of intentional communication with humans [60]. However, and despite some obvious facial morphological differences between gorillas and humans (e.g., salient browridge, lack of chin), at least some facial movements are similar, which could lead humans to anthropomorphically misinterpret gorillas’ facial expressions (e.g., mistaking a "fear grimace" as a "friendly smile"). Conversely, gorillas could also misinterpret human facial expressions, potentially identifying these as signals of aggression (although not yet shown in gorillas, these inter-specific perceptual misunderstandings were shown in Barbary macaques [61, 62] and dogs [63]). Therefore, the (mis)communication between these two species has significant implications for both animal welfare and human safety.

Despite the increasing risk for close proximity interactions with humans in their wild habitat [57], facial expressions in gorillas have seldom been studied in social contexts [51], and not at all in interactions with humans. Furthermore, the few studies of gorilla facial expressions either describe facial expressions in a holistic way (i.e., one label for the full facial display that might include several facial movements) or are identified by broadly descriptive terms (e.g., "stare") or emotionally-loaded terms (e.g., "frown"), which make objective comparisons between studies difficult. While this initial work on gorilla facial expressions has laid the groundwork for us to understand their diverse and complex behavioural repertoire, to date, a more detailed examination of their facial expressions has yet to be undertaken. Hence, this calls for the development of a more objective tool to accurately record and analyse gorillas’ facial expressions, eliminating anthropomorphic and emotional biases [12].

In this study, we introduce the GorillaFACS (Gorilla Facial Action Coding System), the first objective, systematic, and quantifiable tool for the scientific measurement of gorilla facial movements. It is based on the underlying musculature of the gorilla face and its muscular homologies to the human face, following the methodology of the human FACS, which was initially developed to study human facial behaviour [1–3].

Hence, the aims of this work are: 1) Define an anatomical and functional plan of gorilla facial muscles using published dissections and homologies with the human facial musculature; 2) Identify the corresponding facial movements of gorillas through spontaneous facial movements analysis and categorisation into AUs, 3) Develop the GorillaFACS manual (i.e., the current article) to instruct users in becoming certified to identify AUs in gorillas, and 4) to serve as a reference for coding AUs in future research with gorillas.

## Methodology

This work was approved by the Ethics Advisory Board of the Leipzig University (Ref. 2023.04.06_eb_191). All work undertaken for this manuscript was purely observational.

Following a similar methodology used in previous FACS adaptations [12], we employed a three-step methodology to develop the GorillaFACS: the first step consisted of examining the facial muscular plan of the gorilla; in the second step we analysed videos of spontaneous behaviour of gorillas to identify facial movements; finally, in the third step, we combined the anatomical information with the observed facial movements to classify each of these facial movements into AUs and ADs. The following sections provide a detailed description of this three-step methodology.

### Determination of the facial muscular plan

**Fig 1:**
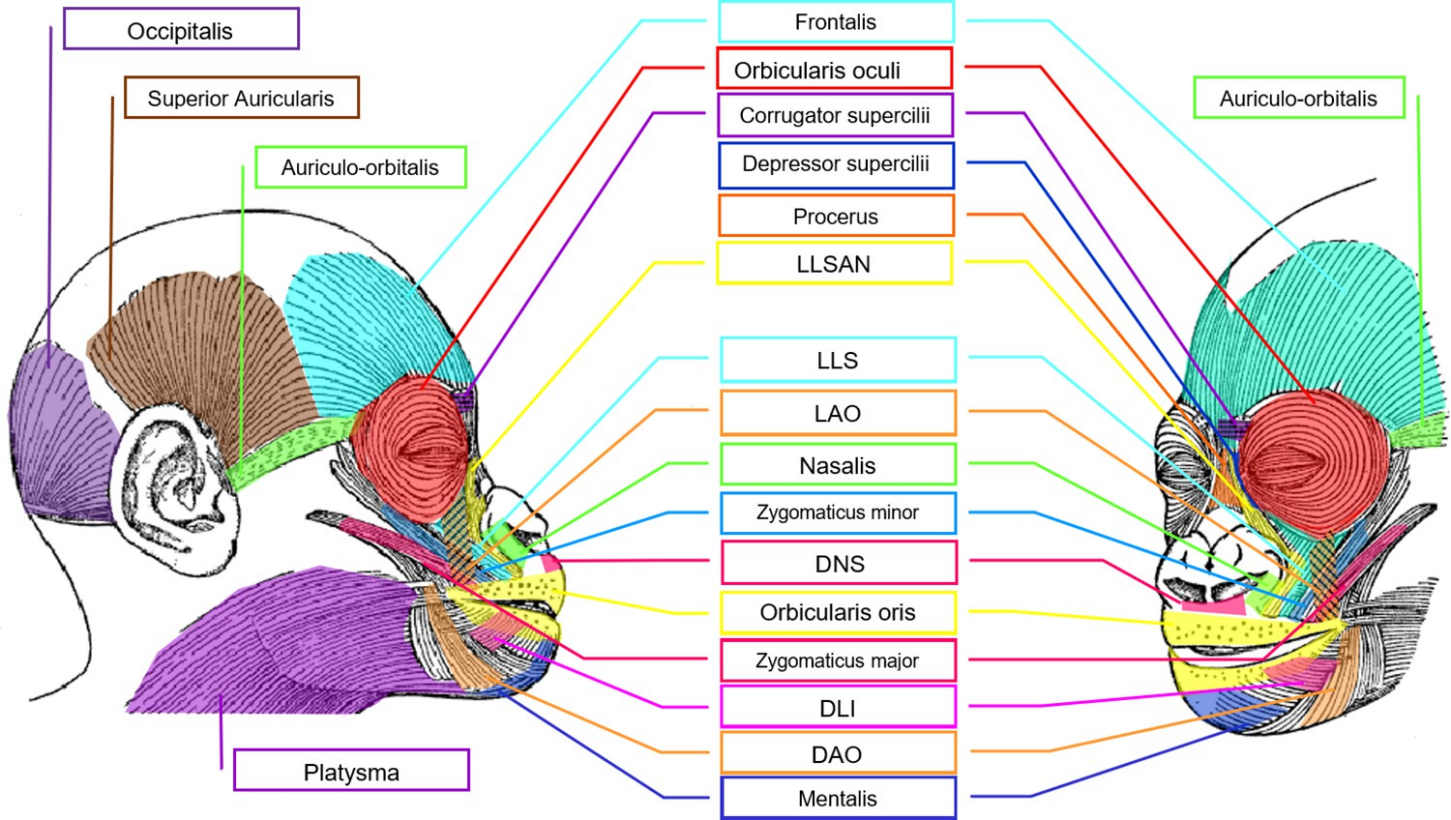
Schematic representation of the facial musculature in the gorilla in lateral and 45 degree angle views (image adapted from Huber [71] and based on published dissections [64, 65]). Labels: DAO: Depressor anguli oris, DLI: Depressor labii inferioris, DNS: Depressor nasi septi, LAO: Levator anguli oris, LLS: Levator labii superioris, LLSAN: Levator labii superioris alaeque nasi.

The first step in adapting this system for gorillas was to establish the facial muscular plan by consulting published literature containing gorilla facial muscle dissections [64, 65], summarised in **Table 1** and illustrated in **Fig 1**. These were also compared with human facial muscles [2].

**Table 1:** Description of facial muscles found in gorillas in the published dissections of the gorilla face [64, 65]. We also indicate if the muscle is commonly found in humans [2]. ✓- present, x–absent, NA–not dissected.

	Gorillas		Humans
Muscle	Diogo et al 2010	Rotenstreich & Marom 2023	
Occipitalis	✓	NA	✓
Frontalis	✓	✓	✓
Platysma	✓	✓	✓
Orbicularis Oculi	✓	✓	✓
Levator Palpebrae Superioris	X	X	✓^1^
Corrugator Supercilii	✓	X	✓
Procerus	✓	✓	✓
Depressor Supercilii	✓	✓	✓
Zygomaticus Minor	✓	✓	✓
Zygomaticus Major	✓	✓	✓
Nasalis	✓	✓	✓
Depressor Nasi Septi	✓	✓	✓
Levator Labii Superioris Alaeque Nasi	✓	✓^2^	✓
Levator Labii Superioris	✓	✓^2^	✓
Levator Anguli Oris/Caninus	✓	✓^3^	✓
Orbicularis Oris	✓	✓	✓
Depressor Anguli Oris	✓	✓^3^	✓
Depressor Labii Inferioris	✓	✓	✓
Mentalis	✓	✓	✓
Risorius	✓	X^4^	✓

^1^Muscle present in humans [2] and macaques [66], and suggested to be present in all primates [67], but not reported in any of the gorilla dissections.

^2^Rotenstreich & Marom (2023) describe this within the “quadratus labii superioris” muscle.

^3^Rotenstreich & Marom (2023) describe this within the “caninus-triangularis complex”.

^4^Rotenstreich & Marom (2023) are unsure if it is present.

**Fig 2:**
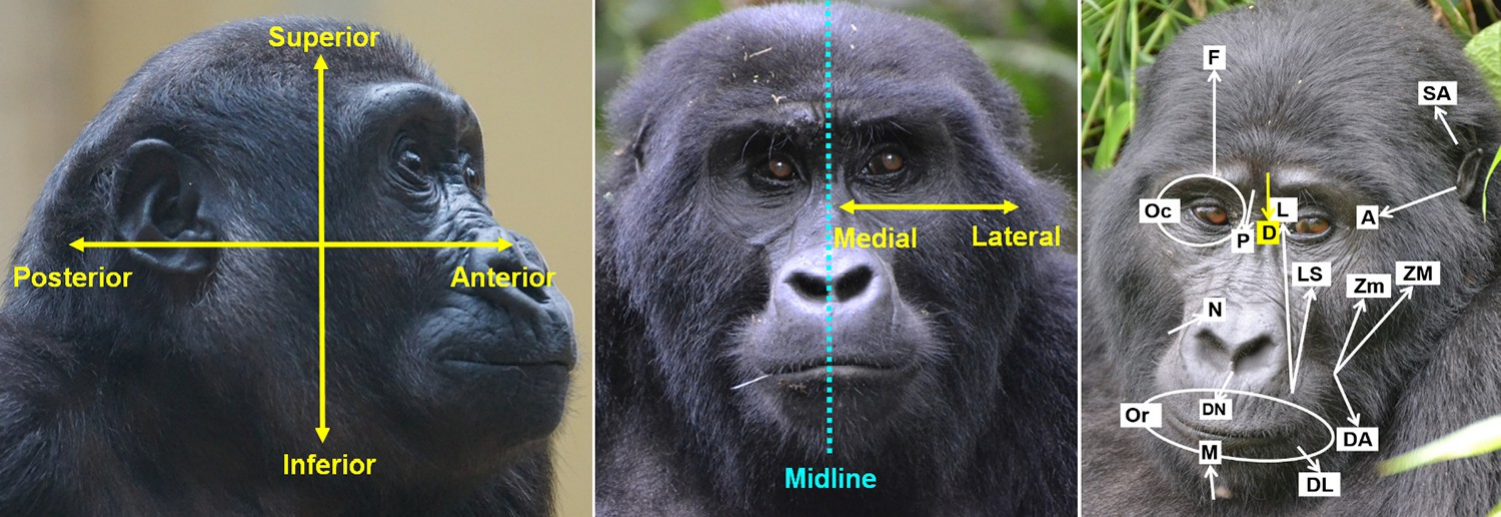
Left and Centre: Spatial representation of positional and directional terminology for the gorilla head/face. We adopted here the terminology used for the standard anatomical position in humans (individual in bipedal position, facing forward). This follows the FACS of other apes [5, 11]. Superior-Inferior: feature or structure or direction of movement that is above or below another one, respectively, in the vertical axis of the head (longitudinal axis). Anterior-Posterior: feature or structure or direction of movement that is in front or behind another one, respectively, along the long axis of the head (sagittal axis). Medial: towards the medial plane (represented by the midline) of the head (transversal axis). Lateral: from the medial plane, towards the left or right side of the head (transversal axis). **Right: Direction of muscle contraction.** Labels represent approximate points of muscle origin, arrow end represents approximate point of muscle insertion, and arrow head represents the direction of muscle contraction (towards the label, except for the Or and Oc muscles, which have no clear insertion and contract concentrically). Labels: A—Auriculo-orbitalis, D—Depressor supercilii, DA—Depressor anguli oris, DL—Depressor labii inferioris, DN–Depressor nasi septi, F—Frontalis, L—Levator labii superioris alaeque nasi, LS—Levator labii superioris, M—Mentalis, N—Nasalis, Oc—Orbicularis oculi, Or—Orbicularis oris, P—Procerus, SA—Superioris auricularis, ZM—Zygomaticus major, Zm—Zygomaticus minor. The occipitalis (O) is not represented here as they are not visible from a frontal view. The O inserts in the galea aponeurotica, originates in the occipital region, and contracts anteroposteriorly. Left picture by Pixel-mixer user from Pixabay.com, centre and right picture by RC.

We compared the anatomical information from the gorilla with the known human facial musculature in order to identify possible functional homologies [68]. The proposed muscle function in gorillas is illustrated in **Fig 2**, based on points of origin, insertion and fibre direction. For the development of the FACS for humans, chimpanzees [69] and rhesus macaques [70], intramuscular electrical stimulation was performed to confirm and validate facial muscle function. Since validation of facial muscle function has been done in these species and due to ethical concerns of performing this procedure in gorillas [11, 69], the muscle function here described for the gorilla was based on published dissections [64, 65] and functional homologies [68] between humans and other primates.

### Identification of facial movements

In the second step of developing the GorillaFACS video recordings of spontaneous facial movements of gorillas were analysed with the aim of 1) identifying the facial movements (AUs and ADs) gorillas display, 2) finding at least one clear example of each facial movement, and 3) extracting short videos to illustrate these examples (included in this manuscript as **Supporting Information Videos–S1 Videos zip target**). A sample of approximately 21 hours and 24 minutes of video was analysed (by CCC) frame-by-frame. The videos were selected according to FACS visibility criteria of the head and face (e.g., lighting, proximity, video quality), and included variable frame rate (30–60 FPS). This sample featured over 200 individuals (it was not always possible to verify the identity of the individuals in the footage) in a variety of populations and including three of the four gorilla subspecies: Western Lowland gorillas (*G*. *g*. *gorilla*) housed in zoos—full list in **SI1 in S1 File** (10h28m of video), Eastern Lowland gorillas (*G*. *b*. *graueri*) housed at the GRACE sanctuary (2h33m of video), and Mountain gorillas (*G*. *b*. *beringei*) from wild populations comprising (8h22m of video). The sample also included a variety of contexts, including potentially positive, negative, and neutral contexts: e.g., resting, grooming, feeding, play, aggression, copulation, human interaction.

The videos were reused from other ethically approved research projects unrelated to the present work, or sourced from online public databases (e.g., YouTube.com, Gracegorillas.org, all with a Creative Commons Licence or approval from the video owners). Therefore, no negative contexts (e.g., pain, distress) were induced during the current work and/or solely for the purpose of developing GorillaFACS. Still images were extracted from videos in some instances or downloaded from public databases (e.g., Pixabay.com, Unsplash.com) to illustrate particular facial features or aid in the identification of appearance changes. We recognise that it is possible that movements displayed in very specific contexts (e.g., birthing) or that are rare may be missing from our video sample. However, if additional movements are found in the future, they can be added to the GorillaFACS through the AnimalFACS platform (www.animalFACS.com).

### Classification of facial movements into Action Units and Action Descriptors

In the final step of developing GorillaFACS, the anatomical plan and behavioural video analysis were integrated. This combination allowed for the detailed description of the facial movements observed in the videos, using precise directional and anatomical terms (**Fig 2**, and **SI2 in S1 File** for a glossary). These were classified according to codes used in previous FACS (including AUs and ADs), following functional muscular homologies wherever possible, or creating new codes whenever the homologies were not identified (for instance, by adding "1" before the code used for the original FACS).

### Facial morphology in gorillas

Since facial morphology is unique to each species, identifying facial movements relies heavily on specific facial landmarks and other anatomical reference points. Hence, it is important to become familiar with gorilla facial morphology, which is described below, with particular remarks on facial features that differ between the subspecies, sexes, and potential individual differences.

The genus *Gorilla* is the most sexually dimorphic of the African apes in terms of size [72], with adult males significantly larger than females, presenting outward movement of the nasal region, and increased prognathism due to larger canines, when compared to females [73].

**Fig 3:**
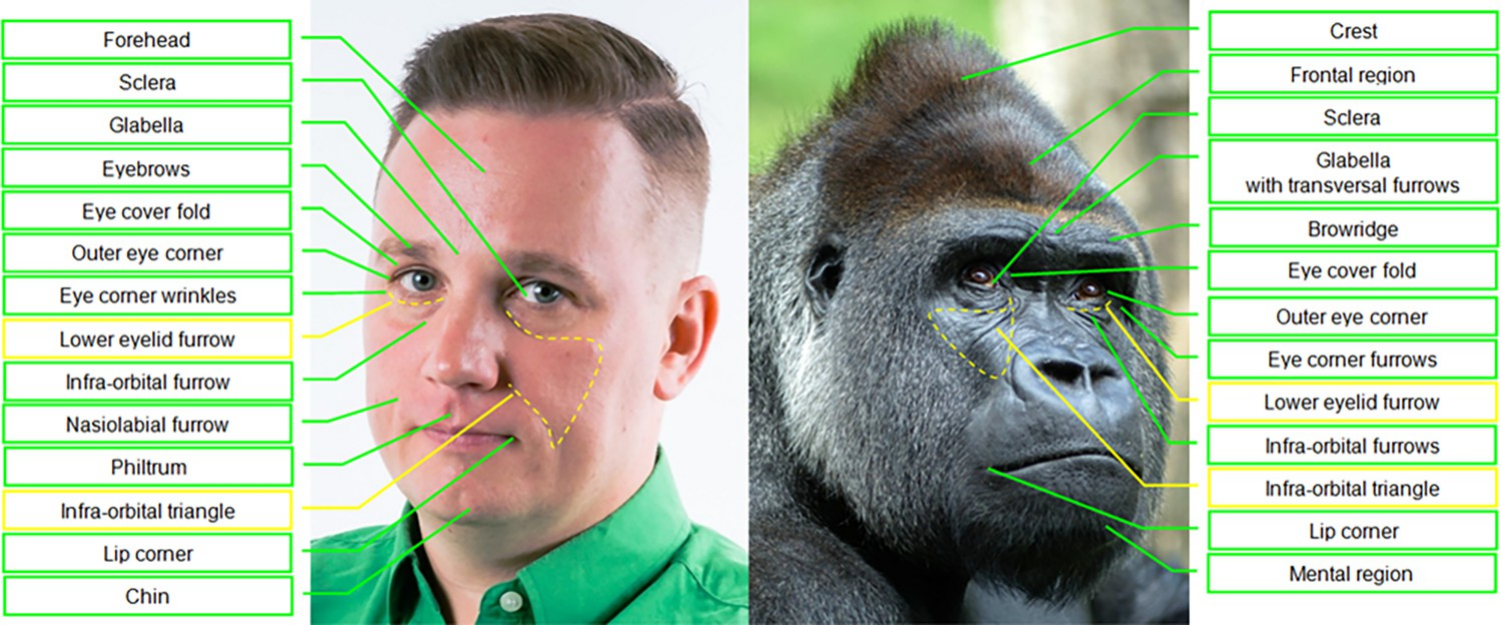
Comparison of facial landmarks of humans and gorillas. Pictures by tarasnesterenko1 (left) and willems_87 (right) from Pixabay.com.

**Fig 4:**
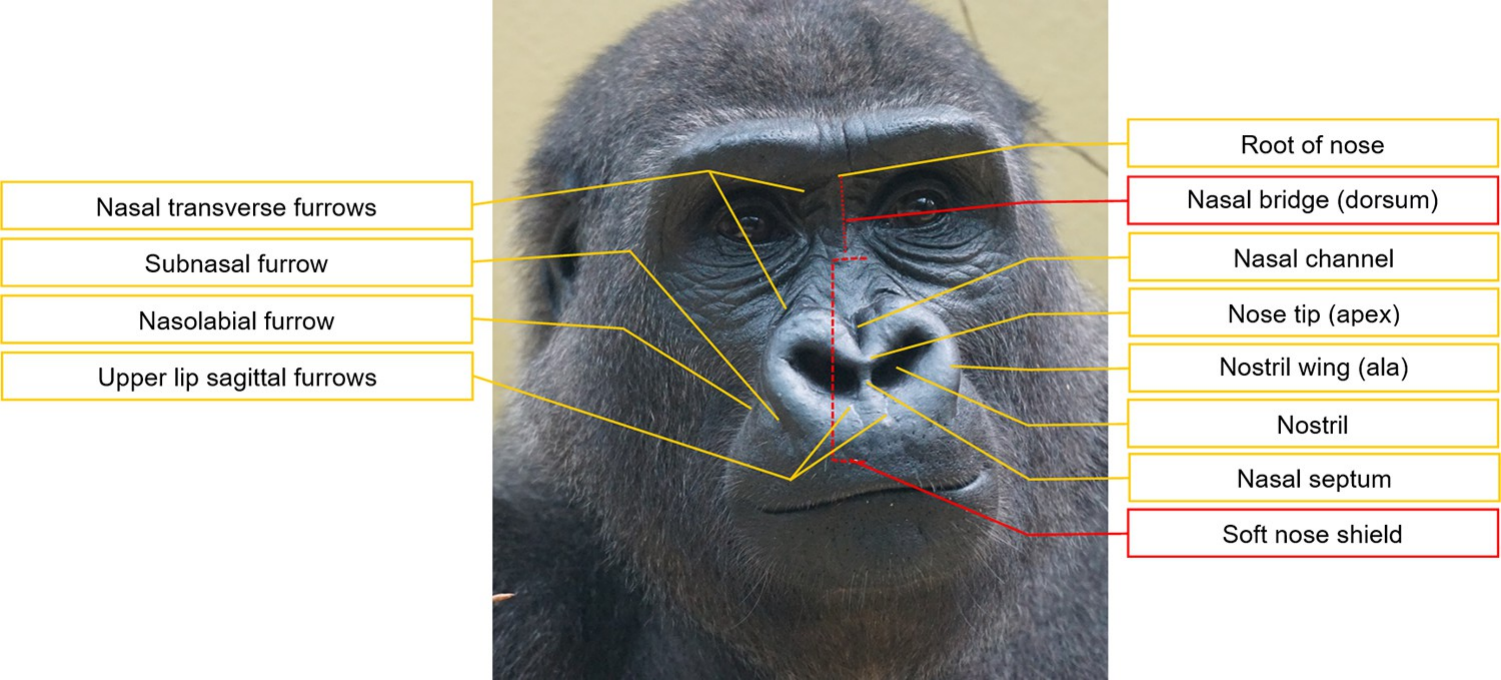
Nasal region landmarks of gorillas. Picture by Pixel-mixer user from Pixabay.com.

Regarding their facial landmarks (**Figs 3** and **4**), gorillas present a very prominent browridge, similar to other apes. The nose shield of gorillas is larger and more prominent in relation to other parts of the face than in the other apes, due to the highly developed wings bulging laterally and even dorsally. However, similarly to humans, there is no clear separation between the nose and the upper lip in gorillas, because the subnasal furrow is lacking in this genus. In chimpanzees, this furrow is marked and important to identify mouth and nose region AUs, and in orangutans is also present, although less marked [74]. In gorillas, due to the basal area of the soft nose shield and nostril wings merging into the upper lip, independent movements of the nose or upper lip may be harder to distinguish.

Gorillas present a unique specialisation of the subcutaneous connective tissue above the frontal region called a crest (or crown-pad), observed in both sexes, but generally larger in males. The crests are similar tissues to the cheek pads of the orangutan, but the gorilla crest has low vascularisation and not much fat, while the orangutan cheek pads have high fat content, more vascular and with some muscle bundles insertions [75]. Crests are thought to be a product of female mate choice [76], and its presence drastically changes the facial appearance of individuals.

**Fig 5:**
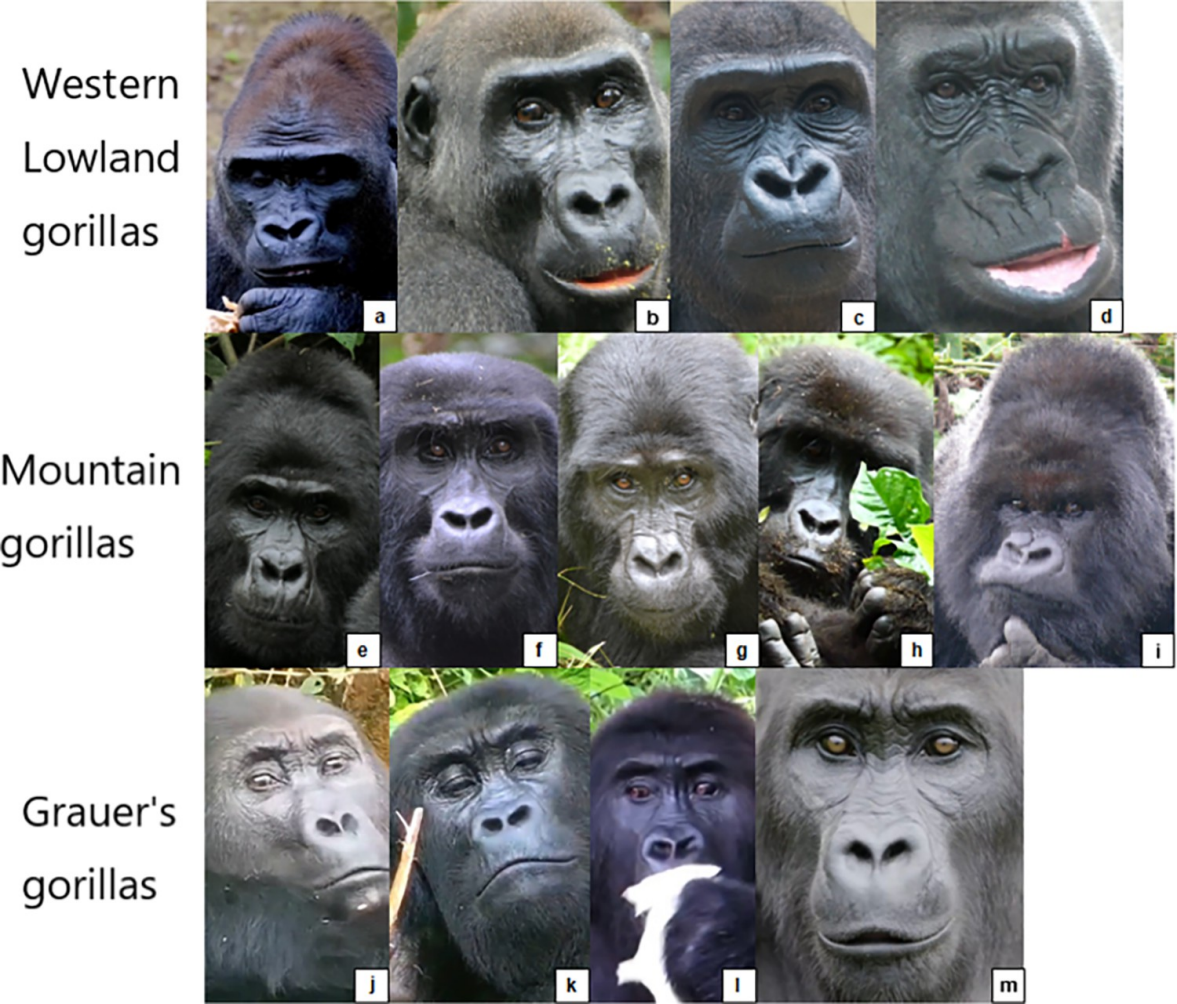
Examples of variation in hair coverage in frontal region and browridge between subspecies and individuals. a) Individual without hair on frontal region, exposing skin and permanent wrinkles. b)—d) Individuals with short dense hair on frontal region, but little or no hair on browridge. e)—i) Variation in hair coverage, from almost no hair covering the browridge in image e) to browridge fully covered in hair in images h) and i). j)–m) individuals with short hair right above the browridge and without hair on the browridge. Western lowland gorillas (*G*. *g*. *gorilla*) (a-d) seem to present a more prominent continuous browridge curving laterally, whilst mountain gorillas (*G*. *b*. *beringei*) and Grauer’s gorillas (*G*. *b*. *graueri*) tend to present less prominent browridges with two more or less salient brow arches above each eye and a slight depression on the glabella. Cross River gorillas (*G*. *g*. *diehli*) are not depicted here as due to their low population numbers and lack of studies, their typical facial features are not well known. Pictures credits: still frame from video NickyPe (a), MM (b and i) Pixel-mixer (c), Bart Brebels (d), RC (e-h), and still frames from gracegorillas.org videos (j-m).

Usually the frontal region is covered in hair, but some individuals may have a naked frontal region, exposing the frontal skin, which displays wrinkles in a neutral state—this is a false appearance change for AU1+2, i.e., the presence of wrinkles does not indicate movement per se in the case of gorillas (e.g., **Fig 5**).

However, these features present some variation depending on the subspecies. To the best of our knowledge, there has not yet been published a detailed study comparing facial features differences among subspecies, and any noted differences are highly descriptive or scattered through the literature. For example, according to Coolidge [77], *G*. *b*. *beringei* have a longer palate, narrower skull, thicker and more black hair, more developed crests, whilst Schultz [78] described *G*. *b*. *beringei* as having a higher face and eyes closer together. Other differences have been noted between the Eastern and Western species [79]: the form of the nose is angular with narrow nostrils in *G*. *beringei*, while in *G*. *gorilla* the nose is rounded and padded at the sides with flared nostrils. A “lip” above the septum is present in *G*. *gorilla*, while it is weak or absent in *G*. *beringei*. In general, the nose extends further down the upper lip in *G*. *gorilla*. Within the *G*. *beringei*, *G*. *b*. *beringei* have longer hair on the head, including the browridge and beard, when compared to the *G*. *b*. *graueri*. *G*. *b*. *graueri* and *G*. *gorilla* may have brown hair on the head while the hair of *G*. *b*. *beringei* tends to be black. In *G*. *b*. *graueri*, the hairs are shorter on the scalp and around the face. The upper lip of *G*. *b*. *graueri* is described by some authors [79] as more salient and appearing convex in a lateral view, whilst in *G*. *b*. *beringei* the lip is less salient.

However, these facial differences do not seem to be only at the subspecies level, but also extend to a population level. For example, within *G*. *b*. *beringei* [79], Bwindi gorillas have short facial hair with visible ears, without a beard, sparser hair on the browridge, while Virunga gorillas have long facial hair that hides the ears and forms a beard or whiskers on the face, and hairier browridges. Gorilla’s nasal shields shapes and sizes display an enormous variation between individuals, creating the characteristic “nose print”. Nose prints seem to be most prominent for Virunga than Bwindi populations of *G*. *b*. *beringei*, and even less obvious *G*. *gorilla*. This nose print may or may not include wrinkles, furrows, and/or lines on the soft nose shield, nasal channel, and above the nostrils. In Bwindi gorillas, the nose has no nasal channel, but Virunga gorillas show a strong nasal channel, and the nostrils are relatively large. In addition, Bwindi gorillas have longer faces, lower and narrower ascending rami, and shorter mandibles than the Virunga gorillas. Within *G*. *gorilla*, comparisons are harder, as there is not a lot of information on *G*. *g*. *diehli*, so most of the information is on the abundant *G*. *g*. *gorilla* subspecies. The latter exhibits some differences within the large subspecies and at a population level, for example on skull shape: the skulls of the Cameroon Plateau gorillas tend to be large with a broad skull; those from the coast of Cameroon and Gabon are smaller, with narrower skulls; those from the swampy Sangha River region are large like the Cameroon Plateau population but with shorter faces and smaller jaws [79].

**Fig 6:**
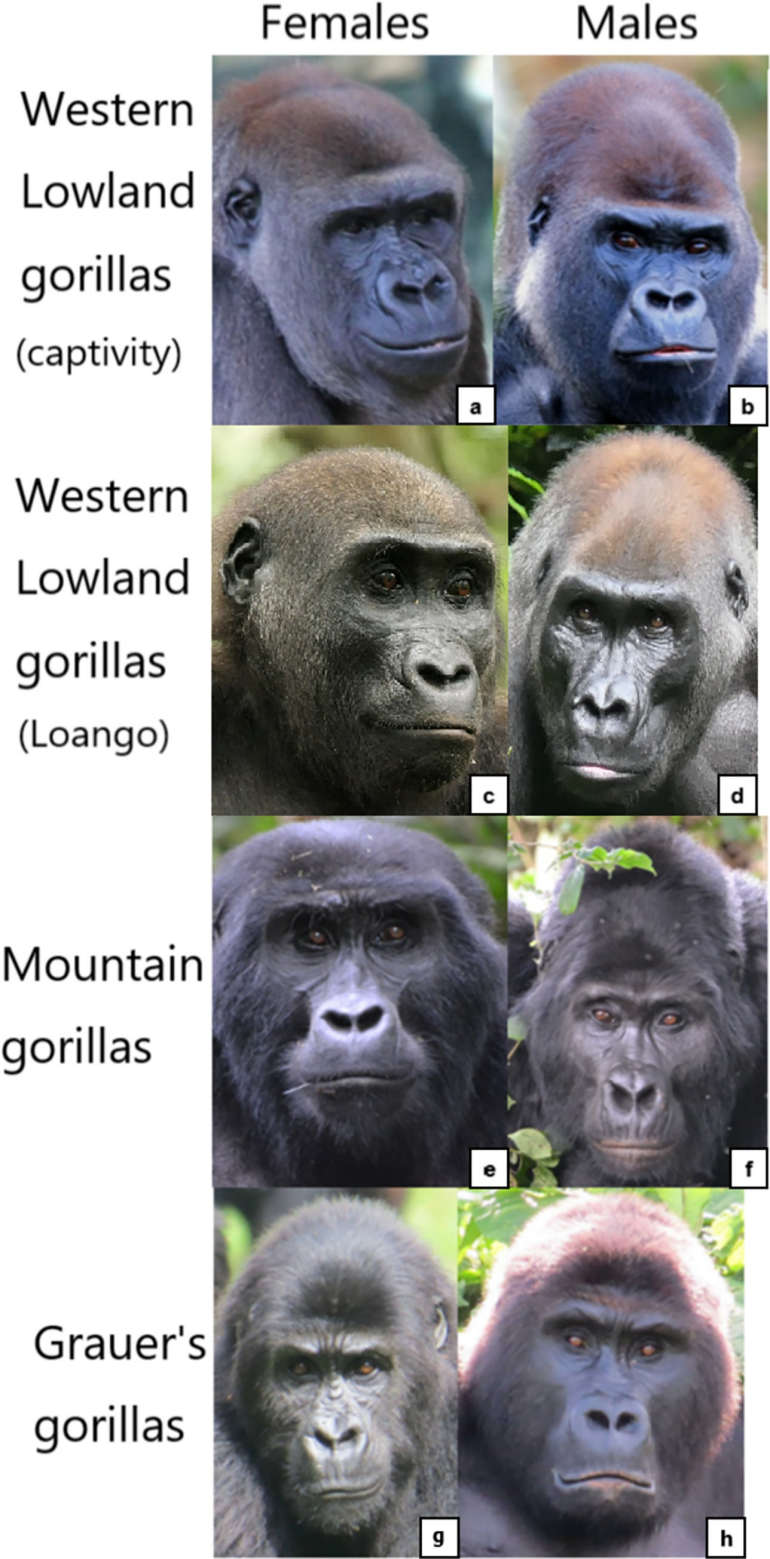
Examples of variation of facial morphology between sex, subspecies, and populations of gorillas. Pictures credits: a) by veverkolog user from Pixabay.com, b) still from video by NickyPe from Pixabay.com, c) and d) by MM, e) and f) by RC, g) and h) from gorillasgrace.org.

Although none of this variation in facial feature have yet been studied systematically between populations of subspecies, personal observations from the authors of this manuscript confirm some of the observations above. For example, there seems to be a defining difference in hair coverage of face between subspecies (e.g., **Fig 5**). In *G*. *b*. *beringei*, browridges are sometimes covered in hair, whilst in lowland gorillas browridges are more visible, which may modify appearance changes for AU1+2 and AU4. *G*. *b*. *graueri* are distinguishable by their longer faces, whilst *G*. *g*. *diehli* have larger and more salient lips. Similar to *G*. *b*. *beringei*, *G*. *b*. *graueri* have less salient browridges than *G*. *gorilla* (e.g., **Fig 6**).

### How to use GorillaFACS as a coding tool

In **Fig 1**, a diagram illustrates the muscular structure of the gorilla’s face, showing the locations of each muscle. The direction of action for each muscle is indicated. The muscle abbreviation labels mark the approximate origin of the muscle on the bone structure. The opposite end of the arrow points to the approximate insertion point where the muscle attaches to the skin. When a muscle contracts, it pulls the skin toward the label position, usually causing the skin to bunch or wrinkle perpendicularly to the muscle’s pull direction. For example, in AU1+2, the frontalis muscle originates from the frontal bone and attaches to the superior fibres of the orbicularis oculi and depressor supercilii muscles (**Table 1**, [49]). These diagrams demonstrate the underlying musculature and its function, aiding in the understanding of facial appearance changes produced during AUs. For specific anatomical terms and definitions, refer to **SI2 –Glossary in S1 File**.

This manuscript includes all the AUs and ADs found in gorillas, along with a list of appearance changes describing in detail the visual results of each muscular movement on the face of the gorilla. Each movement is illustrated by still images and/or short video-clips (see **SI Videos**). Each AU also sets minimum criteria, in which the presence of specific visible appearance change(s) are a condition required to code an AU. This is to avoid coding very low intensity muscular activity, which may be hard to detect by the human eye and thus cannot be coded by FACS.

As with all FACS systems, anyone interested can become a certified GorillaFACS coder. Due to the objective nature of FACS, no prior experience with FACS or the target species is necessary [14]. To become certified in GorillaFACS, individuals must self-study the GorillaFACS Manual (this article and its **SI Videos**) before taking a certification test, which includes 25 videos described in the next section "Coding Reliability," to ensure system reliability. GorillaFACS learners should thoroughly study the anatomical information (refer to previous sections on anatomy and morphology), appearance changes, and the minimum criteria described for each AU/AD (see results section) and illustrated in the **SI Videos**, before attempting the certification test. The GorillaFACS certification test is available for free upon request at www.animalFACS.com.

### Coding reliability

We tested inter-observer reliability between two FACS coders (CCC: certified in HumanFACS [2] and in all the AnimalFACS developed to date [5, 6, 8, 10–15]; RC: certified in ChimpFACS [5]) by coding 25 short videos (not used to describe the AUs). Inter-observer reliability was used to: (1) confirm both coders could reliably identify AUs included on the GorillaFACS manual, and (2) to refine the descriptions of AUs through discussion when agreement between coders on a particular AU was low. This was followed by additional rounds of coding using the same 25 videos to confirm that inter-observer reliability had sufficiently improved. In each coding round, one of the coders (RC) was always blind to the other coder (CCC) scores.

The coders’ overall reliability (Wexler’s index [80], equation (1)) and the AUs independent coding agreement (calculated through the average of each AU agreement) from a first round of coding (**Table 2**) indicated an agreement between coders of 70%, which is considered an acceptable agreement. However, individual AU agreement was in some cases low, hence a second round of coding was performed with the same videos. This second round increased the overall reliability to 81% (**Table 2**), which is considered a "very good" agreement [3, 81], but some AUs were still low. Hence, in a third and final round of coding focused on particular problematic AUs, we obtained a slightly higher mean agreement of 83% from the Wexler’s index [80] (1), and also a good or very good independent coding agreement on most AUs (**Table 2**). The final “coding key” from these reliability videos will act as a certification test for future GorillaFACS coders, in which an agreement score of 70% or more will be required to ensure the robustness of the GorillaFACS (as per the Wexler’s index and [68]).


$${\left(1\right)\;Wexler}^{'}s\;index\;=\;\frac{\left(Number\;of\;AUs\;on\;which\;coder\;1\;and\;Coder\;2\;agreed\right)\times 2}{The\;total\;number\;of\;AUs\;scored\;by\;the\;two\;coders}$$

**Table 2:** Mean Wexler’s index [80] (1) and independent coding agreement for each AU and AD in the three coding rounds. NA denotes instances where all coders agreed that a particular Action was not present in any of the clips.

AUs / ADs	Round 1	Round 2	Round 3
Wexler’s index (overall agreement)	0.70	0.81	0.83
AU1+2: Brow raiser	0.86	0.75	0.71
AU4: Brow Lowerer	0.57	0.77	0.77
AU6: Cheek Raiser	1.00	0.86	0.86
AU7: Lid Tightener	0.00^1^	1.00	0.80
AU43: Eye Closure	0.00^1^	0.50^1^	0.50^1^
AU45: Blink	0.92	1.00	0.91
AU47: Half-blink	1.00	1.00	1.00
AU9: Nose Wrinkler	0.85	0.85	0.88
AU10: Upper Lip Raiser	0.67	0.67	0.73
AU12: Lip Corner Puller	0.43	0.63	0.62
AU14: Dimpler	0.00^1^	0.80	1.00
AU16: Lower Lip Depressor	0.20	0.75	0.80
AU160: Lower Lip Relax	0.50	1.00	1.00
AU17: Chin Raiser	0.33	0.55	0.77
AU18: Lip Pucker	0.59	0.77	0.77
AU22: Lip Funneler	0.75	1.00	1.00
AU122: Lower Lip Inner Curl	NA	NA	NA
AU222: Lower Lip Extension	0.80	1.00	0.80
AU24: Lip Presser	0.40	0.91	0.91
AU25: Lips Part	0.96	1.00	1.00
AU26: Jaw Drop	0.67	0.86	0.83
AU27: Mouth Stretch	0.86	0.93	0.93
AU28: Lips Suck	0.80	0.00^1^	1.00
AU38: Nostril Dilator	1.00	0.57	0.80
AU39: Nostril Compressor	NA	NA	NA
AU138: Nose Shield Expander	0.00^1^	0.75	1.00
AU139: Nose Shield Flattener	1.00	0.00^1^	0.00^1^
AU238: Nose Downwards	NA	NA	NA
AD19: Tongue Show	0.00^1^	0.00^1^	0.00^1^
AD190: Tongue Downwards	NA	NA	NA
AD191: Tongue Curl	NA	NA	NA

^1^Low agreement due to rarely coded AUs/ADs (≤3 occurrences), not due to low agreement between coders.

## Results

The results presented here serve not only as a report of the facial movements identified during the development of GorillaFACS for gorillas but also as a manual for future GorillaFACS coders. This manual helps coders learn to identify these facial movements and serves as a guide for GorillaFACS coding post-certification. All the AUs identified in gorillas are listed in **Table 3**, along with the corresponding muscles. Additionally, **Table 4** describes the ADs found in gorillas (see **SI3** for ADs description and **SI4-SI6** for other useful ADs codes in S1 File). Each movement is described in detail and illustrated with pictures and videos in the **S1 Video in S1 zip target**.

**Table 3:** Comparison between FACS Action Units (AU) for humans [2] and gorillas according to underlying musculature [64, 65]. ✓- present, x—absent. Cells highlighted in grey are AUs present in gorillas.

AU code	AU name	Underlying muscle	Human	Gorilla
**AU1**	**Inner Brow Raiser**	Frontalis (medial)	**✓**	**X**
**AU2**	**Outer Brow Raiser**	Frontalis (lateral)	**✓**	**X**
**AU1+2**	**Brow Raiser**	Frontalis	**✓**	**✓**
**AU4**	**Brow Lowerer**	Procerus, Depressor supercilii, Corrugator supercilii	**✓**	**✓**
**AU5**	**Upper Lid Raiser**	Orbicularis oculi, Levator palpebrae superioris	**✓**	**X**
**AU6**	**Cheek Raiser**	Orbicularis oculi, pars orbitalis	**✓**	**✓**
**AU7**	**Lid Tightener**	Orbicularis oculi, pars palpebralis, Levator palpebrae superioris	**✓**	**✓**
**AU43**	**Eye closure**		**✓**	**✓**
**AU45**	**Blink**		**✓**	**✓**
**AU46**	**Wink**		**✓**	**X**
**AD47**	**Half-blink**		**X**	**✓**
**AU8**	**Lips Towards Each Other**	Orbicularis oris	**✓**	**X**
**AU9**	**Nose Wrinkler**	Levator labii superioris alaeque nasi	**✓**	**✓**
**AU10**	**Upper Lip Raiser**	Levator labii superioris	**✓**	**✓**
**AU11**	**Nasolabial Furrow Deepener**	Zygomatic minor	**✓**	**X**
**AU12**	**Lip Corner Puller**	Zygomatic major	**✓**	**✓**
**AU13**	**Cheek Puffer**	Caninus (or Levator anguli oris)	**✓**	**X**
**AU14**	**Dimpler**	Buccinator	**✓**	**✓**
**AU15**	**Lip Corner Depressor**	Depressor anguli oris	**✓**	**X**
**AU16**	**Lower Lip Depressor**	Depressor labii inferioris	**✓**	**✓**
**AU160**	**Lower Lip Relax**	Relaxation of orbicularis oris/lower lip	**X**	**✓**
**AU17**	**Chin Raiser**	Mentalis	**✓**	**✓**
**AU18**	**Lip Pucker**	Incisivii labii (superioris and inferioris), Orbicularis oris	**✓**	**✓**
**AU20**	**Lip Stretcher**	Risorius	**✓**	**X**
**AU21**	**Neck Tightener**	Platysma myoides	**✓**	**X**
**AU22**	**Lip Funneler**	Orbicularis oris	**✓**	**✓**
**AU122**	**Lower Lip Inner Curl**		**X**	**✓**
**AU222**	**Lower Lip Extension**		**X**	**✓**
**AU23**	**Lip Tightener**		**✓**	**X**
**AU24**	**Lip Pressor**		**✓**	**✓**
**AU25**	**Lips Parted**	Orbicularis oris, Levator labii superioris, Depressor labii inferioris, non-mimetic muscles	**✓**	**✓**
**AU26**	**Jaw Drop**		**✓**	**✓**
**AU27**	**Mouth Stretch**		**✓**	**✓**
**AU28**	**Lip Suck**	Orbicularis oris	**✓**	**✓**
**AU38**	**Nostril Dilator**	Nasalis	**✓**	**✓**
**AU39**	**Nostril Compressor**	Nasalis, Depressor septi nasi	**✓**	**✓**
**AU138**	**Nose Shield Expander**	Nasalis	**X**	**✓**
**AU139**	**Nose Shield Flattener**	Nasalis	**X**	**✓**
**AU238**	**Nose Downwards**	Nasalis, Depressor septi nasi	**X**	**✓**

**Table 4:** Comparison between FACS Action Descriptors (AD) for humans [2] and gorillas. ✓- present, x—absent. Cells highlighted in grey are present in gorillas.

AU/AD code	AU/AD name	Human	Gorilla
**AD101**	**Scalp Retraction**	**X**	**✓**
**AD19**	**Tongue Show**	**✓**	**✓**
**AD190**	**Tongue Downwards**	**X**	**✓**
**AD191**	**Tongue Curl**	**X**	**✓**
**AD119**	**Lick**	**✓**	**✓**
**AD29**	**Jaw Thrust**	**✓**	**✓**
**AD30**	**Jaw Sideways**	**✓**	**✓**
**AU31**	**Jaw Clencher**	**✓**	**X**
**AD32**	**Bite**	**✓**	**✓**
**AD33**	**Blow**	**✓**	**X**
**AD34**	**Puff**	**✓**	**✓**
**AD35**	**Suck**	**✓**	**X**
**AD36**	**Bulge**	**✓**	**✓**
**AD37**	**Lip Wipe**	**✓**	**✓**
**AD40**	**Sniff**	**✓**	**✓**
**AD50**	**Vocalisations**	**✓**	**✓**
**AD80**	**Swallow**	**✓**	**X**
**AD81**	**Chewing**	**✓**	**✓**

### Action Units

As in previous FACS adaptations for new species [e.g., 12], we report each AU found in gorillas, providing a numerical code, a descriptive name, and a brief comparison of the anatomical features between humans and gorillas, and, where relevant, other primates. The following information is included for each AU:

**A. Proposed muscular basis:** Muscle(s) that produces the AU (**Table 3**);

**B. Appearance changes:** List of multiple and redundant cues (e.g., face feature movement of shape change, movement direction, and formation or deepening of wrinkles, in relation to facial landmarks, **Figs 3** and **4**) that help to identify when an AU occurs. Video (see **SI Videos**) and photo examples are also presented illustrating different appearance changes;

**C. Minimum criteria** to code an AU: visible appearance change(s) that when present are sufficient to code an AU;

**D. Subtle differences between AUs:** Wherever necessary, a comparison of similar AUs that can be confused or that share some appearance changes.

#### Upper face Action Units

*AU1+2—Brow Raiser*. The brow area exhibits significant differences between humans and gorillas, which influence the upper face AUs and their respective appearance changes. In **humans**, the forehead (the portion of the frontal bone between the eyebrows and hairline) and the eyebrows (hair strips located on the supraorbital ridges) are unique morphological and anatomical features. The eyebrows are highly visually prominent on the bare forehead and contribute to important appearance changes for several AUs. In contrast, **gorillas** lack a forehead and eyebrows; instead, the area of the head located on the frontal bone is referred to as the frontal region, and the prominent area above the eyes is known as the browridge. However, the frontal region is not above the supraorbital ridges, but instead sits almost on an oblique plane in relation to the face. The frontal region is usually covered in dense hair which prevents detecting any appearance changes (e.g., wrinkles) other than movement of the hair. The browridge is much more salient than in humans, and presents two slightly salient arches separated by the glabella. These arches are similar anatomical features to other species (e.g., chimpanzees) and move upwards and downwards.

**Fig 7:**
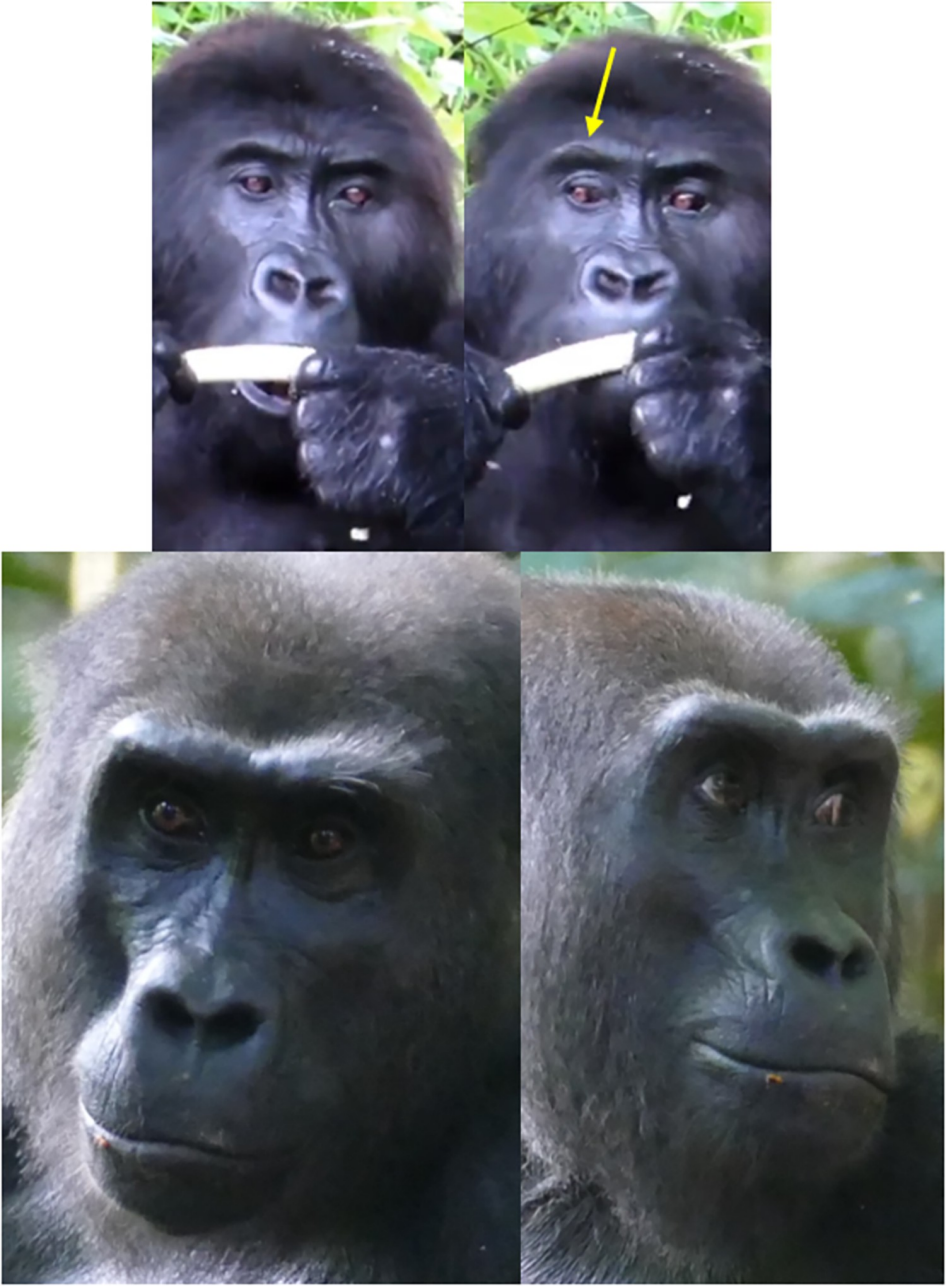
Examples of AU1+2 –Brow Raiser. Top left: neutral browridge. Top right: AU1+2R - Action Unit 1+2—Brow Raiser Right. The right brow ridge area is pulled up, but not the left brow ridge area. The right brow ridge area is also only pulled up on the upper part of the superciliary arch (indicated by the yellow arrow). Bottom left: neutral browridge. Bottom right: AU1+2. Top pictures: still frames from gracegorillas.org videos, bottom pictures by MR.

In the human FACS, AU1, the "Inner Brow Raiser," elevates the medial portion of the eyebrow, and AU2, the "Outer Brow Raiser," elevates the lateral section, creating wrinkles on the forehead. These two movements can be coded separately in humans, and unilateral movements (i.e., occurring on one side of the face only) are often observed. In gorillas, similar to other primates, AU1 and AU2 do not occur independently. Instead, they are only observed as a combined movement, denoted as AU1+2 (e.g., **S1a-S6a and S1b-S6b Videos in S1 Videos zip target**). AU1+2 can be observed unilaterally in gorillas, i.e., on only one side of the browridge (i.e., R for right side of the face and L for left side of the face, see **S3a and S3b Video in S1 Videos zip target**). The upwards brow movement in gorillas is not always uniform along the whole width of the browridge, sometimes with upwards movement more pronounced in the upper and outer area of the arch (e.g., **Fig 7**).

**A. Proposed muscular basis:** Frontalis.

**B. Appearance changes**:

1. The browridge moves dorsally. This movement can be detected at least in three different ways:
  1.1. The salient arches move fully up (e.g., **S5a and S5b Video in S1 Videos zip target**) or have portions pulled up (e.g., **S3a and S3b Video in S1 Videos zip target**);
  1.2. The skin slides over the browridge (e.g., **S2a, S2b, S6a, and S6b Videos in S1 Videos zip target**);
  1.3. The entire browridge itself is pulled upwards, including glabella (e.g., **S1a and S1b Video in S1 Videos zip target**).
2. The portions of the browridge or the whole browridge that is pulled upwards may appear flattened and less salient.
3. The underbrow region is more visible, with skin appearing to stretch and the eye cover fold may be more exposed, particularly if 1.3. happens.
4. If no hair is present in the frontal region and the skin is visible, wrinkles appear in this area or the existent wrinkles are deepened and brought closer together (e.g., **S2a and S2b Video in S1 Videos zip target**).
5. In more intense movements, the hair in the frontal region might move dorsally.
6. In profile view, even if most of the upper face is not visible, an upwards movement of the browridge may be visible and thus, AU1+2 can be coded.
7. Unilateral movements (L or R) can be observed, as both sides of the browridge can move independently (e.g., **Fig 7**, and **S3a and S3b Video in S1 Videos zip target**).

**C. Minimum criteria**: Dorsal movement of the any portion of the browridge.

**D. Subtle differences between AUs:** Head movements (e.g., head up) or changes in camera angle might make the underbrow more visible (appearance change 3). As a result, it may appear that AU1+2 is occurring. However, this movement should only be coded if the minimum criterion is met, specifically if the browridge is observed moving upward.

**Fig 8:**
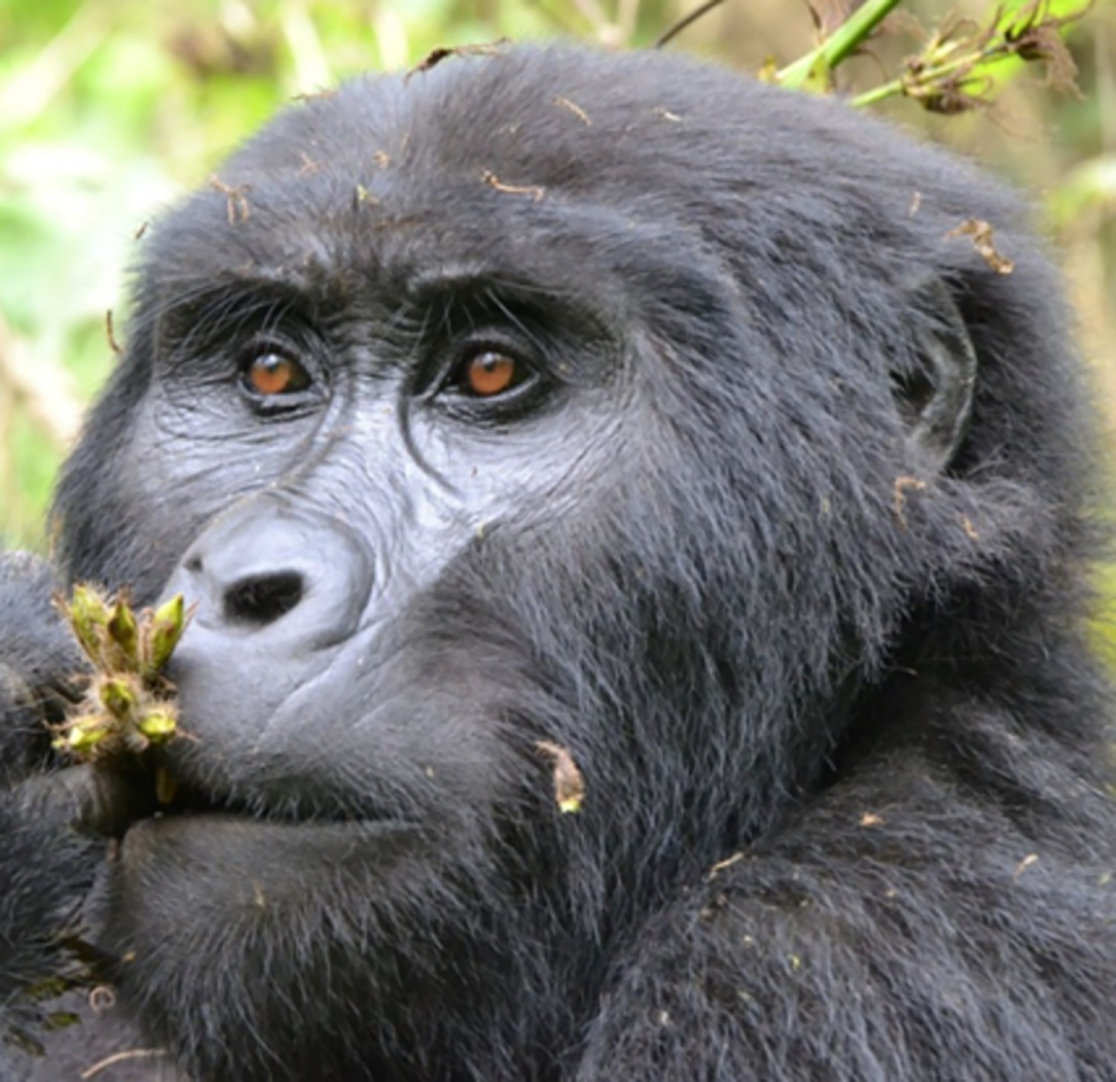
Brow arches in a female *G*. *b*. *beringei* with some thin long hairs directed downwards. Picture by RC.

*AU4—Brow Lowerer*. The glabella area (i.e., area between the brows) differs slightly between humans and gorillas. While in **humans** the glabella is a flat area of smooth skin between the brows, in **gorillas** the glabella area usually has permanent wrinkles, and may sit deeper than the rest of the browridge or the salient arches of the browridge. In **humans**, the glabella contrasts with the surrounding areas due to being a (usually) hairless area between two strips of hair, while in **gorillas** either the whole browridge is covered in hair or is fully naked (although see **Fig 5** for examples of variation in this). In particular, *G*. *b*. *beringei* may present more or less individualised brow arches and thin long hairs projected inferiorly in front of the eyes, attached to the arches (**Fig 8**) whilst other subspecies do not seem to present this feature (**Fig 5**).

**Fig 9:**
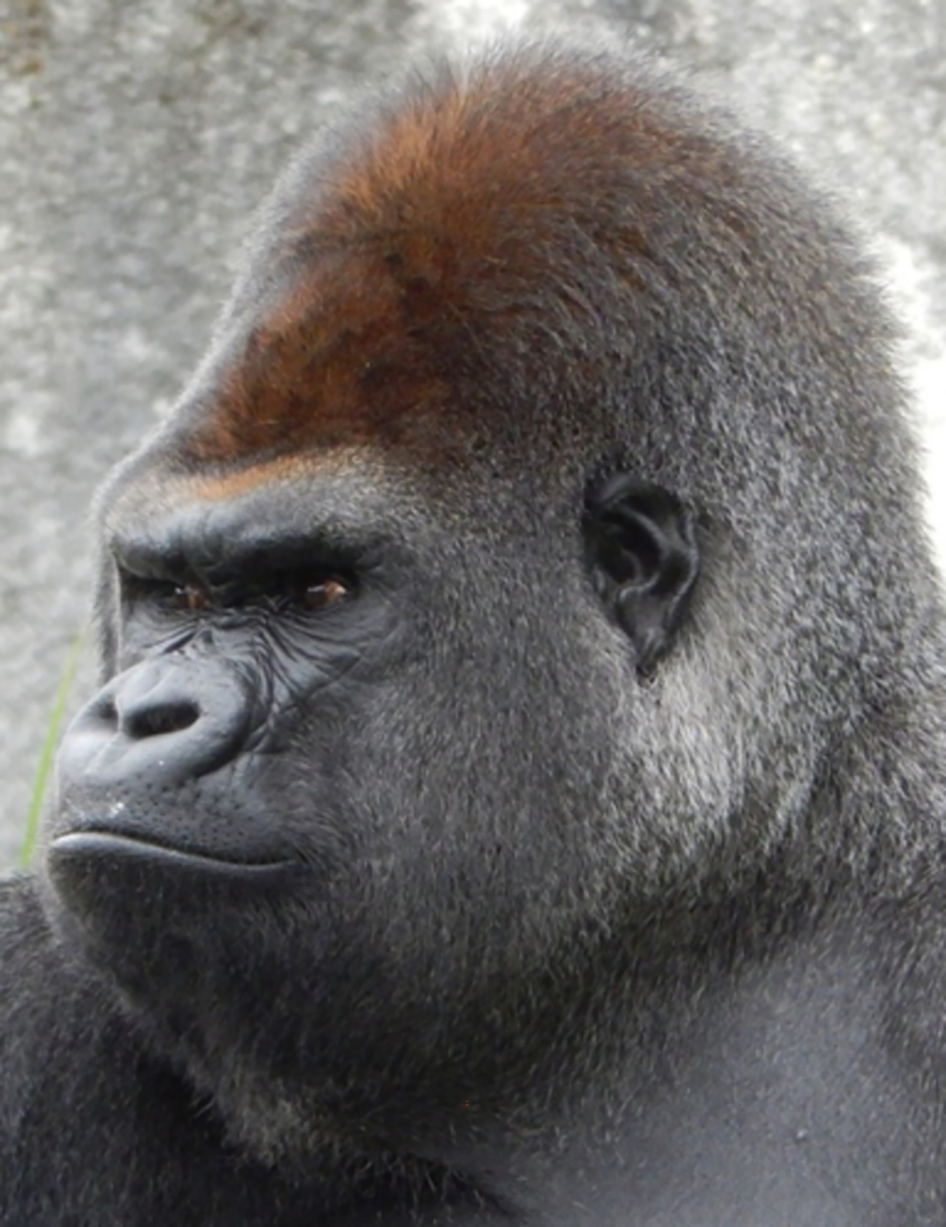
Intense AU4—Brow Lowerer. Other AUs present. Picture by jadorelyon user from Pixabay.com.

**Fig 10:**
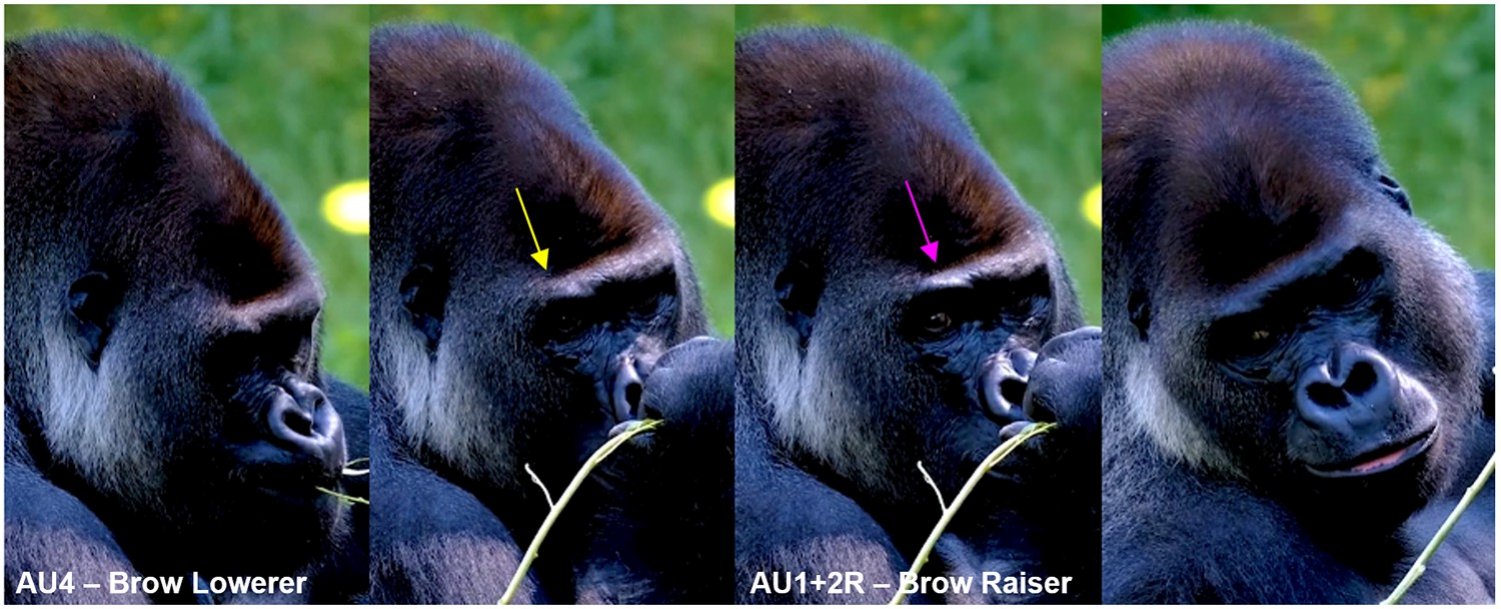
Sequence of alternating movements between AU4—Brow Lowerer and AU1+2—Brow Raiser.

**Humans** can produce AU4—Brow Lowerer, by the contraction of three different muscles (procerus, depressor supercilii, and corrugator). In **humans**, the procerus pulls the medial end of the eyebrow downward, the depressor supercilii pulls the eyebrow and the skin above the orbit downward, and the corrugator supercilii pulls the eyebrows towards the midline and downwards. In **gorillas**, these three muscles are also present producing some of the same appearance changes seen in humans, except for corrugation. Corrugation of the brows, i.e., the brows come closer together and create wrinkles on the glabella, seems to be unique to humans. Hence, gorillas produce AU4—Brow Lowerer, but with slightly different appearance changes (e.g., **Figs 9 and 10, S7a-S10a and S7b-S10b Videos in S1 Videos zip target**).

On the first image from the left, the individual presents an AU4; On the second image, the individual’s browridge returned to neutral; On the third image the individual produces and AU1+2R, in which the right browridge arch is pulled upwards; On the fourth image, the browridge returns to neutral again. Other AUs present. Still frames from **S11 Video in S1 Videos zip target**.

**A. Proposed muscular basis:** Procerus, depressor supercilii, and corrugator supercilii.

**B. Appearance changes**:

1. The browridge moves caudally, with either the browridge moving down (e.g., **S7a and S7b Video in S1 Videos zip target**), the skin of the browridge moving down (e.g., **S8a and S8b Video in S1 Videos zip target**), or any portion of the brow arches (usually the inner portions) moving down (e.g., **S8a and S8b Video in S1 Videos zip target**).
2. If only the inner corners of the brow arches are pulled down, a slight "V" shape may form.
3. Wrinkles may appear or may be deepened on the glabella and root of the nose.
4. The eye aperture may be narrowed.
5. In more intense movements, the underbrow region may become less visible, the eye cover fold may disappear from view, and the root of the nose may also be covered (e.g., **Fig 9**).
6. If hair is present above or on the browridge, it will move downwards.
7. In very intense movements, the root of the nose might be completely covered (e.g., **S7a and S7b Video in S1 Videos zip target**).

**C. Minimum criteria**: Caudal movement of the browridge, glabella, or brow arches.

**D. Subtle differences between AUs:** Even though AU1+2 and AU4 act in opposite directions (dorsal and caudally respectively), in **humans** they can be coded simultaneously, impacting each other’s appearance changes and creating new appearance changes. In contrast, in **gorillas** they are mutually exclusive, i.e., they cannot be coded simultaneously, and hence do not share any appearance changes. In gorillas, either an AU1+2 is acting whenever the browridge goes upwards from neutral, or an AU4 is acting whenever the browridge goes downwards from neutral. However, these two movements are sometimes observed in succession, in which AU1+2 is immediately followed by AU4 (or vice-versa), without a clear temporal return to neutral (e.g., **Fig 10**). In addition, the release of AU4 to neutral might be difficult to distinguish from a weak AU1+2, and the release of AU1+2 to neutral might be confused with a weak AU4. However, the release of AU1+2 (e.g., **S4a, S4b, S10a, S10b, S11a, and S11b Videos in S1 Videos zip target**) should not be coded as AU4, and vice-versa. In order to define when to code one or the other, comparison with the neutral browridge for each individual may be necessary, as well as frame-by-frame analysis of the succession of movements.

*AU6—Cheek Raiser and AU7—Lid Tightener*. **Human** cheeks are formed by deposits of fat sitting on the zygomatic bones, just below the eyes. The outer layer of the muscle surrounding the eyes (orbicularis oculi pars orbitalis) contracts to pull the surrounding skin towards the eye, decreasing the infraorbital triangle (IOT, e.g., **Fig 3**) area and raising the cheeks. In **gorillas**, there are no evident fat deposits on the zygomatic bone that form the human-like cheeks, so the appearance changes are significantly different from humans, instead affecting the skin around the eye more globally in gorillas. Likely due to these anatomical differences, in **humans** AU6 can be coded as an independent action, while in **gorillas** it is mostly observed with AU43—Eye closure or AU45—Blink (see below for appearance changes).

**Fig 11:**
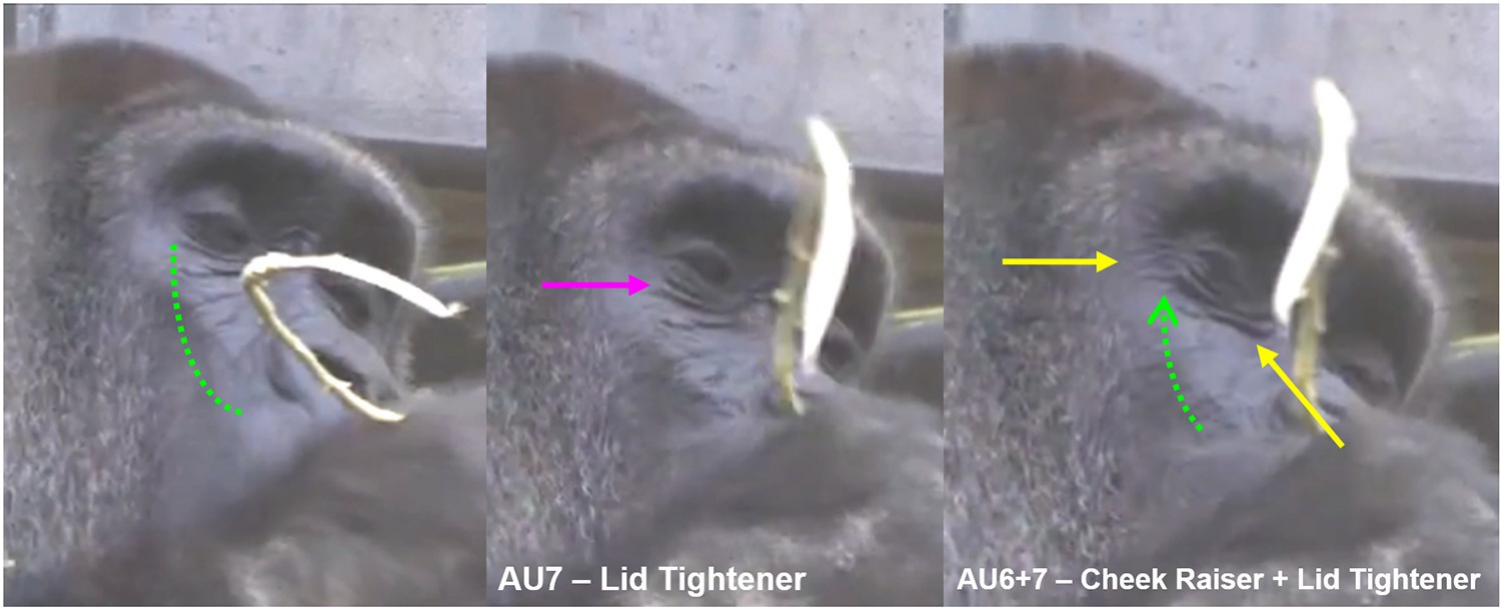
Sequence of AU6—Cheek Raiser and AU7—Lid Tightener. Left: Neutral under eye area; Centre: AU7—Lid Tightener, identified by the following appearance changes: lower eyelid bulging, infraorbital furrow deepened (indicated by pink arrow), and narrowing of the eye; Right: AU6+7—Cheek Raiser and Lid Tightener, with IOT reduced in size (indicated by dashed green arrow), and skin wrinkles under the eye and at the eye corner appearing or being deepened (indicated by yellow arrow). Still frames from **S12 Video in S1 Videos zip target**.

**Fig 12:**
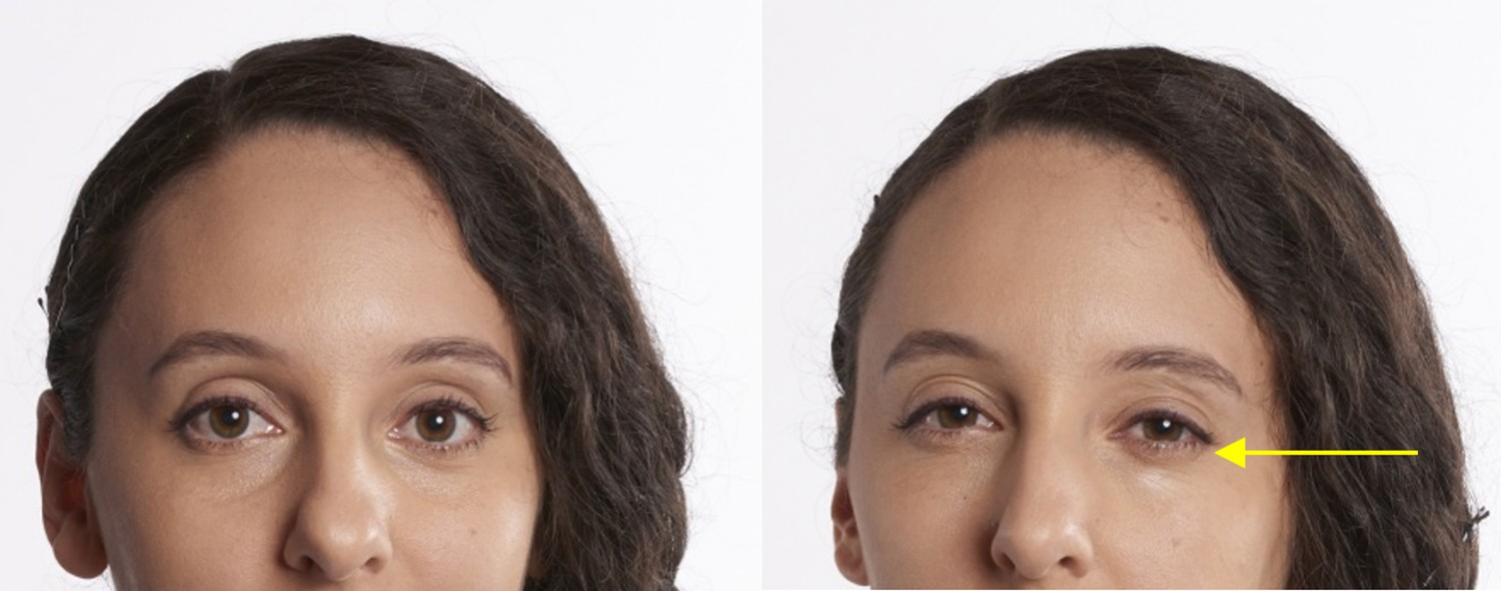
Example of AU7—Lid Tightener in humans. Left: Neutral eyes. Right: AU7 with several appearance changes visible in the lower eyelid: the lower eyelid is raised covering more of the eyeball than in neutral, narrows eye aperture, the shape of the lower eyelid becomes less curved, the lower eyelid furrow becomes deeper. Picture by Robin Higgins user from Pixabay.com.

In **gorillas**, AU6 was also observed together with AU7—Lid Tightener (lower eyelid is raised or bulged, e.g., **Fig 11, S12a, S12b, S13a, and S13b Videos in S1 Videos zip target**), which in humans can also be coded as an independent AU (e.g., **Fig 12**). However, in gorillas, AU7 may be too hard to detect reliably due to the difficulty in clearly visualising the eye area, and was only observed once in the video analysed here. Hence, we recommend to code this movement only in optimal conditions of visibility of the eye area and when the eye is opened.

**A. Proposed muscular basis:** Orbicularis oculi (pars orbitalis).

**B1. Appearance changes for AU6**:

1. Wrinkles form around the eye, particularly at the eye corner and under the eye.
2. The IOT decreases in size and bulges (e.g., **Fig 11, S12a and S12b Video in S1 Videos zip target**).
3. The skin of the IOT is pulled towards the eye.
4. Deepens the infraorbital furrow.
5. Even if eye closure is not present, it narrows eye aperture.

**B2. Appearance changes for AU7**:

1. Lower eyelid bulges, infraorbital furrow deepens, and eye aperture narrows (e.g., **Fig 11**).
2. When eye closure (AU43) or AU7 are present together with AU6, it can make the eyelids appear compressed and bulging.
3. When AU6+AU43 are acting simultaneously, it is not possible to code AU7 as the appearance changes will be masked by the previous AUs.

**C1. Minimum criteria for AU6**: Wrinkles form around the eyes and eye narrows.

**C2. Minimum criteria for AU7:** Eye aperture is narrowed, lower eyelid is raised, and bulges; If eyes are closed (without AU6), eyelids appear compressed and bulging.

*AU43—Eye closure and AU45 –Blink*. In the human FACS, the distinction between blinks (AU45) and eye closure (AU43) is based on duration. AU45 is coded if the eye is closed for half a second or less, while AU43 codes varying intensities of the lower eyelid moving over the eyeball to close the eye, with AU43E (E denoting maximum intensity) indicating complete closure (upper eyelid touching lower eyelid and covering the eyeball entirely). Unlike other AUs, where a muscle contracts to produce movement, eye closure or blinking by AU43/45 is achieved by the relaxation of the levator palpebrae superioris muscle.

**A. Proposed muscular basis:** Orbicularis oculi (pars palpebralis) and levator palpebrae superioris relaxation.

**B. Appearance changes**:

1. The eyelids move towards each other, with usually more movement from the upper eyelid, reducing the eye opening until it closes the eye completely (i.e., the eyelids cover the eyeball completely).
2. The eyelids become more visible.
3. When the eyelids touch each other closing the eye there may be some additional bulging and wrinkling on the eyelids from pressing eyelids against each other can be seen, or movement right below the eye.
4. Subtle movement or tension on the skin might be seen globally around the eyes, including browridge, IOT and both eye corners (e.g., **S14a and S14b Video in S1 Videos zip target**).
5. All appearance changes above can be observed in both AU43 and AU45. However, in **AU43** the eye remains closed for 500ms or more (e.g., **S15 Video in S1 Videos zip target**), while in **AU45** the eye opens within 500ms (e.g., **S14a** and **S14b Video in S1 Videos zip target**). Therefore, AU43 and AU45 are mutually exclusive, as they cannot be coded simultaneously.

**C. Minimum criteria**: the eyelids move towards each other (or only upper eyelid) and cover the eyeball completely.

**D. Subtle differences between AUs:** In the human FACS, appearance changes 3 and 4 are part of AU6—Cheek Raiser and AU7—Lid Tightener, respectively. AU6 and AU7 were not observed in gorillas in isolation, and the appearance changes of AU6 and AU7 are usually accompanied by AU43/45, consider coding AU6 as well, whenever the IOT is shortened or bulged, and/or AU7 if the visibility of the lower eyelid is good enough to detect the appearance changes. However, do not code AU7 if the eye is closed and the lower eyelid shape is not visible, as this is one of the minimum criteria to identify AU7.

*AU47—Half-Blink*. The AU47 –The Half-Blink has not been described by Ekman and colleagues [2] in the **human** FACS, but has been described for the domestic cat in the CatFACS [15], for horses in the EquiFACS [14], and for the common marmosets in the CalliFACS [12]. In the domestic cat, this movement is frequently observed as part of the behavioural repertoire, and is described as sequential movements of the eyelids towards and away from each other, without ever closing the eye completely. A similar eyelid movement has been observed in other species outside FACS (e.g., dogs: [82]) as a single movement instead of a sequence of movements. Although the function of this movement in gorillas is still not clear, in humans it has recently been linked to differences in spontaneous versus voluntary movements [83] and in cats seems to function in communicating positive emotion towards humans [21]. In **gorillas** the single AU47—Half-Blink, is described below (e.g., **S16a and S16b Video in S1 Videos zip target**).

**A. Proposed muscular basis:** Orbicularis oculi (pars palpebralis) and levator palpebrae superioris relaxation.

**B. Appearance changes**:

1. The upper eyelid moves towards the lower eyelid, reducing the eye opening and returning to neutral without ever touching the lower eyelid.
2. In some movements, both upper and lower eyelids are seen moving towards and away from each other.

**C. Minimum criteria**: the eyelids move towards each other (or only upper eyelid), but do not cover the eyeball completely (eyelids might touch near the eye corners, but not medially).

**D. Subtle differences between AUs:** AU47 is coded when the eyelids move towards each other without fully covering the eyeball, whereas AU43/45 is coded when the eyeball is completely covered by the eyelids. With AU47, no movement around the eye appears to be displayed. Therefore, if any further movement is detected in the browridge or infraorbital triangle (IOT), consider coding AU4 or AU6, respectively. Additionally, slight eyelid movements may occur when the individual changes eye direction, but this should not be coded as AU47.

#### Lower Face Action Units

**Fig 13:**
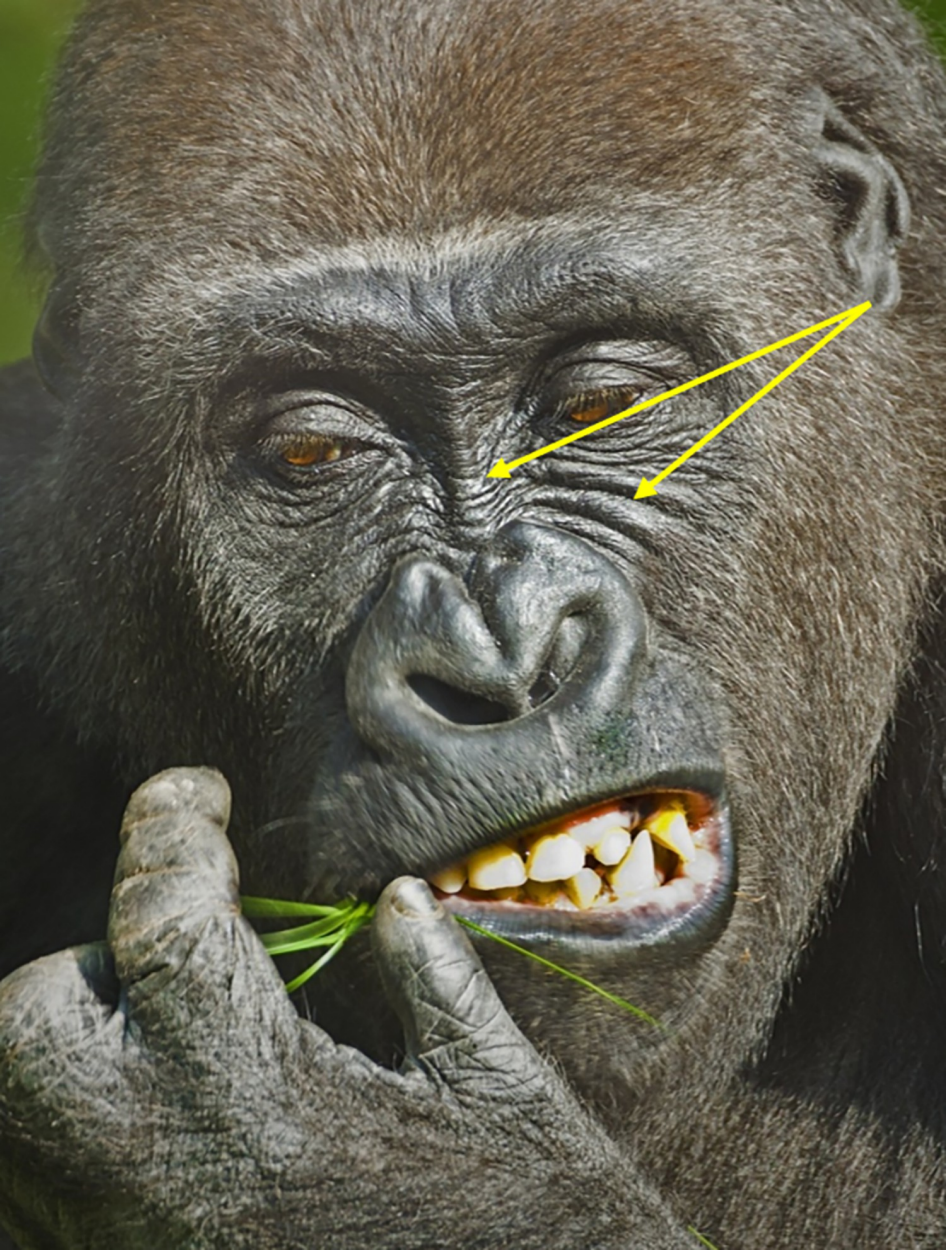
AU9L - Left Nose Wrinkler. Yellow arrow indicates deepened wrinkles above the nose shield and extending into the IOT on the left side of the face. The IOT is also bulging. Appearance changes of AU9 are visible on the left side of the face, particularly when comparing to the right side of the face that does not have an AU9. AU10L is also present on the left side of the face (note lip shape and shortening of the lip medially—see next section for AU10 appearance changes) along other AUs. Picture by GerMai user from Pixabay.com.

*AU9—Nose Wrinkler*. In **humans**, the levator labii superioris alaeque nasi muscle wrinkles the nose. In **gorillas**, this movement is very similar with most of the same appearance changes (e.g., **Fig 13, S17a-S19a and S17b-S19b Videos in S1 Videos zip target**). In both species, smaller movements produce more appearance changes around the nostril area, pulling the nostrils upwards, whilst in more intense movements the nostril, side of nose, upper lip and glabella may have visible changes.

**A. Proposed muscular basis:** Levator labii superioris alaeque nasi.

**B. Appearance changes**:

1. The nostril wings are pulled upwards, and change shape (widening or narrowing).
2. New wrinkles form on the nose above the nostrils, and the existent wrinkles deepen; the wrinkles run laterally sometimes extending into the IOT (e.g., **S17a** and **S17b Video in S1 Videos zip target**).
3. Both the lower eyelid furrow and the infraorbital furrow deepen.
4. The skin bulges on the IOT.
5. The upper lip may be pulled upwards but this is clearly following the nose movement, without any asynchrony between nose and upper lip movements upwards (e.g., **S18a and S18b Video in S1 Videos zip target**).
6. In strong movements, the browridge may be lowered, but the movement is mostly seen in the glabella area.
7. The teeth may become exposed.
8. Unilateral movements (L or R) are often observed, with only one nostril or one side of the nose shield being pulled upwards (e.g., **Fig 13,** and **S19a and S19b Video in S1 Videos zip target**).

**C. Minimum criteria**: the nostril wings are pulled upwards or the nose shield moves upwards.

**D. Subtle differences between AUs:** In very strong movements of AU9, the eye aperture may narrow, the glabella may be pulled ventrally, and wrinkles may form on the nose and extend laterally from it. In these instances, some appearance changes of AU9 may overlap with AU4; if the minimum criteria for AU4 are met, it should also be coded. Additionally, in very strong AU9, AU6 might be present due to the constriction of the eye. Therefore, AU6 should also be coded in such cases.

*AU10—Upper Lip Raiser*. In **humans**, AU10 is an up/down movement, due to the positioning of the muscle and the direction of contraction. In **gorillas**, this movement produces similar appearance changes to the ones observed in humans, with slight changes due to the prognathism of the mouth area and the lack of lip eversion with high contrast in coloration between the everted lips (e.g., **S20a-S22a and S20b-S22b Videos in S1 Videos zip target**).

**A. Proposed muscular basis:** Levator labii superioris.

**Fig 14:**
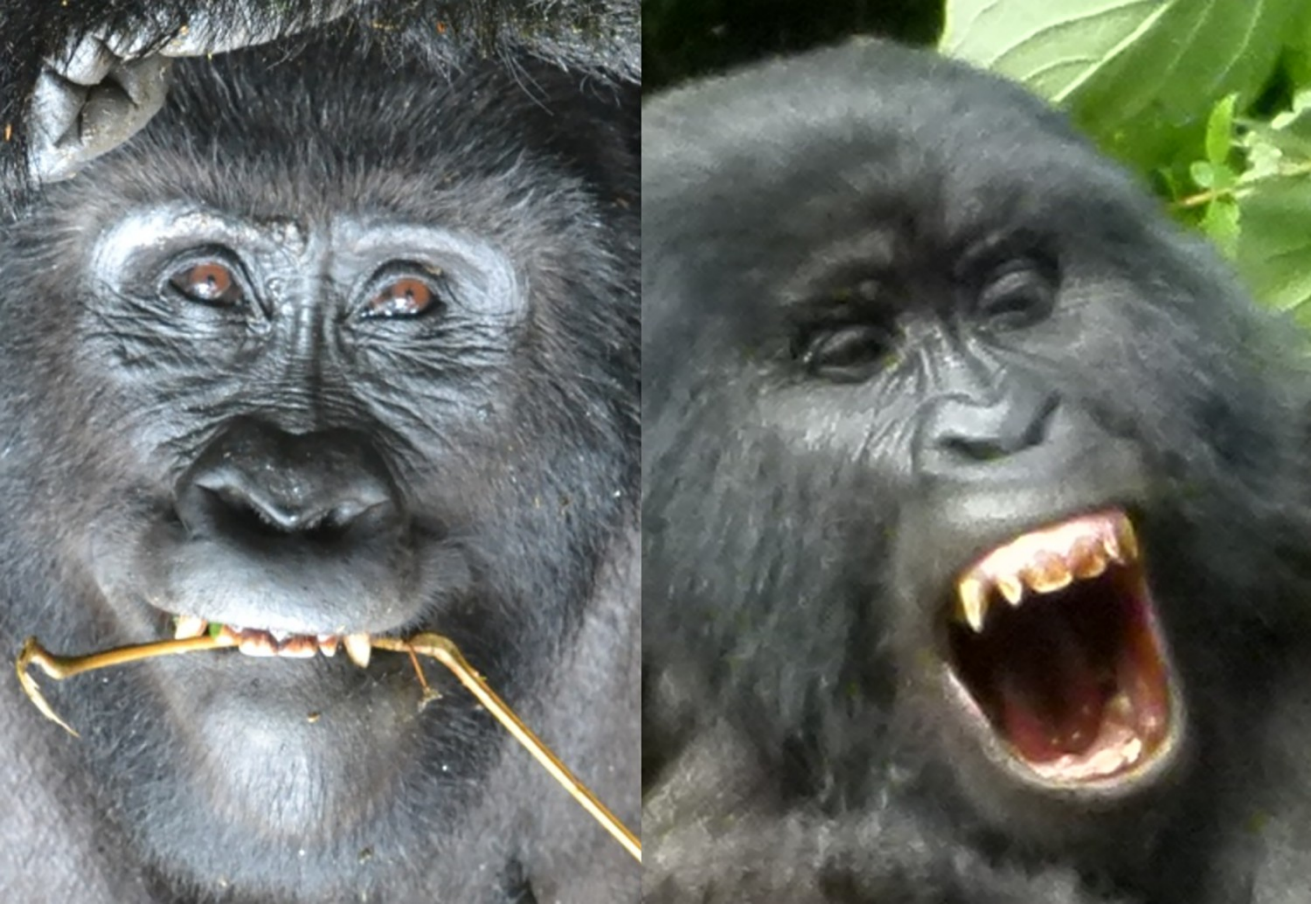
AU10—Upper Lip Raiser. Other AUs present, including on the left picture an AU28 –Lips Suck (see below). Pictures by RC and MR.

**B. Appearance changes**:

1. The upper lip is pulled upwards. This movement may happen globally along the upper lip (e.g., **S20a and S20b Video in S1 Videos zip target**) or on a more localised area, such as a unilateral movement or a medial movement (e.g., **S21a and S21b Video in S1 Videos zip target**)
2. The distance between the nose and the upper lip decreases.
3. The lip appears to thicken at the edge as it is retracted.
4. The inside of the lip may become visible and the upper teeth are revealed.
5. The nose shield may follow the movement of the upper lip, particularly at the basal area (e.g., **S22a and S22b Video in S1 Videos zip target**).
6. In stronger actions of AU10, the subnasal furrow deepens as the lip is pulled upwards.
7. In stronger actions, as the lip is fully retracted, the nose shield may be pushed upwards, but no wrinkling of the nose or IOT is visible.
8. In extreme actions, the lip is fully retracted revealing both the upper teeth (e.g., **Fig 14**) and gums.
9. In profile, the top lip is seen to be pulled up towards the nose and in stronger actions the lip may appear to be slightly bulging.

**C. Minimum criteria**: The upper lip is pulled upwards at any point along its width or the whole lip is pulled upwards.

**D. Subtle differences between AUs:** To distinguish between a strong AU9 that may raise the upper lip from an AU10, it is necessary to exclude any asynchronous nose shield or nostril movement and wrinkling of the nose. If any of these appearance changes are present, AU9+10 is coded. If not, only AU10 is coded.

**Fig 15:**
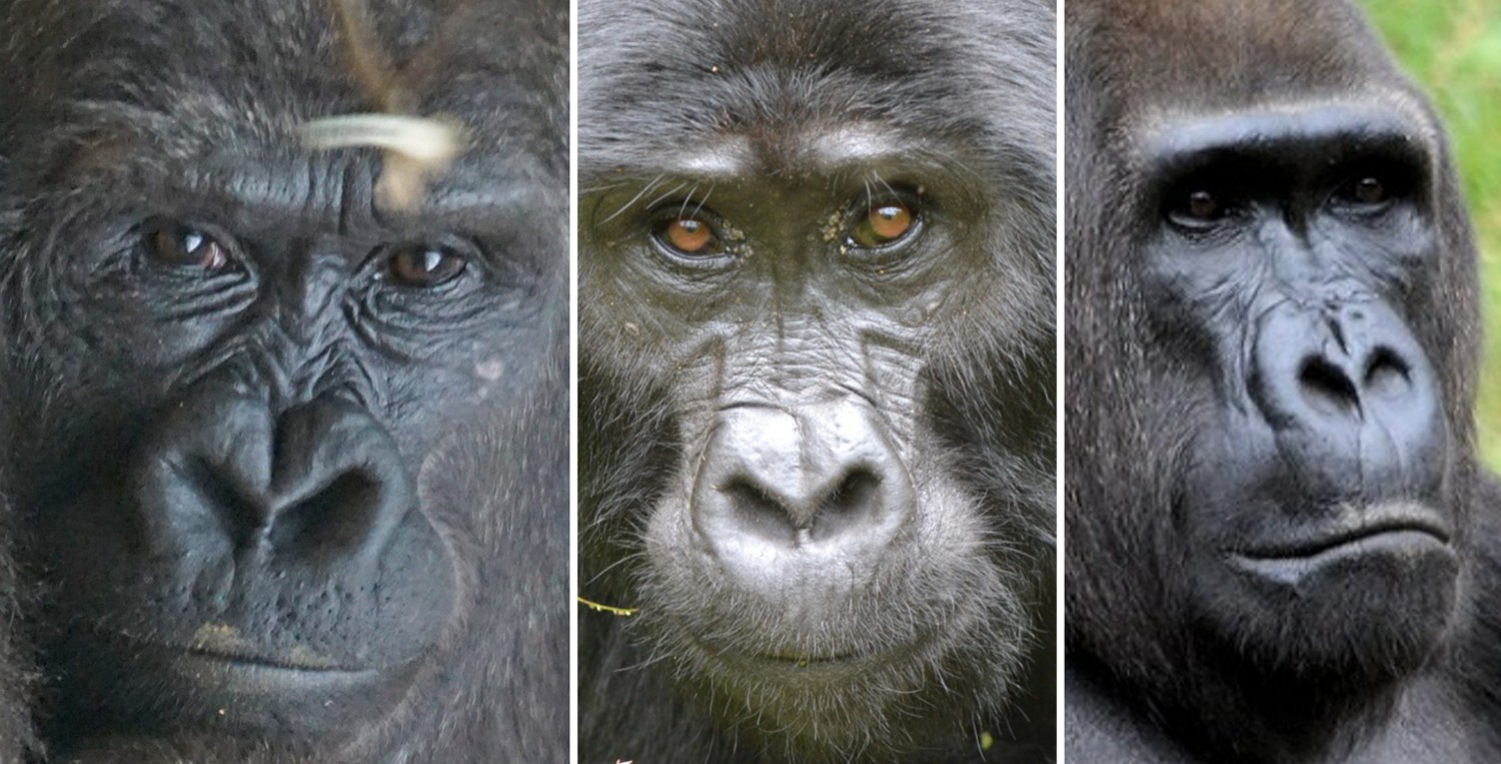
Examples of lip corners visibility in gorillas. Due to the prognathism of the mouth area, the lip corners are not visible on the picture on the left and centre in frontal view, but one of the lip corners is visible on the picture on the right in which the face was photographed at a 45° angle. Pictures by Pixel-mixer user (left) and PublicDomainPictures user (right) from Pixabay.com, and by RC (centre).

*AU12—Lip Corner Puller*. In **humans**, the lip corners are a clearly visible landmark on the face, both in frontal and profile views, partly due to the flat morphology of the human face, and partly due to the lip eversion and contrast coloration with the surrounding skin. Hence, lip corner movements are usually very conspicuous in humans. However, in **gorillas**, the mouth area is projected forwards due to the skull prognathism. As such, the lips curve around this area resulting in the lip corners not being visible in a frontal view. The dark skin of gorillas, the lack of lip eversion, and hair covering lateral areas of the face, all further contribute to the difficult detection of movements of the lip corners. Lip corner movements are thus usually only detected in gorillas in a face profile view or at a 45° angle, and with good lighting conditions (e.g., **Fig 15**).

One of the movements produced by the lip corners is called Lip Corner Puller—AU12, and is produced by the zygomaticus major muscle, both in **humans** and **gorillas**, pulling the lip corners back and slightly upwards in the direction of the ears (e.g., **S23a-S26a and S23b-S26b Videos in S1 Videos zip target**).

**A. Proposed muscular basis:** Zygomaticus major.

**Fig 16:**
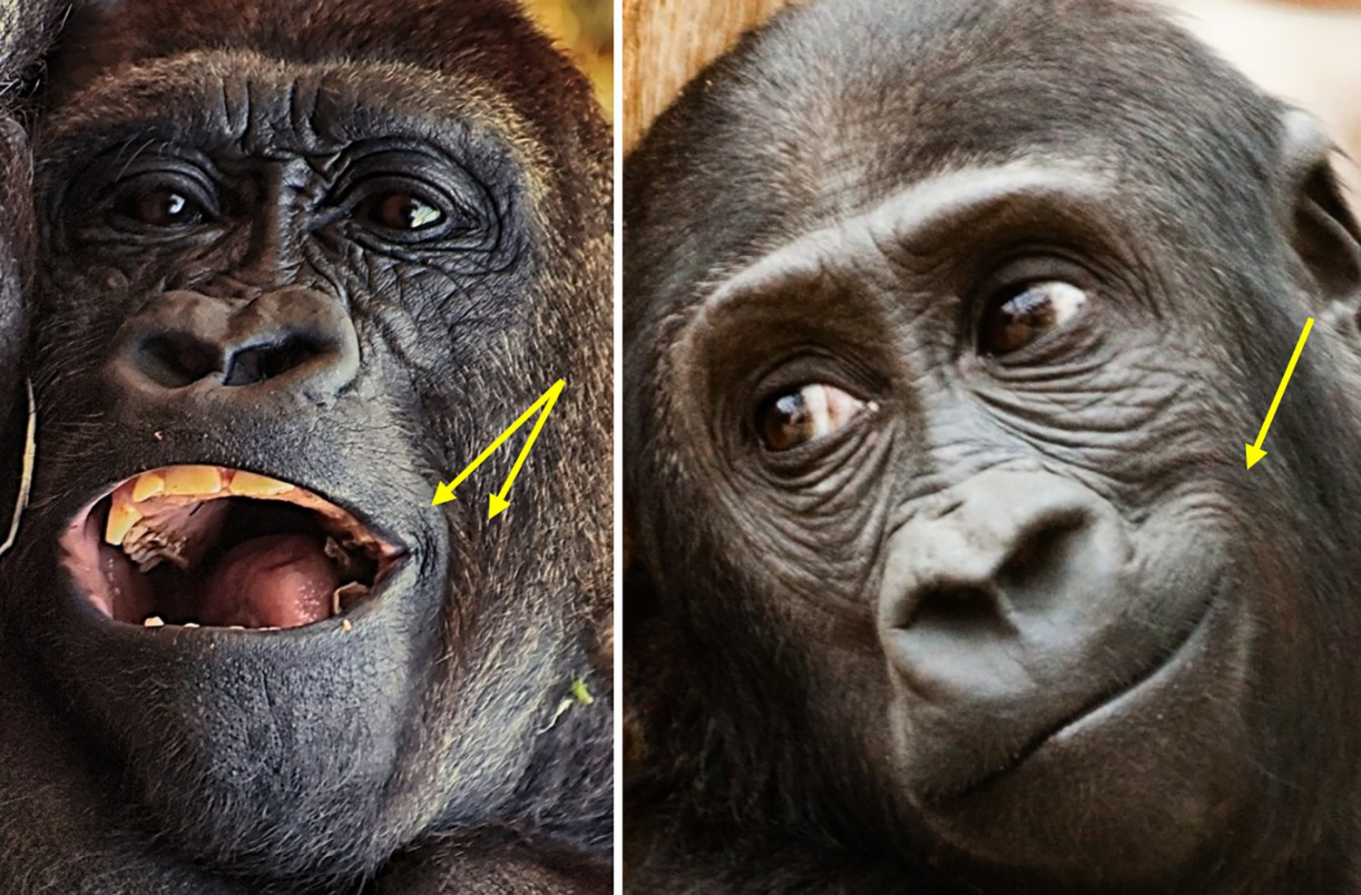
AU12—Lip Corner Puller. Yellow arrows indicate concentric wrinkles formed at the mouth corners and on the edge of the mouth region covered with hair. Left: Other AUs are present other than AU12 (AU25+27+10). Right: AU12 with closed mouth. Pictures by Alexas_Fotos user (left) and PublicDomainPictures user (right) from Pixabay.com.

**B. Appearance changes**:

1. The lip corners are pulled backwards and slightly upwards towards the ears.
2. Concentric wrinkles and bunching of the skin forms behind the lip corners and just outside the edge of the mouth area (e.g., **Fig 16**). However, in low intensity movements, no wrinkles are seen (e.g., **S23a and S23b Video in S1 Videos zip target**).
3. Both subnasal and nasolabial furrows deepen, with the nasolabial furrow being pulled laterally.
4. In some movements, the upper lip and soft nose shield may accompany the movement of the lip corner and nostrils may become wider (e.g., **S24a and S24b Video in S1 Videos zip target**).
5. Movement of the hair posterior to the lip corners may also accompany the movement.
6. The lips may part in more intense movements and expose the upper teeth.

**C. Minimum criteria**: The lip corners are pulled backwards.

**Fig 17:**
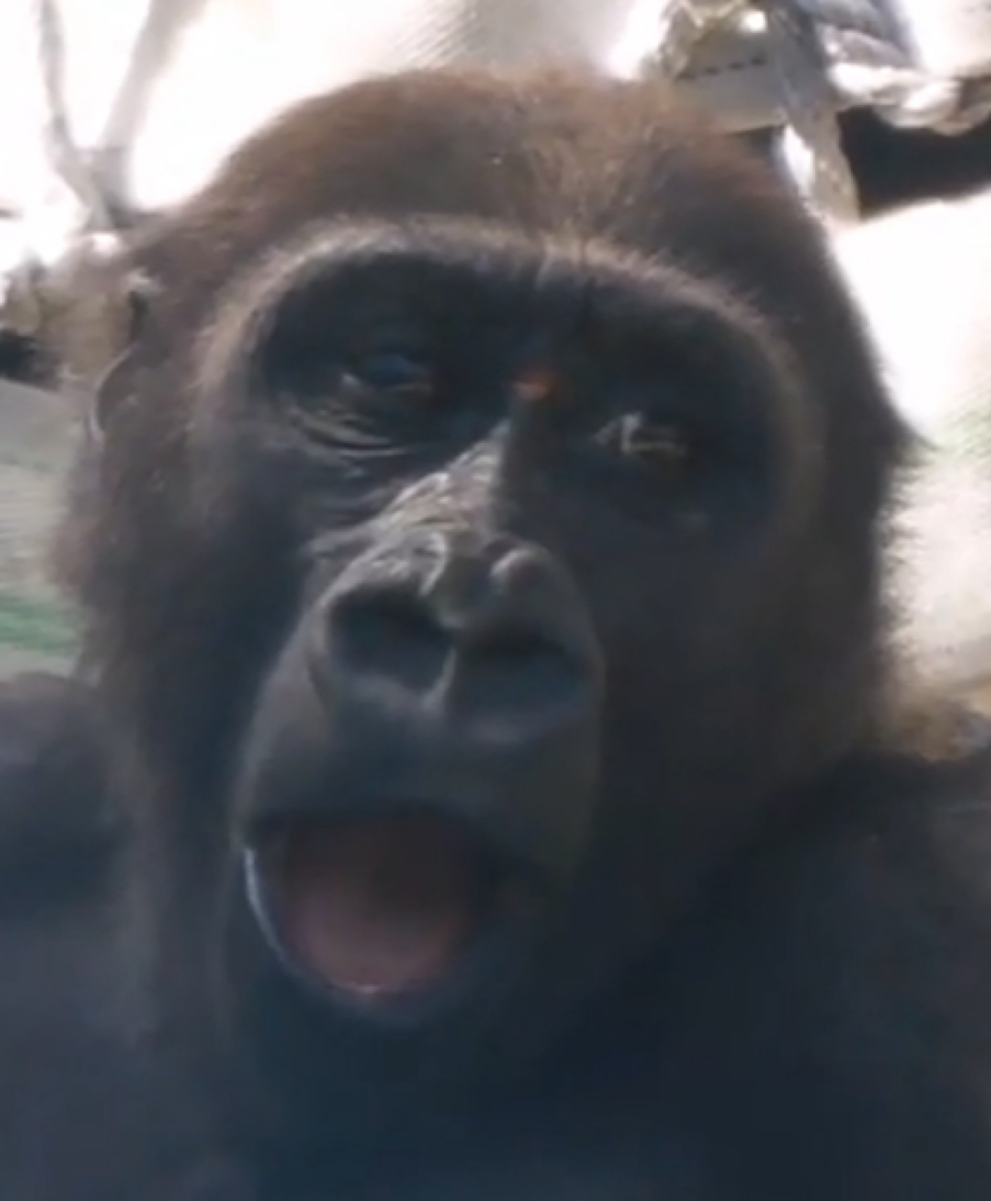
Example of mouth opening without AU12. Still frame from S27 Video in S1 Videos zip target.

**D. Subtle differences between AUs:** When AU26 or AU27 are acting, particularly with AU27, the lips might slide caudally due to the mouth aperture which may give the false impression of AU12 due to the lip corner angle (e.g., **Fig 17, S27a and S27b Video in S1 Videos zip target**). However, only code AU12 if a pulling movement is observed independently on the lip corner area or the characteristic concentric wrinkles are present. AU12 will deepen the lip corners when acting and make the lips appear longer.

*AU14—Dimpler*. In **humans**, this movement tightens the lip corners and pulls them slightly towards the ears. It is a very conspicuous movement as it creates dimples posteriorly to the lip corners. However, in **gorillas**, as in AU12, this movement is harder to detect. Other than the prognathism of the mouth making it difficult to detect the lip corners, gorillas do not produce dimples when tightening the lip corners. Hence, although the action is the same in both species (i.e., tightening the lips) by the same muscle (i.e., buccinator), some of the appearance changes differ drastically (e.g., **S28a-S30a and S28b-S30b Videos in S1 Videos zip target**).

**A. Proposed muscular basis:** Buccinator.

**B. Appearance changes**:

1. The lip corners are tightened and slightly pulled upwards towards the subnasal furrow and nose.
2. The area around the lip corners bulges and raises slightly.
3. The subnasal furrow deepens.
4. The upper lip may become narrower laterally.

**C. Minimum criteria**: The lip corner is tightened and the upper lip appears to compress against the lower lip in the corner area, in a squeezing movement of the lip corner.

**Fig 18:**
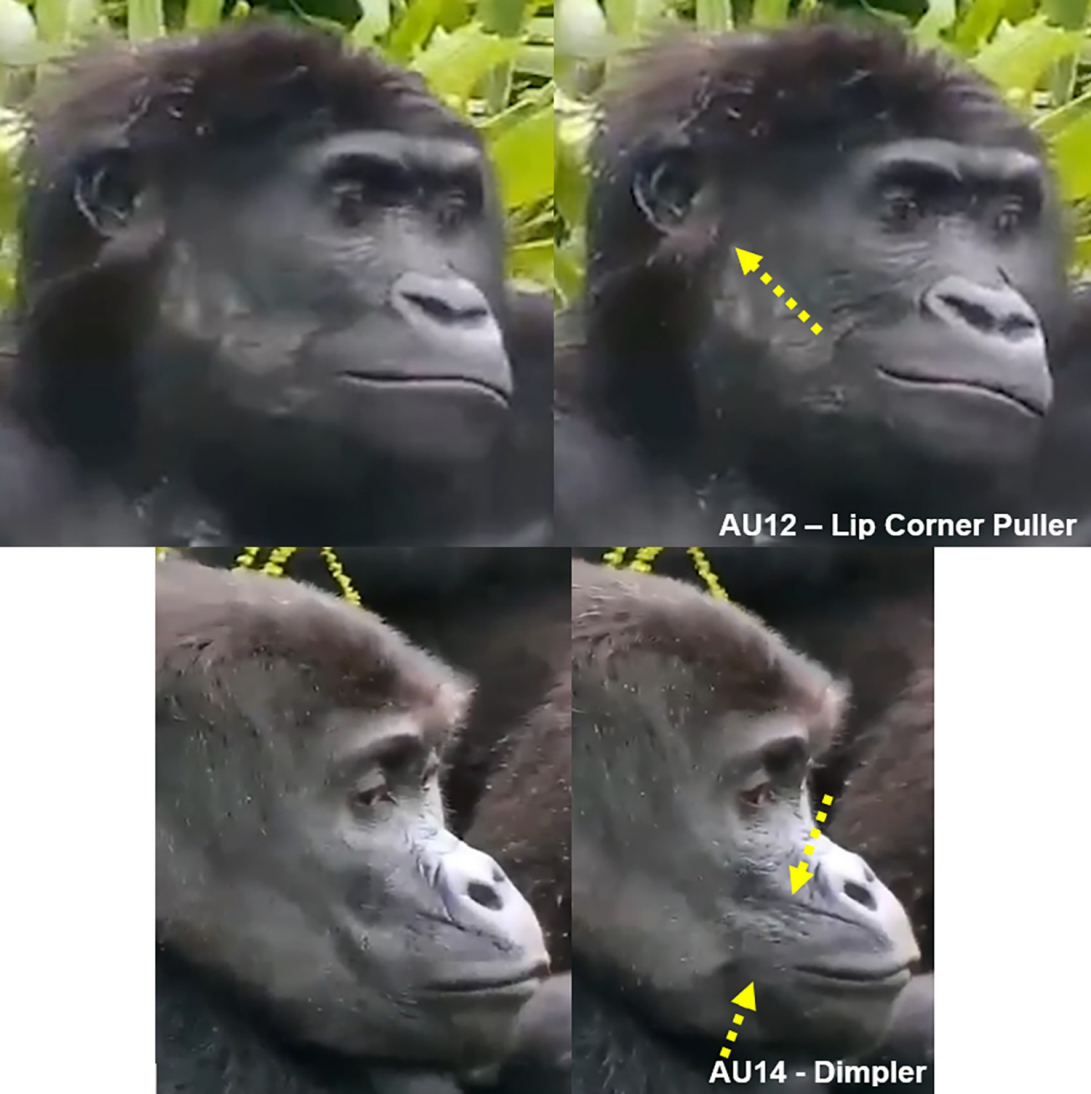
Comparison of AU12 –Lip Corner Puller vs AU14 –Dimpler. Top and bottom left images: Neutral mouth area; Top right: AU12 –Lip Corner Puller, identified by the concentric wrinkles behind the mouth corner; yellow arrow indicates direction of lip corner movement towards the ears. Bottom right: AU14 –Dimpler, identified by the slight bulging of the lip corner area and additional wrinkles due to lip corner area being compressed; yellow arrows indicate direction of compression of the lips in the lip corner area. Notice that in AU12 there is no tightening or squeezing of the lip corner, and in AU14 there is no concentric wrinkles formation behind the lip corner. Still frames from **S24 (top) and S29 (bottom) Videos in S1 Videos zip target**.

**D. Subtle differences between AUs:** This AU can only be produced when the lips are not parted, at least in the lateral areas, but the lips may still be slightly parted in the medial region. AU14 differs from AU12 in the direction of the lip corner movement: whilst in AU12 the lip corners move posteriorly towards the ears, in AU14 the lip corners move superiorly towards the nose. In AU12 there is no bulging of the lip corners like in AU14; conversely, in AU14 there are no concentric wrinkles or bunching up of the skin next to the lip corners. Another difference between these two AUs is that in AU12 the lip corner is pulled back, like a string is pulling this area, while in AU14 the lip corner area is squeezed, like there is a pinching movement on this area (e.g., **Fig 18**).

**Fig 19:**
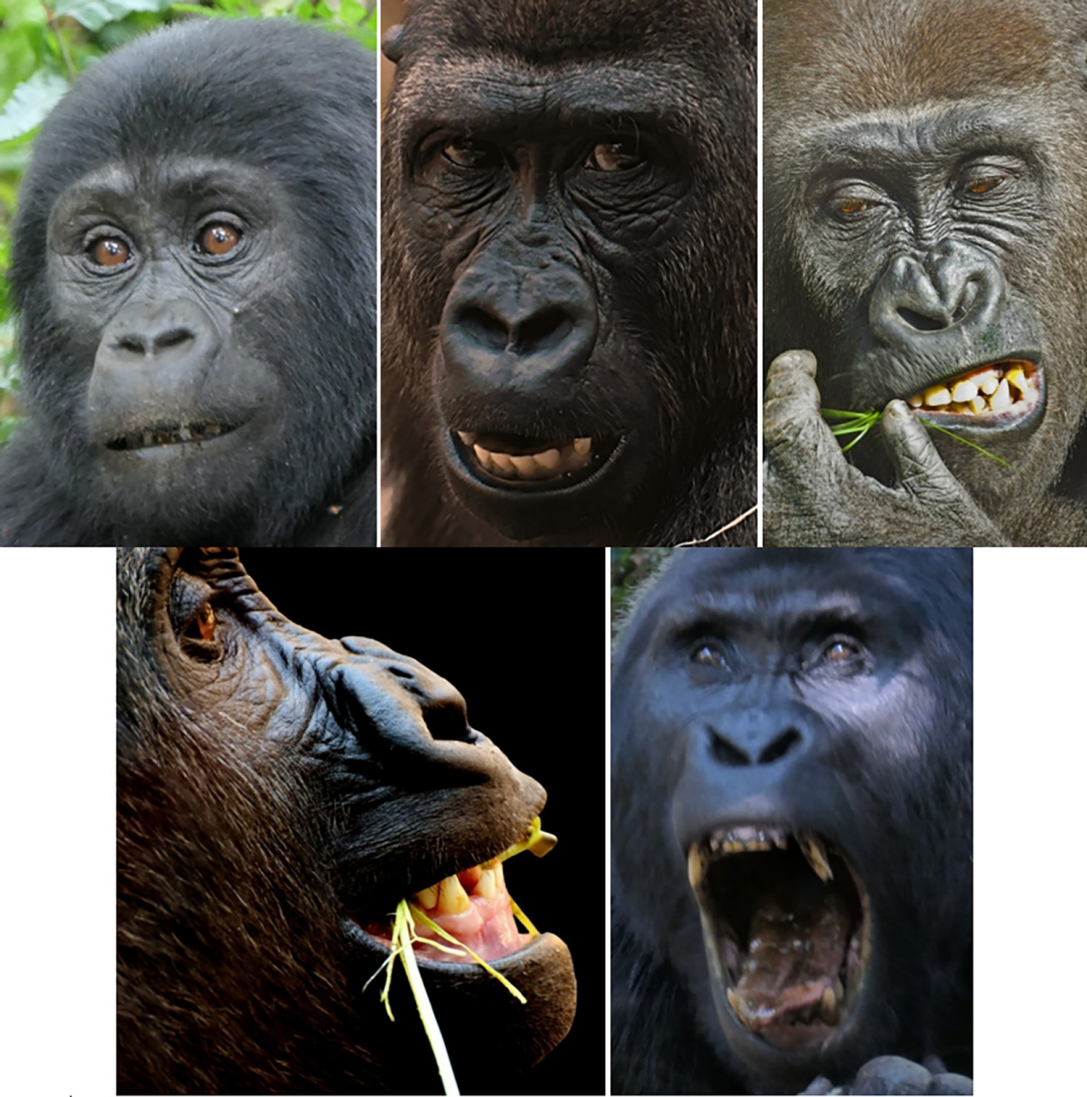
AU16—Lower Lip Depressor. Other AUs present. Pictures by MR, Alexas_Fotos, GerMay users from Pixabay.com, and RC.

*AU16—Lower Lip Depressor*. The Lower Lip Depressor—AU16—in **humans** and **gorillas** is produced by the same muscle, and it presents similar appearance changes (e.g., **Fig 19, S27a, S27b, S31a-S34a, and S31b-S34b Videos in S1 Videos zip target**). Like in humans, in gorillas this movement only occurs after parting of the lips (AU25).

**A. Proposed muscular basis:** Depressor labii inferioris.

**B. Appearance changes**:

1. The lower lip is pulled downwards, sliding over the mental region and parting the lips (hence AU16+AU25 is usually coded).
2. It may expose the lower teeth (e.g., **S31a and S31b Video in S1 Videos zip target**).
3. The mental region decreases in apparent size.
4. The inner part of the lower lip may become more visible.
5. The skin of the mental region may flatten/stretch.
6. In low intensity movements, the cues may be more apparent in the medial region (e.g., **S32a and S32b Video in S1 Videos zip target**).
7. It can be observed as a unilateral movement on one side of the mouth (AU16L or AU16R, e.g., **Fig 19**), or a more medial movement (e.g., **S32a and S32b Video in S1 Videos zip target**), or the whole length of the lip slides down.

**C. Minimum criteria**: The lower lip is pulled downwards either in the medial portion, the lateral portions or along the whole lip.

**D. Subtle differences between AUs:** When AU16 is acting, the lower lip must be pulled downwards, sliding along the chin. This movement may protrude the lip slightly, but do not code AU16 if the lower lip is just hanging loose; In this case, code AU160—Lower Lip Relax instead (see below for AU160 description). In addition, if the lower lip is presenting a flattened or funnelled shape while protruding forward, AU22—Lip Funneler (see below for AU22 description) can be coded instead (if there is no sliding downwards of the lip) or in addition to AU16 (if there is sliding downwards of the lower lip and protrusion in a flattened or funnel shape, e.g., **S33a and S33b Video in S1 Videos zip target**).

**Fig 20:**
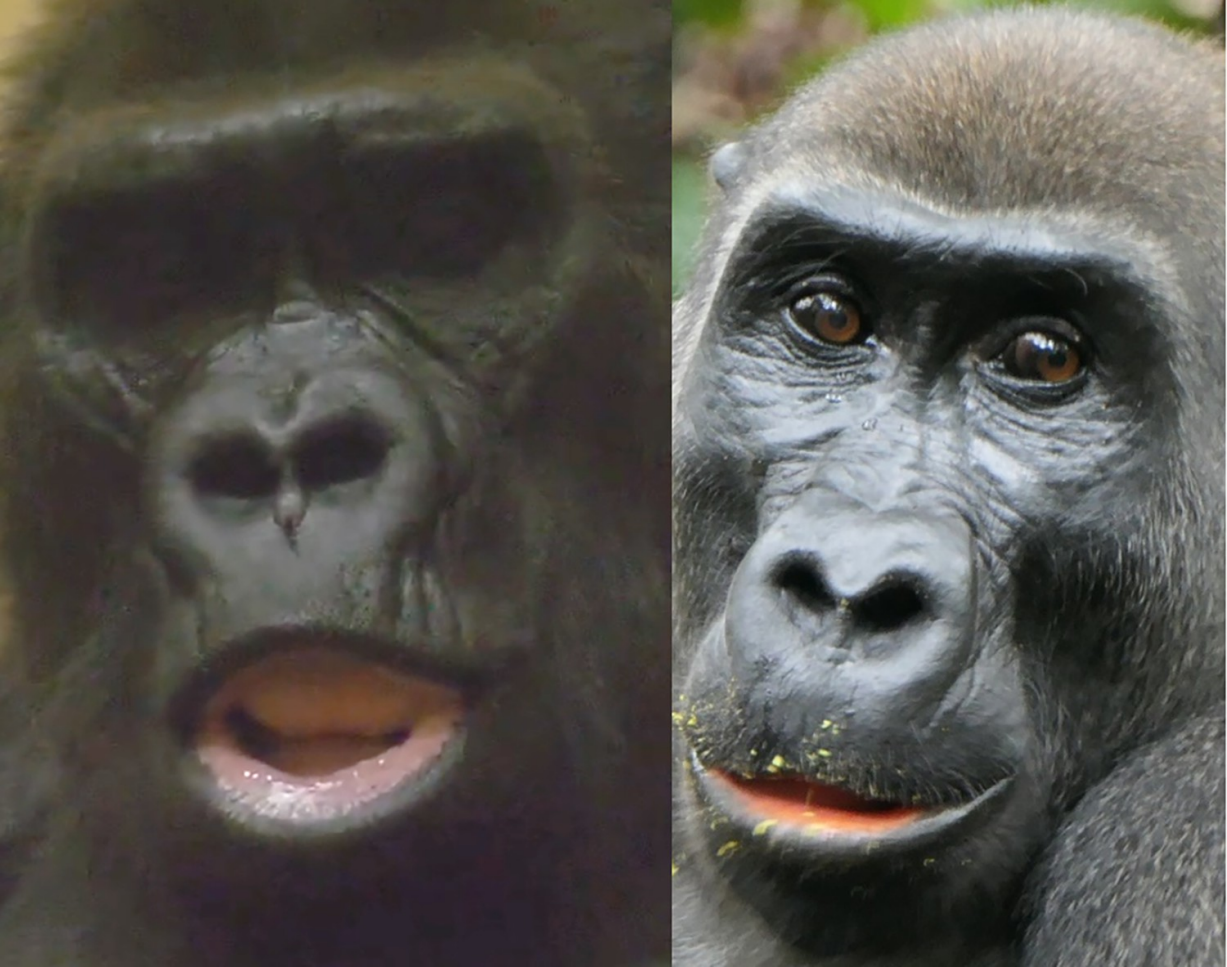
AU160—Lower Lip Relax. Other AUs present. Picture on the left is a still frame from YouTube.com videos and on the right by MR.

*AU160—Lower Lip Relax*. The Lower Lip Relax—AU160 describes the strong relaxation of the lower lip to the point it falls forward and hangs loose. This AU is not observed in **humans**, due to the small size and low flexibility of the human lower lip. It was however described for chimpanzees [5] and orangutans [11], since these species have a very large, thick, and highly mobile lower lip. In **gorillas,** AU160 is also observed, and it presents similar appearance changes to the other apes (e.g., **Fig 20**, **S35a, S35b, S36a, and S36b Videos in S1 Videos zip target**).

**A. Proposed muscular basis:** Relaxation of the lower lip and the lower portion of the orbicularis oris. This AU is thus not produced by the contraction of any muscle.

**B. Appearance changes**:

1. The lips are parted (AU25) and the lower lip is relaxed, falling forward and hanging loose (e.g., **S35a and S35b Video in S1 Videos zip target**).
2. It may expose the inner lip, mouth, gums and teeth.
3. The lower lip shape is modified and appears to be more concave in frontal view and with almost a pointy angle medially. In some cases, the lip may even fold outwards (as seen in last frames of **S36a and S36b Video in S1 Videos zip target**).
4. The lower lip also appears thicker when relaxed.
5. In side view, the lower lip appears to protrude forwards. This is also sometimes visible if the recording angle is from above the face plane, as the lower lip can be seen protruding more forward than the upper lip.
6. The mental region appears smaller.
7. The upper lip may accompany the mouth area relaxation and appear more extended than in a neutral face (e.g., **Fig 20**).
8. If the individual is moving or moving the head, the lip will dangle as a result. However, this is not due to any muscle contraction, but instead relaxation.

**C. Minimum criteria**: The lower lip is relaxed, falls forward to some degree and hangs loose.

**D. Subtle differences between AUs:** The main difference between AU16 and AU160 is that when AU16 is produced, the lower lip is pulled downwards sliding along the chin; This movement may protrude the lip slightly, but the lower lip is not loosely hanging from the mouth. If AU160—Lower Lip Relax is present, the lip falls forward and is loose. Usually, AU16 is a quicker movement than AU160, and AU160 tends to be displayed for longer periods than AU16. In AU16, the lip shape is also rounder and smoother, and thinner, whilst AU160 makes the lip appear pointier medially and appears thicker.

AU160 may also be confused with AU22, since in both AUs there is lip protrusion. However, in AU22 there is always a flattened or funnelled shape while protruding forward, and the lip is not loose. In still frames, AU22B might be hard to distinguish from AU160, if relaxation/contraction cues are not at a high intensity.

Some individuals may present some elongation of both their lips in a neutral face (e.g., **S14a and S14b, S50a, and S50b Videos in S1 Videos zip target**), which presents different appearance changes from AU22 and AU160. It is unclear if this is just an extreme relaxation of the whole mouth or if it is due to individual differences in the mouth of these particular individuals or due to age-related changes. Nonetheless, it should not be coded as AU160, unless the lip is hanging loose as described above.

**Fig 21:**
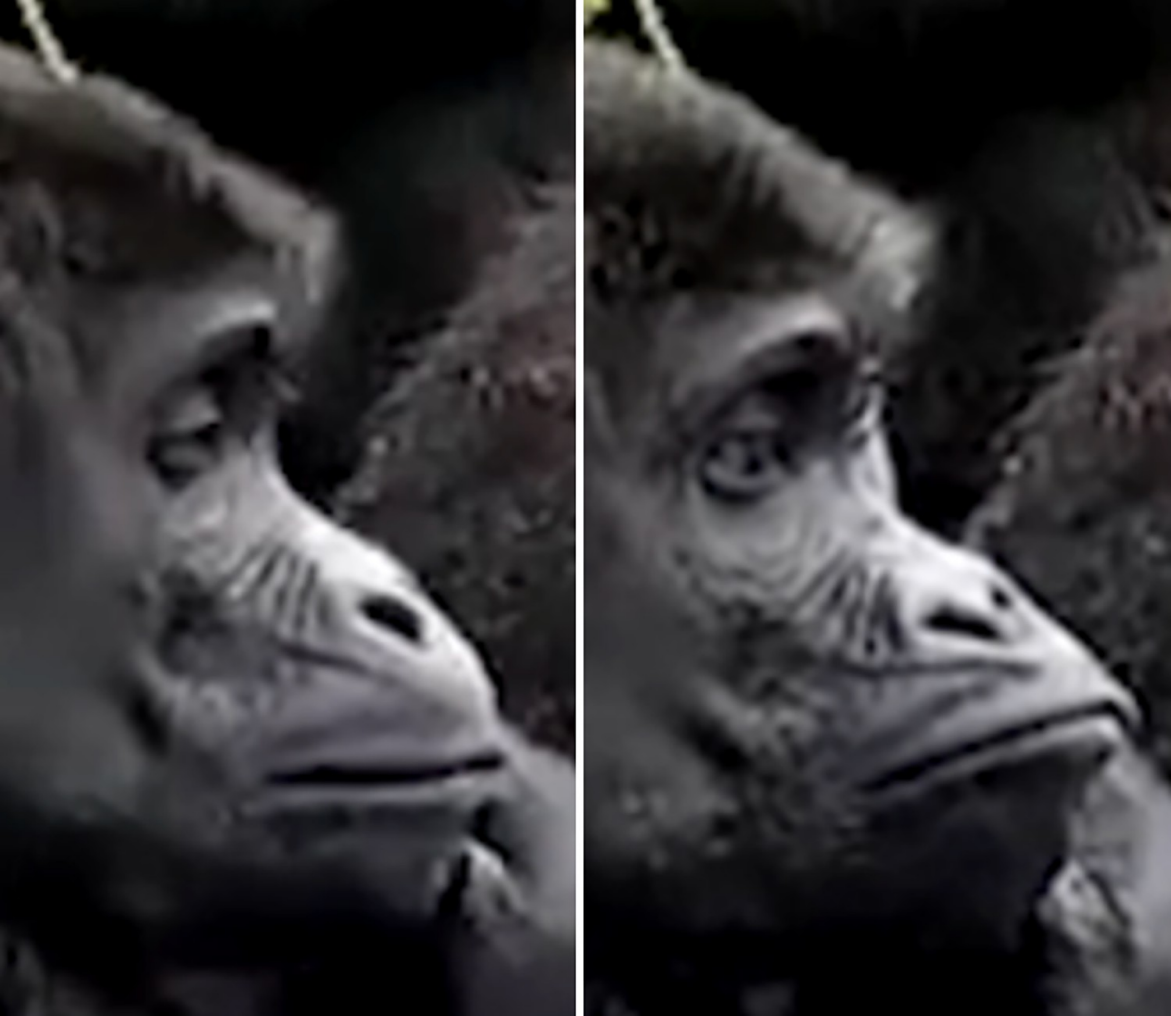
Left: Neutral mouth. Right: AU17—Chin Raiser. Still frames from **S38 Video in S1 Videos zip target**.

*AU17—Chin Raiser*. In **humans**, AU17—Chin Raiser is produced by the mentalis muscle that pushes the chin and lower lip upwards. **Gorillas** have the same muscle, but do not have an anatomical "chin" due to lack of the skull bony protuberance (chin boss) and a fat deposit on the mental region. The anatomical chin is a feature exclusive to the human face. Nonetheless, in **gorillas**, AU17 can still be coded with slightly different appearance changes in comparison to humans (e.g., **Fig 21, S37a-S42a and S37b-S42b Videos in S1 Videos zip target**).

**A. Proposed muscular basis:** Mentalis.

**B. Appearance changes**:

1. The lower lip is pushed upwards.
2. The mental region increases in size and may be projected forward (e.g., **S37a and S37b Video in S1 Videos zip target**).
3. The lower lip may increase in size and/or bulge.
4. If the mouth is closed, the lower lip may push the upper lip upwards (e.g., **S38a and S38b Video in S1 Videos zip target**) or cover the upper lip.
5. If the mouth is opened (AU25/AU26/AU27), the lower lip is extended upwards covering more of the mouth aperture.
6. The mouth corners may appear to be slightly pulled downwards and the medial area of the mouth appears pushed upwards.
7. It can be observed as a unilateral movement on one side of the mouth (AU17L or AU17R, e.g., **S39a and S39b Videos in S1 Videos zip target**), or a more medial movement, or the whole length of the lower lip may be pushed upwards.

**C. Minimum criteria**: The lower lip and/or mental region are pushed upwards, and/or the mental region may protrude.

**D. Subtle differences between AUs:** AU17 is mutually exclusive to AU16 as they are movements in opposing directions. However, AU17 might be confused with AU16 returning to neutral and vice-versa. Therefore, identifying the neutral position of the lower lip in a particular individual is important to distinguish these two movements and the release of the opposing movement.

Low intensity AU17 might be hard to code from still images as individuals have slightly variation in their mouth shape or they may relax their mouth area giving the false appearance of AU17 acting if the lips protrude forward. Hence, AU17 should only be coded when upwards movement of the lower lip/mental region is detected or if there is a neutral frame for comparison and no apparent relaxation of the mouth area. For AU17 to be coded, movement on the mental region is not needed, it is only needed to see movement on the lower lip, going upwards.

**Fig 22:**
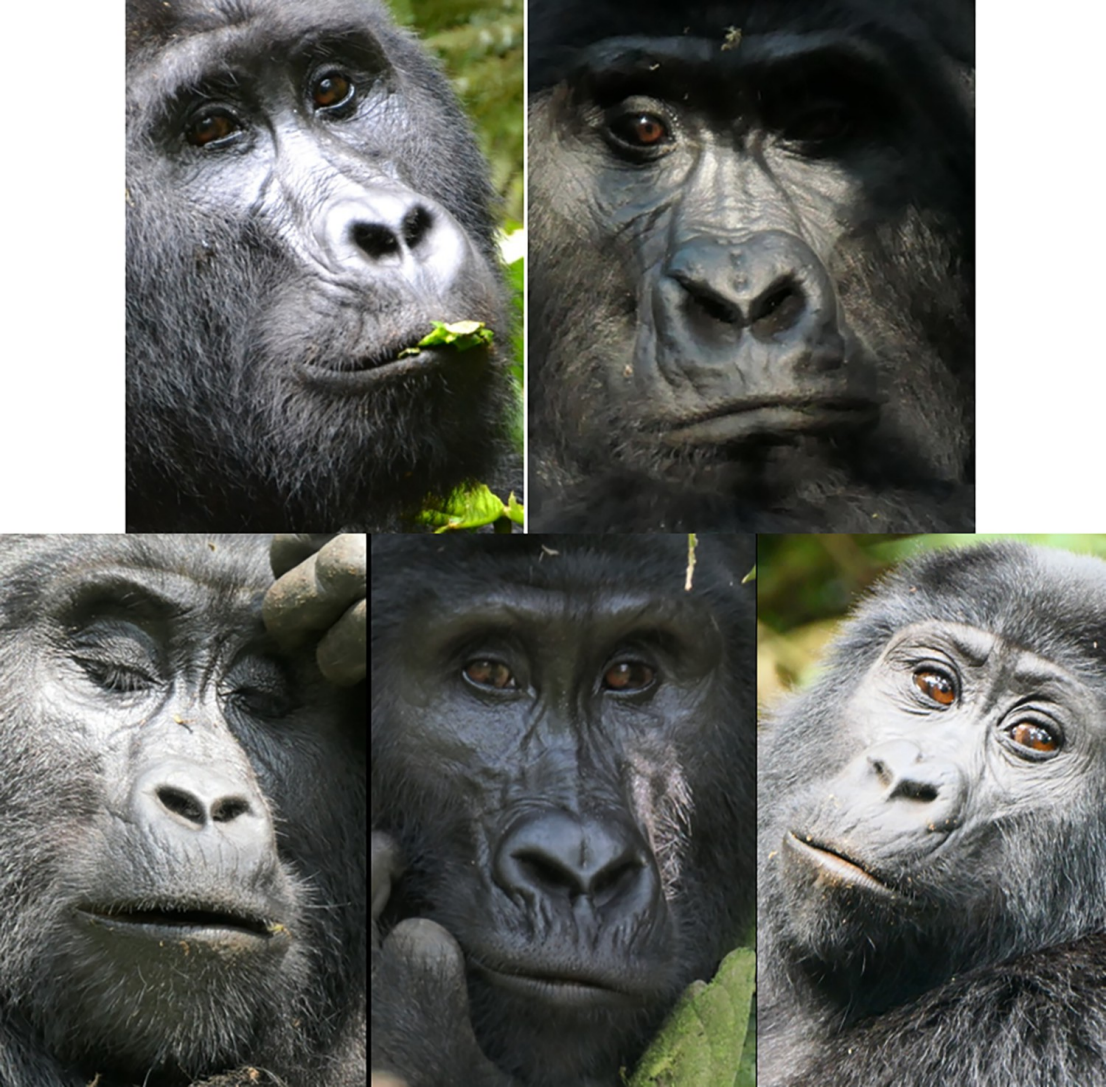
Examples of AU18—Lip Pucker. The lip corners are pushed medially and wrinkling of the upper lip is observed. Top left: AU18 with AU12 false appearance change of lip corners slightly pointing upwards. Top right: shows lip corners visible in frontal view due to these being brought forward during AU18. Bottom right: Low intensity AU18 showing lips brought slightly forward. Bottom centre: slightly asymmetrical AU18 (more intense on the left side of the face). Other AUs present. All pictures by RC except bottom right picture by MR.

*AU18—Lip Pucker*. In **humans**, AU18—Lip Pucker is produced by the orbicularis oris and incisivii labii muscles that bring the lip corners towards each other medially, puckering them. These muscles also exist in **gorillas**, but the characteristic puckering action with the wrinkling of the lips observed in humans appears slightly different in gorillas. In **gorillas**, AU18 can be produced with or without wrinkling, where the lip corners are drawn medially, but no puckering is observed (e.g., **Fig 22, S43a-S47a and S43b-S47b Videos in S1 Videos zip target**).

**A. Proposed muscular basis:** Orbicularis oris and incisivii labii.

**B. Appearance changes**:

1. The lip corners move towards the mouth midline or any section of the lips is pushed towards the mouth midline.
2. Some medial bulging of the lips may be observed.
3. Additional wrinkles are formed or the existent wrinkles become more conspicuous, particularly on the upper lip (e.g., **S43a and S43b Video in S1 Videos zip target**).
4. The mouth area may appear to become narrower as it appears compressed medially (e.g., **S43a and S43b Video in S1 Videos zip target**). In frontal view, the lip corners become visible (e.g., **Fig 22**).
5. The lip corners may appear to be slightly pointing upwards due to the movement of the upper lip being squeezed towards the mouth midline (e.g., **Fig 22**).
6. If the mouth is open, fewer teeth may become visible. The sharp angle of the lip corners becomes rounder.
7. Skin and hair accompany the lip corner movement towards the medial area of the mouth.
8. It is often observed as a unilateral movement on one side of the mouth (AU18L or AU18R, e.g., **S44a and S44b Video in S1 Videos zip target**). It can be observed with an alternating movement from AU18L to AU18R, appearing as a swinging motion of the lip laterally. It can also occur only on one of the lips (top lip: AU18T or bottom lip: AU18B).
9. In low intensity movements, only the lip corners move slightly forward, without wrinkling or mouth shape change. Hence, in low intensity movements, AU18 may not be visible from a full frontal view if the lip corners are not visible.

**C. Minimum criteria**: The lip corners are pushed medially or the lips are drawn together medially.

**D. Subtle differences between AUs:** AU18 is mutually exclusive to AU12 as they are movements of the lip corners in opposing directions. However, AU18 might be confused with AU12 returning to neutral and vice-versa. Therefore, identifying the neutral position of the lip corners in a particular individual is important to distinguish these two movements and the release of the opposing movement. In addition, with AU18 the lip corners may appear to be slightly pointing upwards (e.g., **Fig 22**), which is a false appearance change for AU12. However, in AU18 the lip corners move forward, not towards the ears as it happens in AU12.

Caution is needed when coding AU18 in gorillas, as many individuals have permanent wrinkles particularly on the top lip, which may act as false appearance changes to this AU. Also here, having a frame of the neutral mouth for each individual is important to understand when the AU18 acted, or as a minimum criterion to code this movement, movement of the lip corners or the lip itself moving medially must be observed.

**Fig 23:**
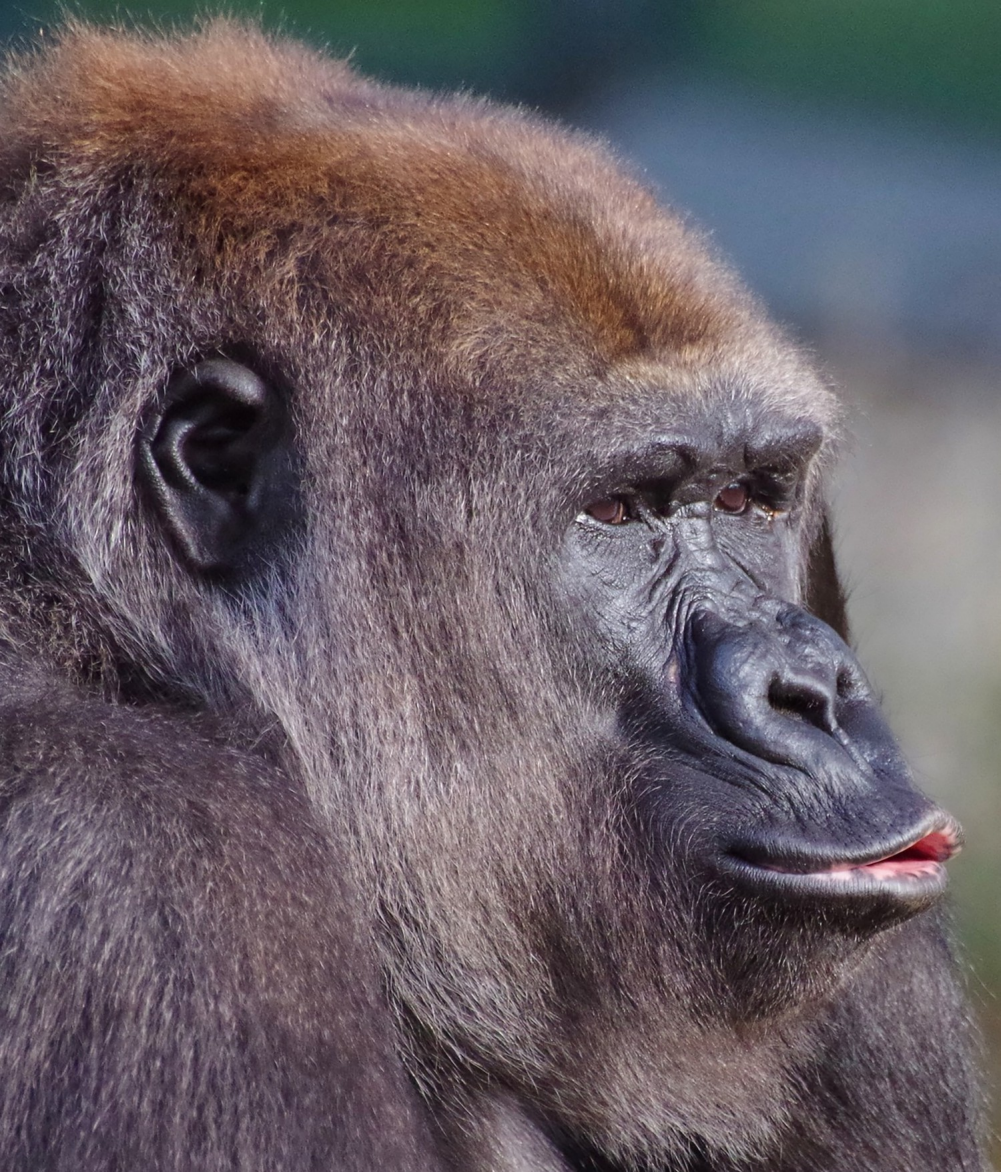
Example of AU22—Lip Funneler. Picture by 99564 user from Pixabay.com.

*AU22—Lip Funneler*. In **humans** and **gorillas**, AU22—Lip Funneler is produced by the orbicularis oris muscle to project the lips outwards in a typical funnelled or flattened shape. In **gorillas**, due to the larger size of the lips in comparison to humans, AU22 is a more conspicuous movement and can have more variation of how the lips are positioned during this movement. However, the appearance changes are similar between humans and gorillas (e.g., **Fig 23, S48a-S54a and S48b-S54b Videos in S1 Videos zip target**).

**A. Proposed muscular basis:** Orbicularis oris.

**B. Appearance changes**:

1. The lips are projected forward in a funnelled or flattened shape.
2. The lip corners are pushed forward, but not medially.
3. The edge of the lips may be turned outwards exposing the inner side of the lips.
4. The mouth area may appear to become narrower as it appears compressed medially. In frontal view, the lip corners become visible (e.g., **Fig 23**).
5. Wrinkling may be observed or the existent wrinkles become more conspicuous, on both lips. However, the opposite may also be observed, the lips become smoother, depending on the shape of the lips adopted and the individual’s natural wrinkling.
6. There is usually lip separation, in which case AU22+AU25 are coded together (e.g., **S48a, S48b, S50a, and S50b Videos in S1 Videos zip target**).
7. If there is no lip separation, it is likely AU17 has been added (e.g., **S49a, S49b, S50a, S50b, S52a, S52b, S53a, and S53b Videos in S1 Videos zip target**).
8. The soft nose shield accompanies the movement of the mouth and nostrils change shape and may become more elongated.
9. It can be observed as only on one of the lips (top lip: AU22T or bottom lip: AU22B).
10. It can be produced during vocalisations (AU22+AD50).

**C. Minimum criteria**: The lips are stretched forwards in a funnelled or flattened shape.

**D. Subtle differences between AUs:** This AU is usually easily detected due to the characteristic shape of the lips in most movements. It can be accompanied by different AUs, which may change slightly the expected appearance changes, but the characteristic shape must be present. Due to protrusion of the lips, in less intense movements it may confused with AU16, AU17, or AU18, but these do not present a funnel or flattened shape.

**Fig 24:**
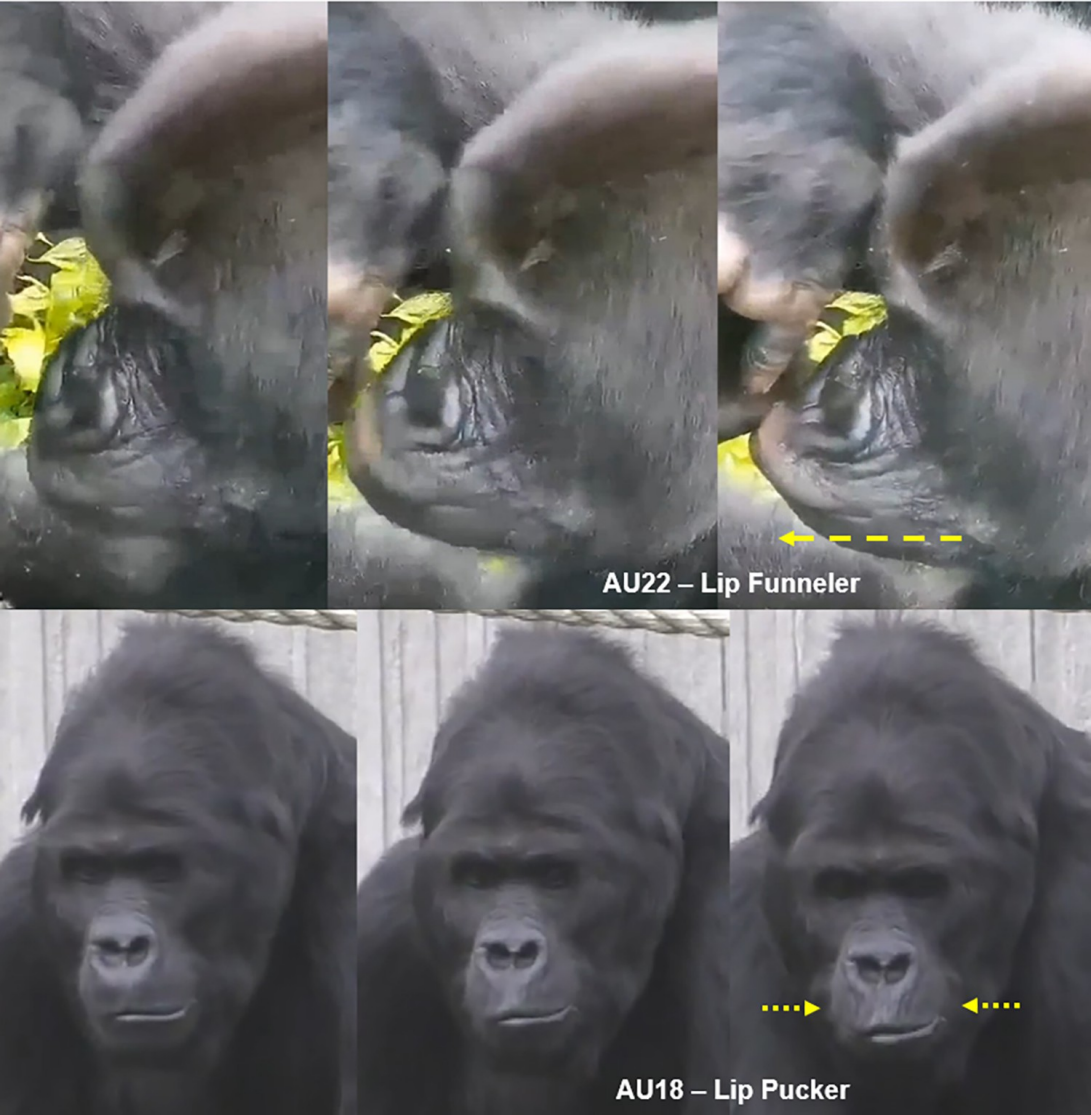
Comparison of AU18 –Lip Pucker vs AU22 –Lip Funneler. Top left: Neutral mouth area; Top centre: AU22 –Lip Funneler, in low intensity, identified by the upper lip flattening shape and smoother appearance; Top right: AU22 –Lip Funneler, in high intensity, identified by the upper lip flattening shape, smoother appearance, and slight pushing of the lip corners forward. Bottom left: Neutral mouth area; Bottom centre: AU18 –Lip Pucker, in low intensity, identified by the slight wrinkling on the top lip and medial protrusion; Bottom right: AU18 –Lip Pucker, in high intensity, identified by the marked wrinkling, protrusion of the lips medially, and lip corners pulled medially. Notice that in AU18 there is no flattening or funnelling of the lips, only medial protrusion in a slightly round shape. Yellow arrows indicate direction of movement, in which in AU22 the mouth area is pushed forward and in AU18 the mouth area is pulled medially. Still frames from **S51 and S47 Videos in S1 Videos zip target**.

To distinguish AU18 from AU22: in AU22 the lip corners do not move as much medially as they do in AU18, and usually only are pushed forward but not medially (e.g., **Fig 24**). In addition, AU18 presents wrinkles much more often than AU22, which sometimes can even act to smooth the lips. Nonetheless, these can co-occur, and it may be difficult to score both if there is not temporal separation of these two AUs (possible to score for e.g., in **S54a and S54b Videos in S1 Videos zip target**, due to AU18 being seen just before AU22).

To distinguish AU17 from AU22: AU17 on its own, will not protrude the lips to the point of flattening or protruding them, and with only AU17 no movement will be seen in the upper lip independently, other than being pushed by the lower lip up. If AU17 is added to AU22, the lower lip and mental region will be seen moving upwards, usually closing the lips or maintaining the lips together (e.g., **S49a, S49b, S51a-S53a, and S51b-S53b Videos in S1 Videos zip target**).

*AU122—Lower Lip Inner Curl and AU222—Lower Lip Extension*. In **humans** the lower lip is short, and other than the AU22, does not have a lot of flexibility to curl outwards. In **gorillas**, the lower lip is much larger than in humans and presents high flexibility and mobility, being often used to hold and manipulate objects or food. This differentiated lower lip morphology allows the production of movements that differ significantly from AU22, and hence here we describe two new lower lip movements for gorillas produced by the orbicularis oris: AU122—Lower Lip Inner Curl and AU222—Lower Lip Extension. In AU122, the lower lip is rolled onto itself inwards, whilst in AU222 the lower lip is extended and elongated outwards (e.g., **S55a-S57a and S55b-S57b Videos in S1 Videos zip target**).

**A. Proposed muscular basis:** Orbicularis oris.

**B. Appearance changes**:

1. In AU122 the lower lip is rolled onto itself inwards, whilst in AU222 the lower lip is extended outwards away from the teeth.
2. In AU122 the lip may be extended as well, but this is just so it can be rolled inwards.

**C. Minimum criteria**: In AU122 the lower lip has to present some curling inwards, and in AU222 the lip has to be extended away from the teeth.

**D. Subtle differences between AUs:** These two AUs—AU122 and AU222—differ from AU22 since the shape of the lower lip never becomes flattened or funnelled, but instead is curled inwards or extended away from the teeth. AU122 also differs from AU28 as the lip is not inserted into the mouth nor it covers the teeth, but it rolls itself inward outside the teeth/mouth.

**Fig 25:**
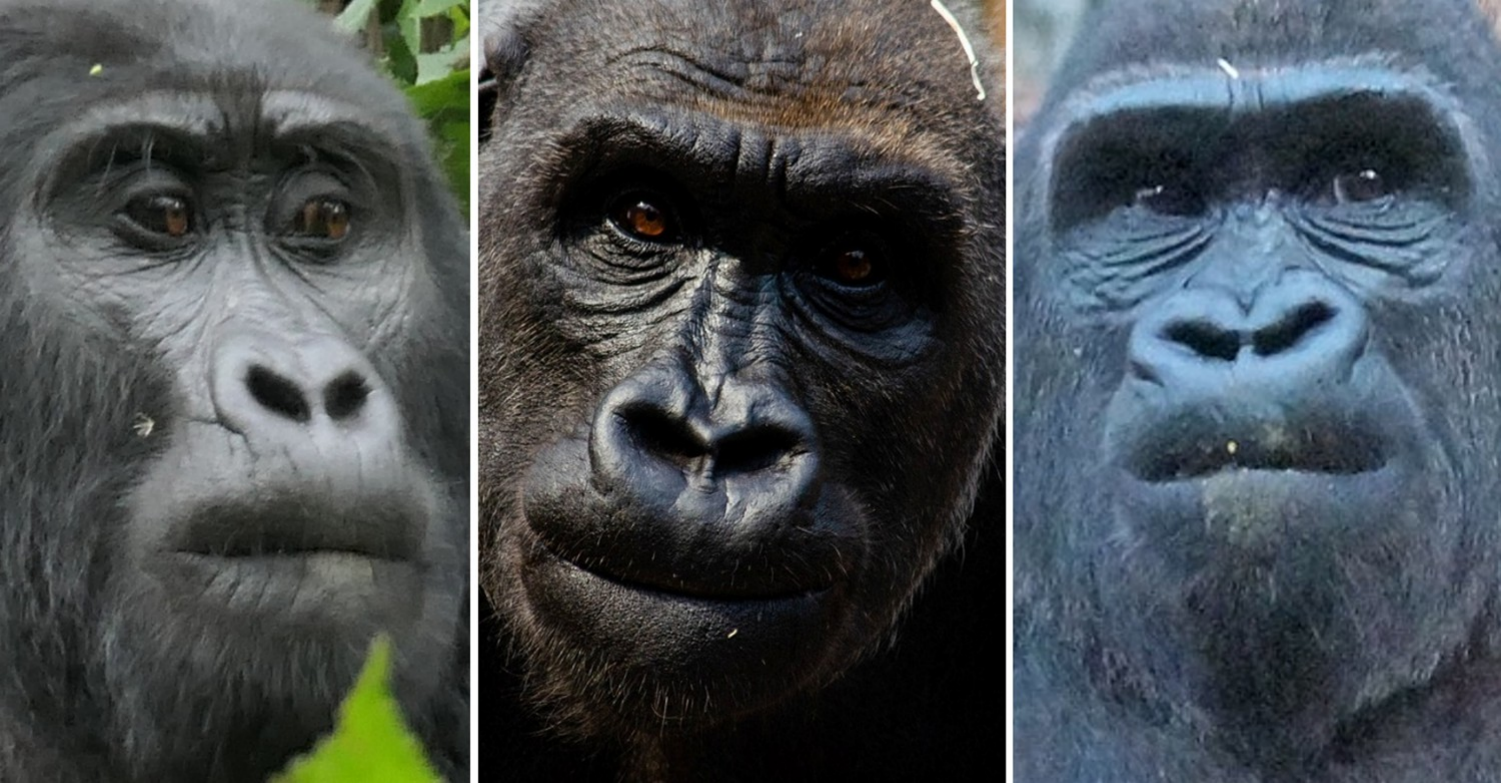
Examples of AU24—Lip Presser. Pictures by RC (left), and imranhussain1343431 user (centre) and Pixel-mixer user (right) from Pixabay.com.

**Fig 26:**
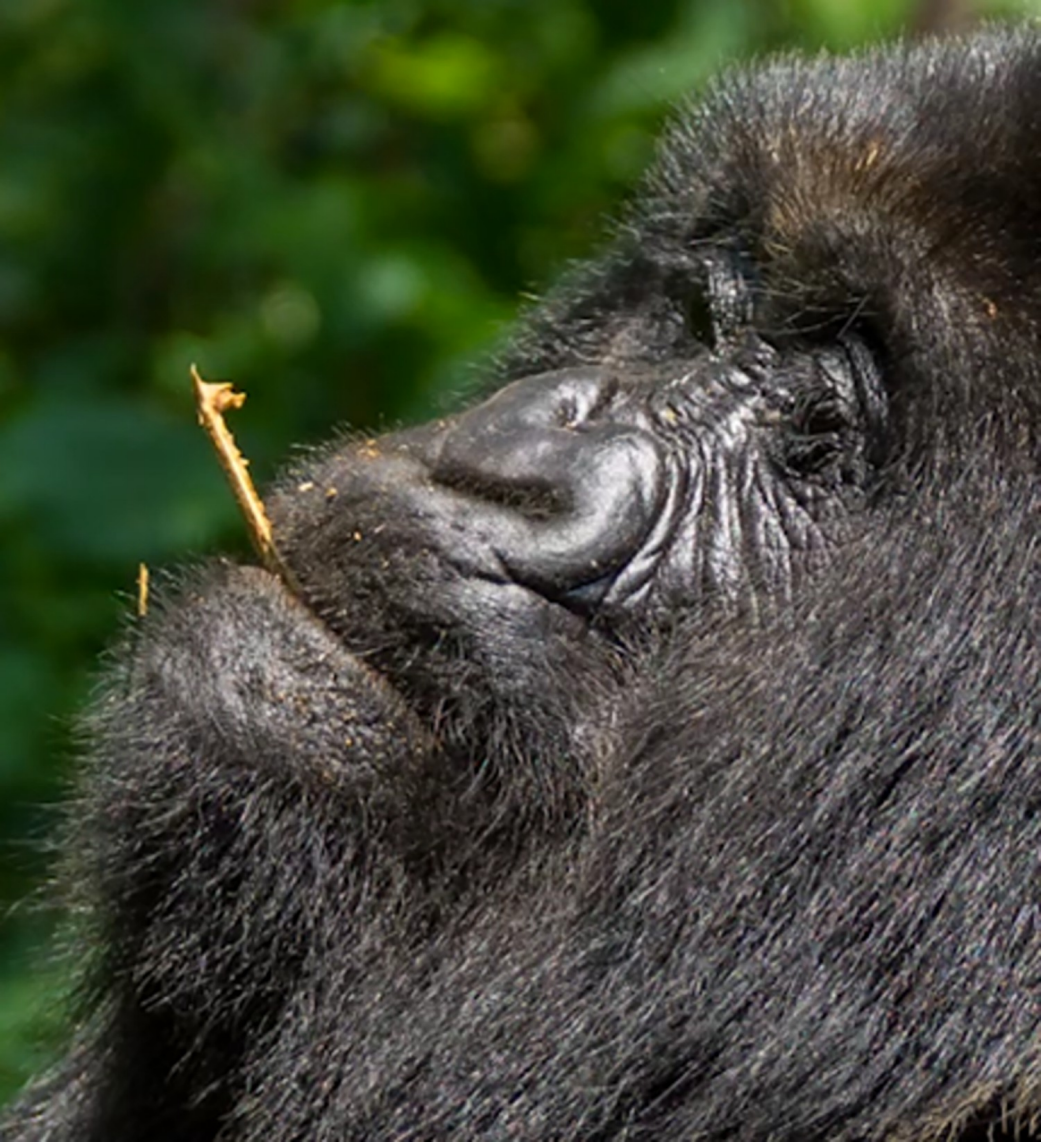
Example of AU24—Lip Presser in profile view whilst holding an object between the teeth/lips. Still frame from YouTube.com videos.

*AU24—Lip Presser*. In **humans** and **gorillas**, AU24—Lip Presser is produced by the orbicularis oris muscle, in which both lips are pressed against each other. In **gorillas**, due to the larger size of the lips in comparison to humans, AU24 is a more conspicuous movement (e.g., **Figs 25 and 26, S58a-S62a and S58b-S62b Videos in S1 Videos zip target**).

**A. Proposed muscular basis:** Orbicularis oris.

**B. Appearance changes**:

1. The upper and lower lips bulge whilst they are being pushed against each other.
2. The distance between the soft nose shield and the edge of the upper lip appears shorter.
3. The mouth appears wide and less vertically extended in frontal view.
4. Existent permanent wrinkling on the lips become less conspicuous and the lip appears smoother whilst bulging.
5. The edge of the lips may be turned inwards slightly.

**C. Minimum criteria**: The lips are pressed against each other and there is bulging on at least one of the lips.

**D. Subtle differences between AUs:** This AU has to be scored in both lips, and no AU25 can be present (AU24 and AU25 are thus mutually exclusive). However, it can be coded with AU26, in which case the bulging may be less conspicuous. AU24 was observed in gorillas whilst holding an object between the teeth/lips, in which case separation of the jaw (AU26) and lips (AU25) happens due to the object, and not necessarily muscle action. Hence, here AU25/AU26 are not coded.

In more intense AU24 movements, the lips appear to be sucked into the mouth; however, if lips enter the mouth beyond the teeth and an AU26 is seen, then AU28—Lips Suck, must be coded as well—see below for AU28 description. AU24 and AU28 may be confused, but in AU28 there is no bulging of the lips.

**Fig 27:**
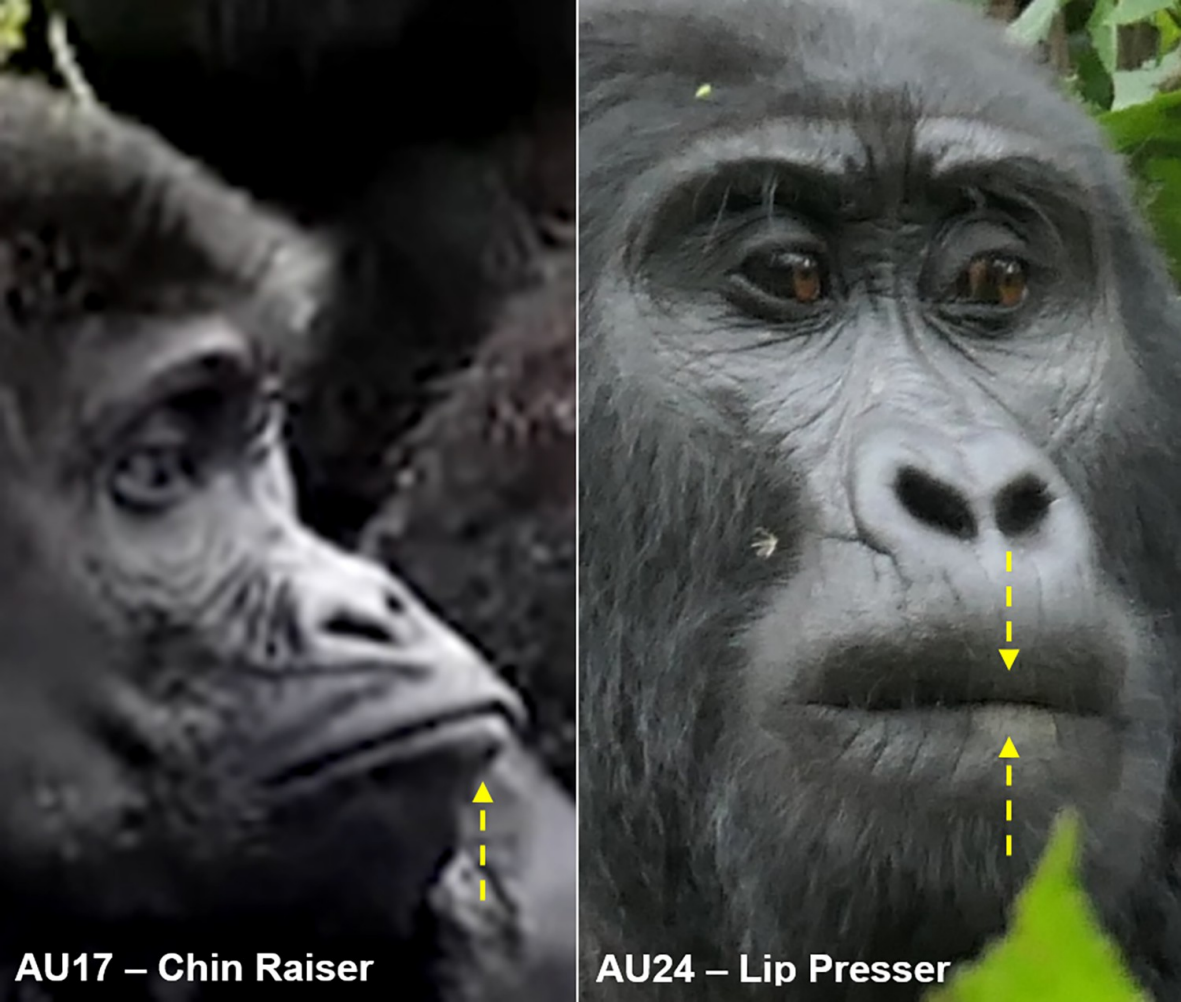
Comparison of AU17 –Chin Raiser (left) and AU24 –Lip Presser (right). In both movements the lower lip has an upward movement, and in both movement the mentalis is likely pushing up the lower lip. However, in AU24, this movement is counteracted by the upper lip pushing down the lower lip, with also contraction of the orbicularis oris to push both lips against each other, resulting in bulging of both lips. In AU17, the upper lip is neutral, so the lower lip and mental region are pushed upwards, without any involvement of the orbicularis oris or upper lip. Hence, if there is no temporal separation seen between these two movements, they are not coded simultaneously, as the resulting appearance changes are very different. Yellow arrows indicate direction of movement of the lips. Still frame from **S38 Video in S1 Videos zip target** (left) and by RC (right).

AU24 can also be confused with AU17, and AU17 can act together with AU24 (e.g., **S62a and S62b Videos in S1 Videos zip target**). However, AU17 can only be coded if there is a clear upwards movement of the lower lip that is not counteracted by the upper lip pushing downwards with equal strength (e.g., **Fig 27**).

AU24 was often observed with AU18 (e.g., **S60a and S60b Videos in S1 Videos zip target**). In this case, the permanent wrinkles do not disappear, or new wrinkles may appear on the lips, and the lip corners are brought more medially than just with AU24.

*AU25—Lips Part*. In **humans** and **gorillas**, AU25 codes the separation of the lips, i.e., it is coded whenever a space is observed anywhere between the upper and lower lip (**e.g., S63a and S63b Videos in S1 Videos zip target**). Unlike most AUs, AU25 can be caused by several different muscles and AUs. Hence, it is usually coded with the AUs that caused the lips to part (if there are enough appearance changes to identify the other AUs). For example, AU10 alone might cause AU25, in which case AU25+AU10 are coded.

**A. Proposed muscular basis:** A range of muscles attaching to the lips might produce this movement, including orbicularis oris, levator labii superioris, depressor labii inferioris, etc. Jaw opening also can produce AU25.

**B. Appearance changes**:

1. The lips are separated at any point and a space can be observed between the upper and lower lip.
2. The inner lips or teeth may become visible.

**C. Minimum criteria**: The lips part, and a space is observed between the lips.

*AU26—Jaw Drop*. In **humans** and **gorillas**, AU26—Jaw Drop describes a movement produced by the relaxation of jaw muscles (masseter and temporalis). These non-mimetic muscles open the jaw slightly by relaxing (e.g., **S63a, S63b, S64a, and S64b Videos in S1 Videos zip target**). At rest, the jaw muscles are contracting to keep the jaw closed and in a neutral position.

**A. Proposed muscular basis:** Masseter and temporalis muscles relaxation.

**B. Appearance changes**:

1. The lower jaw is lowered and teeth separation can be clearly seen or inferred.
2. This jaw lowering is a relaxation movement, of small amplitude, where no sign of tension or skin stretching around the mouth is visible.
3. If the lips part (if AU25 is also present), teeth might become visible. However, AU26 can occur without lip parting (e.g., **S65a and S65b Videos in S1 Videos zip target**). In this case, the mouth shape may change to be more extended vertically.

**C. Minimum criteria**: The lower jaw moves downwards in a small and relaxed movement.

**D. Subtle differences between AUs:** AU25 and AU26 are dorsoventral movements of the mouth, but AU25 refers only to the parting of the lips, while AU26 refers to the parting of the jaw. They can be coded independently, as it is possible that the lips are parted without opening the jaw, and conversely, the jaw might be slightly opened without the lips parting. In the case that both lips and jaw are parted, AU25+AU26 is coded. Code AU26 also when the jaw is parted due to an object keeping the jaw separated (e.g., food held between upper and lower teeth, tongue placed between upper and lower teeth). In still images, a weak AU26 might be difficult to code if there is no clear separation of the teeth.

**Fig 28:**
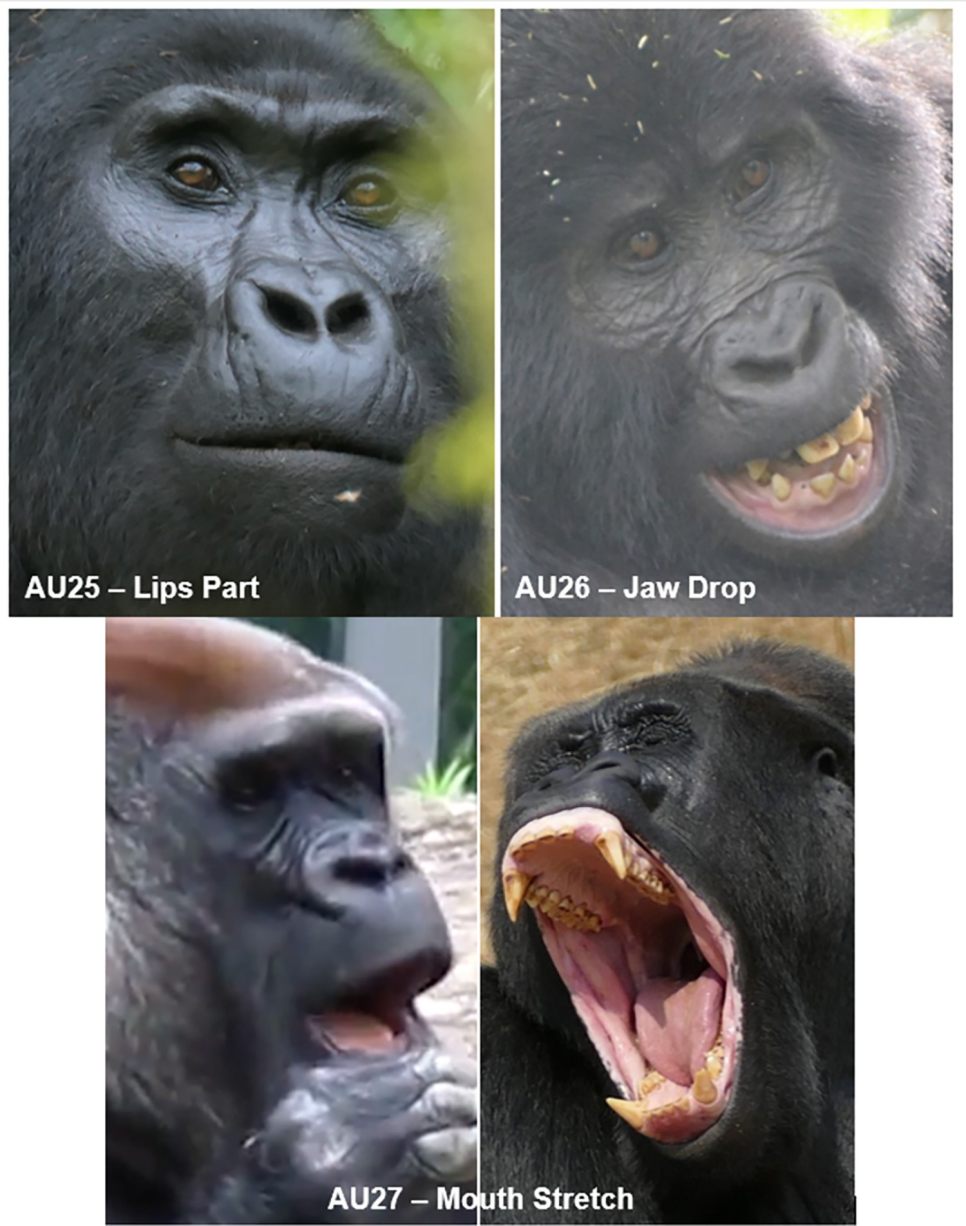
Comparison of AU25—Lips Part, AU26—Jaw Drop, and AU27 Mouth Stretch. Top left: AU25. Top right: AU26. Bottom left: AU27. Bottom right: Maximum intensity AU27. Other AUs present. Pictures by RC (top), still frame from **S66 Video in S1 Videos zip target** (bottom left), and by MrsBrown user from Pixabay.com.

*AU27—Mouth Stretch*. In **humans** and **gorillas**, AU27—Mouth Stretch, describes a movement produced by the pulling of the lower jaw downwards (by non-mimetic muscles), stretching the mouth wide open. Although AU27 describes a larger degree of mouth opening in relation to AU26, AU27 is mutually exclusive to AU26, as different muscles are involved. Due to the degree of mouth opening always separating the lips, it is coded as AU25+AU27 (e.g., **Fig 28**, **S66a and S66b Video in S1 Videos zip target**).

**A. Proposed muscular basis:** The non-mimetic muscles that open the jaw include the anterior belly of the digastric muscle, the mylohyoid muscle, and the inferior head of the lateral pterygoid muscle. These muscles are likely very well developed in gorillas producing their unusually large gape during gouging.

**B. Appearance changes**:

1. The mouth is opened wide by lowering the lower jaw and actively stretching it downwards.
2. The lips become stretched and flatten against the teeth.
3. The lower teeth, tongue and oral cavity are exposed. The upper teeth may also be exposed.
4. The global shape of the face becomes vertically extended.
5. In strong movements, the lips may retract as the opening of the mouth forces the lips to slide caudally.
6. AU27 must be coded together with AU25 as there is always lip separation due to the mouth opening.
7. Often combined or immediately followed by other AUs.

**C. Minimum criteria**: The mouth is stretched open, further than jaw relaxation (AU26), with clear signs of skin stretching.

**D. Subtle differences between AUs:** Although AU26 and AU27 describe visually different degrees of mouth opening, and are produced by different muscles and different muscle actions (relaxation and contraction, respectively), these AUs are mutually exclusive. A wide AU26 and a weak AU27 can be confused, especially in still images. Careful examination of the movement itself, noticing stretching and tension around the mouth and lips, helps in deciding if the jaw is being relaxed or actively pulled down. In a strong AU27, AU12 is also often present, stretching the mouth horizontally and further increasing tension and stretching around the mouth, nose and infraorbital region. AU10 and AU16 are also frequently observed together with AU27. On the other hand, when AU25/26/27 act, the upper and/or lower teeth may become visible and produce a false indicator as if AU10 or AU16 is acting. However, code AU10 or AU16 only if there is clear movement of each lip upwards or downwards, respectively, and/or different onsets/offsets are observed. AU26 and AU27 might also be alternated, where a temporal distinction might be useful to decide when to code AU26 as an independent movement, or not to code it as it is produced as a precursor to AU27. For example, if AU26 is held for a couple of seconds, and then AU27 follows, then both AU26 and AU27 should be coded. Alternatively, for each instance of mouth opening, only the highest degree of opening (i.e., the apex of the mouth opening) might be coded, which means that if an AU27 follows AU26 without mouth closure, only AU27 is coded regardless of AU26 duration.

**Fig 29:**
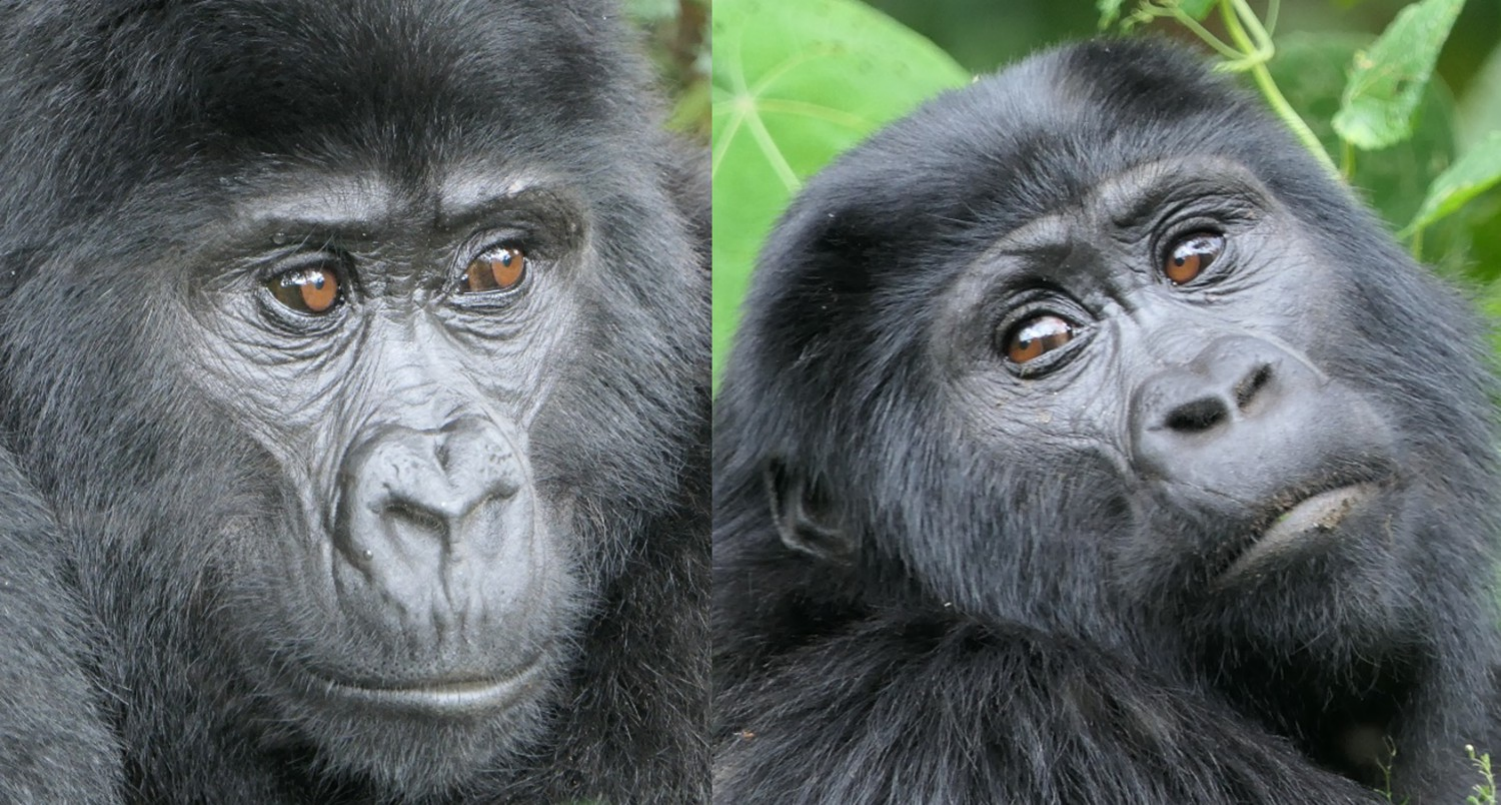
Left: Neutral mouth. Right: AU28 –Lips Suck. Note the lower lip is almost all inserted into the mouth. Other AUs present. Pictures by MR.

*AU28—Lips Suck*. In **humans** and **gorillas**, AU28—Lips Suck have similar appearance changes, with the main difference being the larger size and higher mobility of the lips in gorillas, which makes this movement more conspicuous. The morphology of the lips in gorillas also means that this movement can be produced without AU26, whilst in humans is almost always produced with AU26 so there is space to insert the lips inside the mouth beyond the teeth. In both species, the orbicularis oris muscle inserts the lips or parts of the lip(s) into the mouth or they are sucked into the mouth (e.g., **Fig 29**, **S67a, S67b, S68a, and S68b Videos in S1 Videos zip target**).

**A. Proposed muscular basis:** Orbicularis oris.

**B. Appearance changes**:

1. The lips are inserted or sucked into the mouth.
2. The lips are stretched and may be flattened against the teeth, covering the teeth.
3. It may make the lower lip appear thinner and the mental region may appear to become more salient (e.g., **S67a and S67b Video in S1 Videos zip target**).
4. AU26 can be coded if the jaw is seen moving downwards in order to insert the lip beyond the teeth.
5. It can be coded in only one lip (AU28T or AU28B, e.g., **Fig 14**, **S68a, and S68b Video in S1 Videos zip target**) and/or unilaterally (AU28R or AU28L).

**C. Minimum criteria**: The lips are sucked or inserted into the mouth.

**D. Subtle differences between AUs:** Gorillas, like other apes, have very flexible and extensible lips that, when relaxed, can be considerably far apart from the teeth. Therefore, AU28 can be seen with or without AU26, because without AU26, the lips are being sucked in and rolled inwards slightly towards the teeth and not into the mouth beyond the teeth.

When seen in profile, it may appear as the lips are bulging slightly due to the medial part of the mouth (lips) being pulled inside. This may be a false indicator for the AU24, but in order to code AU24, pressing movement of the lips against each other must be visible.

AU28 can also be confused with AD32—Bite (see **SI3** for description of AD32 in S1 File), since it can be hard to tell whether the lip(s) have been sucked in or are being held in the mouth by a biting action. If the teeth can be seen biting the lip, AD32 is scored instead of AU28. If the teeth cannot be seen, and the entire lip (top or bottom) has disappeared and it could be due to sucking or biting, AU28 should be scored.

*AU38—Nostril Dilator*, *AU39—Nostril Compressor*, *AU138—Nose Shield Expander*, *AU139—Nose Shield Flattener*, *AU238—Nose Downwards*. In **humans** the nostrils are widened with AU38—Nostril Dilator, or constricted with AU39—Nostril Compressor, by the action of the nasalis muscle. The depressor nasi septi muscle is also activated during AU39. These are usually subtle movements, but important in human communication and emotion [84]. **Gorillas** present a much larger and more developed nasal region than humans, and a more connected nose to the upper lip. These differences in facial morphology affect the appearance changes to code AUs. Gorillas also seem to have a more diverse range of movements around the nose shield and nostrils. In gorillas, we observed three new movements not found in humans or other apes: AU138—Nose Shield Expander, AU139—Nose Shield Flattener, and AU238—Nose Downwards. In AU138 the nose shield is pushed forward expanding the size of the nose shield to protrude forward (e.g., **S69a and S69b Video in S1 Videos zip target**). AU139 also produces movement more globally in the nose shield and less in the nostril wings, in which the whole nose shield is slightly flattened against the face. AU139 has been observed as a single movement (e.g., **S70a and S70b Video in S1 Videos zip target**), but also as a repeated sequence of movements (e.g., **S71a and S71b Video in S1 Videos zip target**), resembling sniffing behaviour in some species (e.g., dogs: [13]). It is unclear if sniffing is occurring during the sequence of AU139. Finally, we observed a slightly different movement of the whole nose shield, AU238, in which the whole nose shield is moved downwards towards the upper lip, but without any independent movement of the upper lip (e.g., **S72a and S72b Video in S1 Videos zip target**). Like AU38 (e.g., **S73a and S73b Video in S1 Videos zip target**) and AU39 (e.g., **S74a and S74b Video in S1 Videos zip target**), all these movements can be subtle movements or very quick movements, but are more conspicuous since these tend to involve the whole nose shield rather than just the nostrils, hence movement can be detected in a larger facial feature.

**A. Proposed muscular basis:** Nasalis and depressor nasi septi.

**B. Appearance changes**:

1. In AU38, the nostril increases in size (e.g., **S73a and S73b Video in S1 Videos zip target**), whilst in AU39 decreases in size (e.g., **S74a and S74b Video in S1 Videos zip target**). In AU138 the whole soft nose shield appears more salient (e.g., **S69a and S69b Video in S1 Videos zip target**), and in AU139 the whole soft nose shield appears more flattened (e.g., **S70a, S70b, S71a, and S71b Videos in S1 Videos zip target**). Finally, AU238 pulls the soft nose shield down (e.g., **S72a and S72b Video in S1 Videos zip target**).
2. In AU38/AU39 the nostrils usually change shape or at least the nostril wings will present some movement, whilst in AU138/AU139/AU238 no nostril movement is needed and instead the soft nose shield presents movement.
3. The skin next to the nose and on the nose shield as a whole might move, enlarging, contracting, projecting forward, or flattening, accompanying each of the respective movements.

**C. Minimum criteria**: The nostrils are widened in AU38 and are narrowed in AU39. The soft nose shield is expanded in AU138 and is flattened in AU139. In AU238, a downwards movement of the soft nose shield has to be seen.

**D. Subtle differences between AUs:** Caution regarding appearance change 3 is needed, as movement of the skin around the nose might be due to other AUs pulling the skin globally in the mouth region (e.g., AU9, AU10, AU16). Hence, AU38/AU39/AU138/AU139/AU238 are coded only if the minimum criterion for each AU is present, i.e., if the movement is clearly originating in the nostrils or soft nose shield. Additionally, these nose AUs may be difficult to detect depending on the angle and distance of the individual from the camera. Changes in head position might appear to change the nostril shape. Therefore, if there are head movements that can change the observed nostril shape or the soft nose shield shape, the nose AUs should be coded only when movement is detected in the nostrils or the soft nose shield.

In particular, an intense AU9 may move the whole soft nose shield upwards and change the shape of the nostrils due to the LLSAN muscle acting. However, this movement is not due to the nasalis or nose muscles directly; Hence AU38/AU39/AU138/AU139/AU238 should not be coded, if there is not a clear temporal separation between AU9 and the nose movements. AU9 may be confused with AU38/AU39/AU138/AU139, whilst the release of AU9 may be confused with AU238.

In addition, AU38/AU39 might be hard to code in a side view if the nostrils are not fully visible, but AU138/AU139/AU238 are usually visible from both a frontal and side view.

While AU38 is mutually exclusive to AU39, and AU138 is mutually exclusive to AU139, as these are movements in opposite directions, some of these movements may be combined. For example, we observed a combination of AU38 and AU138, in which the soft nose shield was projected forward and the nostrils were expanding simultaneously (e.g., **S75a and S75b Video in S1 Videos zip target**). In this case, both AUs can be coded. Equally, AU39+AU139 can be coded, as well as AU38+AU238 or AU39+AU238.

Finally, it might be hard to determine the neutral position of the nose and the neutral size of the nostrils for each gorilla, hence, for AU38/AU39/AU138/AU139 movements that have opposite mutually exclusive movements that may happen in sequence, caution should be applied to not code the movement of returning to neutral. Hence, in each coding event, the first movement detected should be coded as an AU, for example, if the nostrils are seen expanding, code AU38 –Nostril Dilator, but the following movement of the nostrils reducing in size should not be coded also as AU39 –Nostril Contraction, as this should be considered a return to neutral.

## Discussion and conclusions

In total, 28 AUs and 14 ADs have been identified in gorillas, which indicates a comparable number of actions found in humans (32 AUs), but higher than other NHP such as chimpanzees (15 AUs), orangutans (17 AUs), rhesus macaques (15 AUs), and gibbons (20 AUs).

Importantly, this potential for facial mobility in gorillas appears to be much higher than previously thought, taking into account their socio-ecological characteristics [48, 49] and behaviour [47, 48]. Indeed, the GorillaFACS confirms previous predictions of gorillas as the NHP with highest facial expressivity potential, due to several factors thought to be related with higher number of facial movements, namely: 1) having plain coloured faces [85], 2) being a very large species with a large face [48], 3) forming large cohesive groups with high social complexity [86], 4) having the same facial number of muscles as humans (except for the Risorius muscle which seems variable [64, 65]), 5) being more terrestrial than arboreal [86].

In addition, and as detailed in the introduction, the gorilla is a highly social species with a wide range of social behaviours (e.g., [87]). They make use of complex visual displays [54, 88], including at least some facial expressions [47, 51, 55]. However, even when accounting for gestures [50] and vocalisations [89, 90], gorilla communication and expressivity have been much less studied than other NHP. To date, there has been no detailed study of facial expressions use and function in gorillas. Likely due to the myth of gorillas as “stone faced” and perhaps due to the lack of a scientific tool to measure facial movements in gorillas, there is a gap in this area. The current methodological work, which aimed to identify and classify the full potential of movement in the gorilla face, presents a new tool to allow its anatomical, objective, and standardised identification and measurement. It also opens up the possibility of multimodal studies, for example simultaneously measuring vocalisations, gestures, and facial expressions.

Being the last ape to have a dedicated FACS, the GorillaFACS now allows a complete cross-species comparison within the apes and with other NHP (e.g., with Old World monkeys [6] and New World monkeys: [12]). One potential application of GorillaFACS is to facilitate research into the evolution of communication and emotions in humans and other animals, since the gorilla is an interesting model due to this taxa phylogenetic position [49].

In more applied settings, such as when working with individuals in captivity (e.g., zoos) or in the wild when there is close proximity between humans and gorillas (e.g., ecotourism sites [57]), GorillaFACS may be a useful tool to help improve gorilla welfare and human-gorilla interactions. As such, GorillaFACS is an important tool, not only to aid in evaluating the welfare and monitor species-specific behaviour of gorillas in captive settings (e.g., zoos, sanctuaries, research facilities), but also to better understand gorilla communication in order to better protect these endangered species.

It is important to note though that the GorillaFACS as such other FACS, only presents the potential for movement, and not the actual use of the facial movements, such as frequency of use or duration of each movement. Humans and gorillas have the largest number of facial muscles within primates, i.e., 24 muscles (excluding ear and mastication muscles) [68, 91] as well as the largest number of AUs; however, this does not necessarily translate as humans and gorillas equally having the highest facial mobility, as other muscle features, such as their subdivisions, arrangements of fibres, topology, biochemistry, and microanatomical mechanical properties, or osteological and external features (e.g., colour) may also impact facial mobility [91]. The actual use of these muscles for communication and emotional expression may not necessarily follow predictions from the anatomical potential. Hence, the need for GorillaFACS to be applied in studies of facial behaviour in gorillas, as well as comparative studies with other species to assess the true facial mobility of this taxa and make further conclusions.

Although we analysed a large sample of gorilla populations, including three of the four subspecies, we do not know to what extent there might be variation in the facial muscular plan between these subspecies, as all dissections published till date are from the *G*. *g*. *gorilla* subspecies. Given the minimal variation in other closely related species of primates within a genus, we expect there to be little difference in facial morphology between gorilla subspecies. For example, in the *Macaca* genus, which is a very diverse taxa in terms of facial morphology and most socio-ecological and behavioural factors, the facial muscular plan was found to have minimal differences along the despotic-tolerant spectrum [92]. Importantly, these differences were not found to affect the successful validation of the MaqFACS, initially developed for rhesus macaques, for Barbary [7], crested [9], and Japanese macaques [8]. Furthermore, it is difficult to find available specimens from the other subspecies for dissection due to dwindling population numbers and/or location and protection status of the biological specimens [31]. Future work may explore facial muscle differences, not only among the subspecies, but also within each subspecies, to investigate individual potential variations, if specimens become available. The gorilla subspecies presents diversity in habitat, diet, behaviour, social structure, and morphology [93], so it is possible that some of these factors may influence not only the use of the facial muscles, but also the muscles itself, for example, in terms of presence, attachment sites, size, and shape, or at a more micro-anatomical level. The subspecies living in larger groups and in less arboreal environments (i.e., with more potential for sociality and less visually occluded environments) such as *G*. *g*. *beringei* [31, 93], may make more use of facial expressions and thus have evolved more robust facial musculatures. However, as we mention above, the facial muscular plan and extrinsic factors are not always strictly associated, therefore future research will need to address these questions.

Furthermore, as we presented in the current work, there seem to be slight variations regarding morphological facial features which may affect the detection of AUs, but we did not systematically measure differences between subspecies or populations. This is however now possible to do by applying the GorillaFACS tool to measure differences between subspecies, contexts, and individuals.

Despite the many advantages of using FACS (e.g., objectivity, standardisation, high detail, allowing quantification of AUs and cross-species comparisons, see also [19]), its application is very time-consuming, both in terms of requiring coders to spend time studying FACS and obtaining certification for each species of interest, but also during the actual video-coding of facial movements, in which the frame-by-frame analysis of just a few minutes of video can take many hours. Furthermore, due to the quick and subtle nature of facial expressions, FACS coding can only be used with video recordings of individuals, not in real time. Even with FACS currently being the gold standard to measure facial movements, both of these factors, i.e., long time and asynchronous video analysis, should be considered when applying this tool. The development of the GorillaFACS also opens up the possibility of automation of this tool, as it has been done already for the human FACS [94] and the MaqFACS [95].

The GorillaFACS revealed a higher number of facial movements than in other NHP, suggesting higher potential for mobility in gorillas. However, it is also possible that when identifying AUs, different conditions may have been in place artificially inflating the number of AUs, such as for example greater experience of the coders with FACS, larger and more diverse video samples, and/or better quality cameras, etc. There were three new AUs described in gorillas in the nasal region (AU138, AU139, and AU238), and two new AUs related to the lips (AU122 and AU222). These five new movements are the main differences when compared with the human FACS and other NHP FACS, which may mean the increased mobility may be mostly in the nasolabial region, instead of a more generalised facial mobility. Therefore, future studies of cross-species FACS comparisons will be important to better understand the real facial mobility of each species.

Another potential limitation is that the environmental conditions of this taxa in the wild, i.e., dark/shadowy forests, combined with the dark facial skin of gorillas may make it harder to collect good quality video, where all facial movements are easily visible. This may be the reason why gorillas were often thought as being not very facially expressive, as the human vision is not optimal for these conditions (i.e., dark forests and dark faces). However, a detailed analysis with a tool such as the GorillaFACS may help detecting facial movements that are usually hard to see, even in poor lighting conditions, as observers that are GorillaFACS trained will be more attuned to facial movements of these species.

## Supporting information

S1 Videos zip segment Each Videos ZIP Segment file is a segment of a split ZIP. To access the videos in the split ZIP, download all Videos ZIP Segment files that are part of the split ZIP to a single folder (including the Videos ZIP Target file that has the full “.zip” extension), rename all of the downloaded files to have the same root filename (e.g., rename “pone.0308790.s001.z01” to “Videos.z01”, rename “pone.0308790.s002.z02” to “Videos.z02”, rename “pone.0308790.s003.z03” to “Videos.z03”, etc.), then open the file with the “.zip” extension (e.g., “Videos.zip”), navigate the folders within the ZIP and select a video to open. Alternatively, after downloading all ZIP and ZXX files into a folder, unzip all files by opening the .ZIP file with a compressing software such as WinRAR, WinZip, or 7-Zip. This will give access to the 177 GorillaFACS video examples organized in their respective folders for each AU and AD.
https://doi.org/10.1371/journal.pone.0308790.s001
(Z01)

S2 Videos zip segment https://doi.org/10.1371/journal.pone.0308790.s002
(Z02)

S3 Videos zip segment https://doi.org/10.1371/journal.pone.0308790.s003
(Z03)

S4 Videos zip segment https://doi.org/10.1371/journal.pone.0308790.s004
(Z04)

S5 Videos zip segment https://doi.org/10.1371/journal.pone.0308790.s005
(Z05)

S6 Videos zip segment https://doi.org/10.1371/journal.pone.0308790.s006
(Z06)

S7 Videos zip segment https://doi.org/10.1371/journal.pone.0308790.s007
(Z07)

S8 Videos zip segment https://doi.org/10.1371/journal.pone.0308790.s008
(Z08)

S9 Videos zip segment https://doi.org/10.1371/journal.pone.0308790.s009
(Z09)

S10 Videos zip segment https://doi.org/10.1371/journal.pone.0308790.s010
(Z10)

S11 Videos zip segment https://doi.org/10.1371/journal.pone.0308790.s011
(Z11)

S12 Videos zip segment https://doi.org/10.1371/journal.pone.0308790.s012
(Z12)

S13 Videos zip segment https://doi.org/10.1371/journal.pone.0308790.s013
(Z13)

S14 Videos zip segment https://doi.org/10.1371/journal.pone.0308790.s014
(Z14)

S15 Videos zip segment https://doi.org/10.1371/journal.pone.0308790.s015
(Z15)

S16 Videos zip segment https://doi.org/10.1371/journal.pone.0308790.s016
(Z16)

S17 Videos zip segment https://doi.org/10.1371/journal.pone.0308790.s017
(Z17)

S18 Videos zip segment https://doi.org/10.1371/journal.pone.0308790.s018
(Z18)

S19 Videos zip segment https://doi.org/10.1371/journal.pone.0308790.s019
(Z19)

S20 Videos zip segment https://doi.org/10.1371/journal.pone.0308790.s020
(Z20)

S21 Videos zip segment https://doi.org/10.1371/journal.pone.0308790.s021
(Z21)

S22 Videos zip segment https://doi.org/10.1371/journal.pone.0308790.s022
(Z22)

S23 Videos zip segment https://doi.org/10.1371/journal.pone.0308790.s023
(Z23)

S24 Videos zip segment https://doi.org/10.1371/journal.pone.0308790.s024
(Z24)

S25 Videos zip segment https://doi.org/10.1371/journal.pone.0308790.s025
(Z25)

S26 Videos zip segment https://doi.org/10.1371/journal.pone.0308790.s026
(Z26)

S27 Videos zip segment https://doi.org/10.1371/journal.pone.0308790.s027
(Z27)

S28 Videos zip segment https://doi.org/10.1371/journal.pone.0308790.s028
(Z28)

S29 Videos zip segment https://doi.org/10.1371/journal.pone.0308790.s029
(Z29)

S30 Videos zip segment https://doi.org/10.1371/journal.pone.0308790.s030
(Z30)

S1 Videos zip target Once you have downloaded all of the ZIP Segments and this ZIP Target file to the same folder and renamed all files to have the same root name, open this target file (with “.zip” extension) to navigate the folders and view the videos. Alternatively, after downloading all ZIP and ZXX files into a folder, unzip all files by opening the .ZIP file with a compressing software such as WinRAR, WinZip, or 7-Zip. This will give access to the 177 GorillaFACS video examples organized in their respective folders for each AU and AD. Video captions for each video can be found in the S2 File.
https://doi.org/10.1371/journal.pone.0308790.s031
(ZIP)

S1 File Supporting Information containing the following sections: SI1: List of Zoos; SI2: Glossary; SI3: Action Descriptors; SI4: Head and Eye Action Descriptors, SI5: Gross behaviour codes; SI6: Visibility codes; SI7: Ear movements not observed in gorillas.
https://doi.org/10.1371/journal.pone.0308790.s032
(DOCX)

S2 File Captions for the Supporting Information Videos.
https://doi.org/10.1371/journal.pone.0308790.s033
(DOCX)
