# Supplementary material for: GorillaFACS: The Facial Action Coding System for the Gorilla spp
Source: PLoS One. 2025 Jan 28;20(1):e0308790. doi: 10.1371/journal.pone.0308790 (PMC11774405; doi:10.1371/journal.pone.0308790)
Supplement: S1 File — Supporting Information containing the following sections: SI1: List of Zoos; SI2: Glossary; SI3: Action Descriptors; SI4: Head and Eye Action Descriptors, SI5: Gross behaviour codes; SI6: Visibility codes; SI7: Ear movements not observed in gorillas. https://doi.org/10.1371/journal.pone.0308790.s032 (DOCX) [file pone.0308790.s032.docx]

**Supporting Information for:**

**GorillaFACS: The Facial Action Coding System for the *Gorilla spp*.**

Catia Correia-Caeiro^1,2*^, Raquel Costa^3,4,5^, Misato Hayashi^3^, Anne Burrows^6,7^, Jordan Pater^6^, Takako Miyabe-Nishiwaki^8^, Jack L. Richardson^9^, Martha M. Robbins^10^, Bridget Waller^11^, Katja Liebal^1,2^

^1^Human Biology & Primate Cognition Department, Institute of Biology, Leipzig University, Leipzig, Germany

^2^Comparative Cultural Psychology, Max Planck Institute for Evolutionary Anthropology, Leipzig, Germany

^3^Research Department, Japan Monkey Center, Inuyama, Japan

^4^Primate Cognition Research Group, Lisbon

^5^Mulheres pela Primatologia, Brazil

^6^Department of Physical Therapy, Duquesne University, Pittsburgh, USA

^7^Department of Anthropology, University of Pittsburgh, Pittsburgh, USA

^8^Center for the Evolutionary Origins of Human Behavior (EHuB), Kyoto University, Inuyama, Japan

^9^Center for the Advanced Study of Human Paleobiology, Department of Anthropology, The George Washington University, Washington, DC, USA

^10^Department of Primate Behavior and Evolution, Max Planck Institute for Evolutionary Anthropology, Leipzig, Germany

^11^Department of Psychology, Department of Psychology, Nottingham Trent University, Nottingham, UK

***Corresponding author**

E-mail: catia_caeiro@hotmail.com (CCC)

**ORCID**: https://orcid.org/0000-0002-2819-6039

**SI1: List of Zoos:**

**Table S1:** List of zoos in which recording of gorillas were made and analysed from public sources (licensed as CC or with video owner permission) such as YouTube.com, Pixabay.com, etc.

| **Zoo Name** | **Country** |
| --- | --- |
| Antwerp Zoo | Belgium |
| Apenheul Zoo | Netherlands |
| ARTIS Amsterdam Royal Zoo | Netherlands |
| Barcelona Zoo | Spain |
| Basel Zoo | Switzerland |
| Beekse Bergen Safaripark | Netherlands |
| Berlin Zoo | Germany |
| Bristol Zoo | UK |
| Burgers' Zoo | Netherlands |
| Chiba Zoo | Japan |
| Denver Zoo | USA |
| Gaia Zoo, Kerkrade | Netherlands |
| Higashiyama Zoo | Japan |
| Howletts Wildlife Park | UK |
| Kyoto Zoo | Japan |
| La Vallée des Singes | France |
| LA Zoo | USA |
| Leipzig zoo | Germany |
| Lincoln Park Zoo | USA |
| Ouwehands Zoo | Netherlands |
| Pairi Daiza Zoo | Belgium |
| Philadelphia Zoo | USA |
| Saint-Martin-la-Plaine Zoo | France |
| San Diego Zoo | USA |
| Schmiding Zoo | Austria |
| Smithsonian's National Zoo | USA |
| Tierpark Hellabrunn | Germany |
| Ueno Zoo | Japan |
| Toronto Zoo | Canada |
| Wilhelma Zoo Stuttgart | Germany |
| Zoo d'Amneville | France |

# SI2: Glossary:

**Apex (of an AU)**: When the movement reaches the maximum intensity and no further increase in muscular action can be observed for that particular AU. The apex can be different each time the same AU is produced, both in how long it is maintained and regarding the maximum intensity.

**De-elongate**: The mouth (or another feature) appears to be shorter than usual in the horizontal plane.

**Dorsal plane**: Imaginary plane of reference that sections the trunk of an individual, parallel to the back and belly. It divides the body into dorsal (upper) and ventral (lower) segments.

**Elongate**: The mouth (or another feature) appears to be longer than usual in the horizontal plane. Opposite of de-elongate.

**External ear:** Portion of the ear external to the head, also designated as auricle, pinna, or concha.

**Facial morphology:** Series of complex facial traits, influenced by both genetics and environmental factors, unique to each individual, such as face and facial feature size, shape and location.

**Facial landmarks:** Key points or structures on the face which are common between individuals of a species. In FACS, they aid in identifying AUs.

**Facial nerve** (cranial nerve, CNVII): The seventh cranial nerve that supplies most of the muscles of the head, and in particular the ones involved in facial behaviour. The facial nerve leaves the lateral border of the corpus trapezoideum in the brain, near its cranial edge, between the fifth and eighth nerves, radiating to the face.

**False indicator**: When a cue used for identifying an AU is already present in the appearance on the neutral face, being thus dependent of the individual unique facial morphology.

**Flatten**: To decrease indentations or salient areas. For example, the lips may appear flattened against the teeth, i.e. they protrude less than usual.

**Glabella**: In humans, it is the space between the two eyebrows. In non-human animals, the glabella is located on the frontal region area, in the midline of the browridge.

**Holistic**: Same as gestalt or global. Refers to the whole that it is formed by several parts. In reference to facial expressions, it means encompassing the whole face/all facial features/all facial movements as one object (e.g. a sad facial expression is an holistic categorisation, while lip corners pulled downwards is one part of that facial expression). In reference to facial processing, it means the face is perceived globally as one single object, instead of by its features/parts.

**Homology (anatomical)**: Similarity between structures from two different species that diverged from a common ancestor. The function of these structures may or may not be identical.

**Mandible**: Lower jaw bone.

**Mastication**: Chewing.

**Mastication muscles**: muscles related to functions such as chewing, jaw movement, swallowing and vocalisations, that include the masseter, temporalis, pterygoid and digastric muscles. These muscles are not supplied by the facial nerve, except for the digastric muscle.

**Maxilla**: Upper jaw bone.

**Mental region**: Facial feature below the mouth, located in the same area as the human chin. The chin (mental protuberance in the skull bone) is a unique anatomical feature present only in humans, and thus in animals this area is referred to as the mental region.

**Mimetic muscles**: The muscles that are supplied by the facial nerve (cranial nerve, CNVII).

**Neutral** (in reference to an AU): Absence of any appearance change related to a particular AU, which can be observed before the onset and after the offset of an AU.

**Neutral face**: Absence of any appearance change in the face due to visible muscular contraction. It serves as a baseline comparison for coding the presence of AUs.

**Nostril wing or ala**: Portion of the nose around the nostril cavity on each side of the nose.

**Offset** (of an AU): Length of time from the end of the apex to the point where the muscle is no longer contracting (neutral).

**Onset** (of an AU): Length of time from the start of the movement till the apex.

**Pinna**: Projecting outer part of the ear that is visible.

**Protrude**: The lips (or other feature) move forward or out, away from the face, more than usual. Opposite of flatten.

**Rotation**: The movement of a part along its long axis. For example, the head rotates when looking left or right along its longer dimension (from top of the head till the neck).

**Stretch**: The lips (or other feature) are pulled and the skin stretched like a rubber band.

**Tighten**: The lips (or other feature) appear tight, the lips are not relaxed or loose. The muscle within the lips has contracted.

**Transverse plane:** Imaginary plane of reference that is perpendicular to the dorsal plane. It sections the body into cranial (front) and caudal (back) segments.

**SI3: Action Descriptors:**

**Action Descriptors (ADs)** identify and describe more broad muscular movements or movements from non-mimetic muscles. The muscles responsible for ADs are not innervated by the facial nerve (cranial nerve VII [1]), and so usually are not considered facial expression movements. However, these movements are often observed in combination with AUs, altering their visual appearance. Hence, section “A. Proposed muscular basis” is omitted below for all ADs, as either no movements are related to facial musculature or the musculature has not been clearly identified. In addition, section “D. Subtle differences between AUs” is included below where necessary, to help distinguishing between similar ADs or AUs. Other miscellaneous movements classified as ADs including head and eye direction, gross behaviours, and visibility status are also briefly described below.

**AD19 - Tongue Show**

**B. Appearance changes:**

1. The tongue is extended outwards and reaches at least the inner lower lip (e.g., **S76a-S77a and S76b-S77b videos**).
2. The jaw must be lowered and the lips are separated. Therefore, this AD is always coded together with AU25+AU26 or AU25+AU27.

**C.** **Minimum criteria**: The tip of the tongue reaches at least the lower lip.

Although rarely seen, the tongue of gorillas can extend far outside the mouth and can fold upwards and downwards, and touching the nose (e.g., **S78a and S78b videos**). Hence, AD190 - Tongue Downwards and AD191 - Tongue Upwards are used to code different tongue movements outside the mouth.

**AD190 - Tongue Downwards**

**B. Appearance changes:**

1. The tongue is extended beyond the lips and folded ventrally.
2. The ventral part of the tongue can be seen touching/licking the mental region.
3. The jaw must be lowered and the lips are separated. Therefore, this AD is always coded together with AU25+AU26 or AU25+AU27.

**C. Minimum criteria to code AD190:** The tongue is projected beyond the lips and folds downwards.

**D. Subtle differences between AUs/ADs:** This AD is distinguished from AD19 due to a clear ventral movement of the tongue (forming a curve with the tip of tongue pointing downwards), while in AD19 the tongue is only seen in a craniocaudal axis motion (no curvature is observed, tip of the tongue points forward). AD19 and AD190 are mutually exclusive (i.e., each time the tongue is projected beyond the lower lip, only one of these movements can be coded).

**AD191 - Tongue Curl**

**B. Appearance changes:**

1. The tongue is extended beyond the lips and is curled upwards (e.g., **S78a and S78b videos**). It may touch the nose.
2. The jaw must be lowered and the lips are separated. Therefore, this AD is always coded together with AU25+AU26 or AU25+AU27.

**C. Minimum criteria to code AD191:** The tongue is projected beyond the lips and folds or curls upwards.

**D. Subtle differences between AUs/ADs:** This AD is distinguished from AD190 by the direction of movement of the tongue: in AD190 the tongue is projected downwards, while in AD191 the tongue is projected out and upwards. If the tongue is not folded upwards or downwards, then AD19 is coded. AD19, AD190 and AD191 are mutually exclusive (i.e., each time the tongue is projected beyond the lower lip, only one of these movements can be coded).

**AD119 - Lick**

**B. Appearance changes:**

1. The individual licks an object, by extending the tongue at least till the lips and touching an object once or in a repeated motion (e.g., **S79a and S79b videos**).

**C. Minimum criteria to code AD29:** The tongue touches an object (e.g., body part, stick, food) once or repeatedly.

**D. Subtle differences between AUs/ADs:** It is mutually exclusive to AD19, AD190, and AD191, as in AD119 the tongue is touching an object/body part, while in AD19/AD190/AD191 the tongue is projected out of the mouth, but doesn't touch anything.

**AD29 - Jaw Thrust**

**B. Appearance changes:**

1. The jaw is pushed forwards.
2. The mental region becomes more prominent and may bulge slightly.
3. If the mouth is open, the lower teeth are seen moving cranially (e.g., **S80a and S80b videos**).

**C. Minimum criteria to code AD29:** The jaw is projected forward.

**D. Subtle differences between AUs/ADs:** There may be individual variation in the relative position of upper and lower jaws in the gorilla. The mandible may be naturally positioned further forwards or backwards in relation to the maxilla and so AD29 should only be coded when movement is observed. Therefore, this movement cannot be coded from still images.

**AD30 - Jaw Sideways**

**B. Appearance changes:**

1. The jaw is moved laterally to one side in relation to the midline (e.g., **S80a-S81a and S80b-S81b videos**).
2. If the mouth is open, the lower teeth are seen moving to one side.

**C. Minimum criteria to code AD30:** The jaw is moved to one side of the face.

**AD32 - Bite**

**B. Appearance changes:**

1. One of the lips is introduced in the mouth and held between the teeth, biting the lip.

**C. Minimum criteria to code AD32:** A lip is held between the teeth.

**D. Subtle differences between AUs/ADs:** AD32 may be confused with AU28, but in the latter the lip is not held by the teeth, and is instead rolled over the teeth or sucked inside. In certain movements it may be hard to distinguish these two Actions, so when in doubt if the lip is being bitten, code AU28.

**AD34 - Puff**

**B. Appearance changes:**

1. The mouth is closed and air is forced inside the mouth inflating the cheeks (e.g., **S82a and S82b videos**).

**C. Minimum criteria to code AD34:** The mouth is closed and air is inflating the cheeks.

**AD36 - Bulge**

**B. Appearance changes:**

1. The tongue is pushed against the inside of the lips or cheeks protruding (e.g., **S82a and S82b videos**).

**C. Minimum criteria to code AD36:** The tongue is pushed against the inside of the lips or cheeks protruding.

**AD37 - Lip Wipe**

**B. Appearance changes:**

1. The tongue is moved across the lip in a swiping movement (e.g., **S83a and S83b videos**).

**C. Minimum criteria to code AD37:** The tongue is moved laterally across the lip(s) in a swiping movement.

**D. Subtle differences between AUs/ADs:** This AU differs from AD19 as the tongue is not only being extended outside the mouth and then retracted back in, but there is a lateral motion across the lips (either from left to right or vice-versa. This movement can be done in just part of the lips, upper lip only or lower lip only.

**AD101 - Scalp Retraction**

**A.** **Proposed muscular basis:** In scalp retraction movements the occipitalis and posterior auricularis muscle actions work together as a unit to pull and flatten the ears, tufts and scalp towards the back of the neck. This muscle complex has previously been described in gorillas [2].

**B.** **Appearance changes:**

1. The skin of the frontal and occipital region is pulled caudally and then ventrally. In gorillas this movement also makes the crest move and the movements is more conspicuous in the crest (**S84a-S85a and S84b-S85b videos**).
2. The distance between the crest base and browridge increases, the skin may appear stretched on the frontal region, and the hair direction accompanies the direction of the movement.

**C.** **Minimum criteria**: The skin on the frontal region is pulled caudally, with simultaneous crest movement.

**D.** **Subtle differences between AUs:** Scalp Retraction (AD101), previously described in crested [3] and Japanese [4] macaques was also observed in gorillas. AD101 in gorillas is a much subtler movement than in other species, perhaps due to the crest position that does not allow to see the scalp skin sliding back.

**SI4: Head and Eye Action Descriptors:**

Head movements recorded in video are possible to code based on the movement itself, or due to changes in the relative position of the head and body, usually from a neutral position (or anatomical standard position, where head and eyes are facing forward, chin at a 90º angle).

**AD51 - Head Turn Left:** The head moves to the left along a vertical axis.

**AD52 - Head Turn Right:** The head moves to the right along a vertical axis.

**AD53 - Head Up:** The head moves upwards (e.g., **S86a** **and** **S86b** **videos**).

**AD54 - Head Down:** The head moves downwards.

**AD55 - Head Tilt Left:** The head rotates tilting to the left side (e.g., **S87a and S87b videos**).

**AD56 - Head Tilt Right:** The head rotates tilting to the right side (e.g., **S88a and S88b videos**).

**AD57 - Head Forward:** The head moves forward and away from the body. In a neutral position, the neck is not well-defined, but with AD57, the neck elongates forward and becomes visible.

**AD58 - Head Back:** The head moves back and towards the body.

In **humans**, eye movements are easy to detect as the coloured iris is surrounded by a large white sclera. In **gorillas**, eye movements can be more difficult to detect, since the eyes usually have a dark brown iris without any sclera visible in the neutral position. Gorillas' eyes have a black round pupil that reacts to light, although in most lighting conditions the pupil is hard to distinguish from the iris. A more or less portion of white sclera can become visible on the outer eye corner when the eyeball moves laterally (e.g., **Figure S1, S89a and S89 videos**) aiding the detection of some eye movements. How much sclera is visible during eye movements seem to present variation between individuals.

Head and eye positions tend to influence how each are perceived by humans [5], so it is important to look for cues other than position alone, such as sclera visibility or clear movement.

**AD61 - Eyes Turn Left:** The eyes move to the left. In large movements the sclera may show on the right side of the eye (e.g., **Figure S1**).

**AD62 - Eyes Turn Right:** The eyes move to the right. In large movements the sclera may show on the left side of the eye (e.g., **S89a and S89b videos**).

**AD63 - Eyes Up:** The eyes move upwards (e.g., **Figure S1**).

**AD64 - Eyes Down:** The eyes move downwards (e.g., **S89a and S89 videos)**.


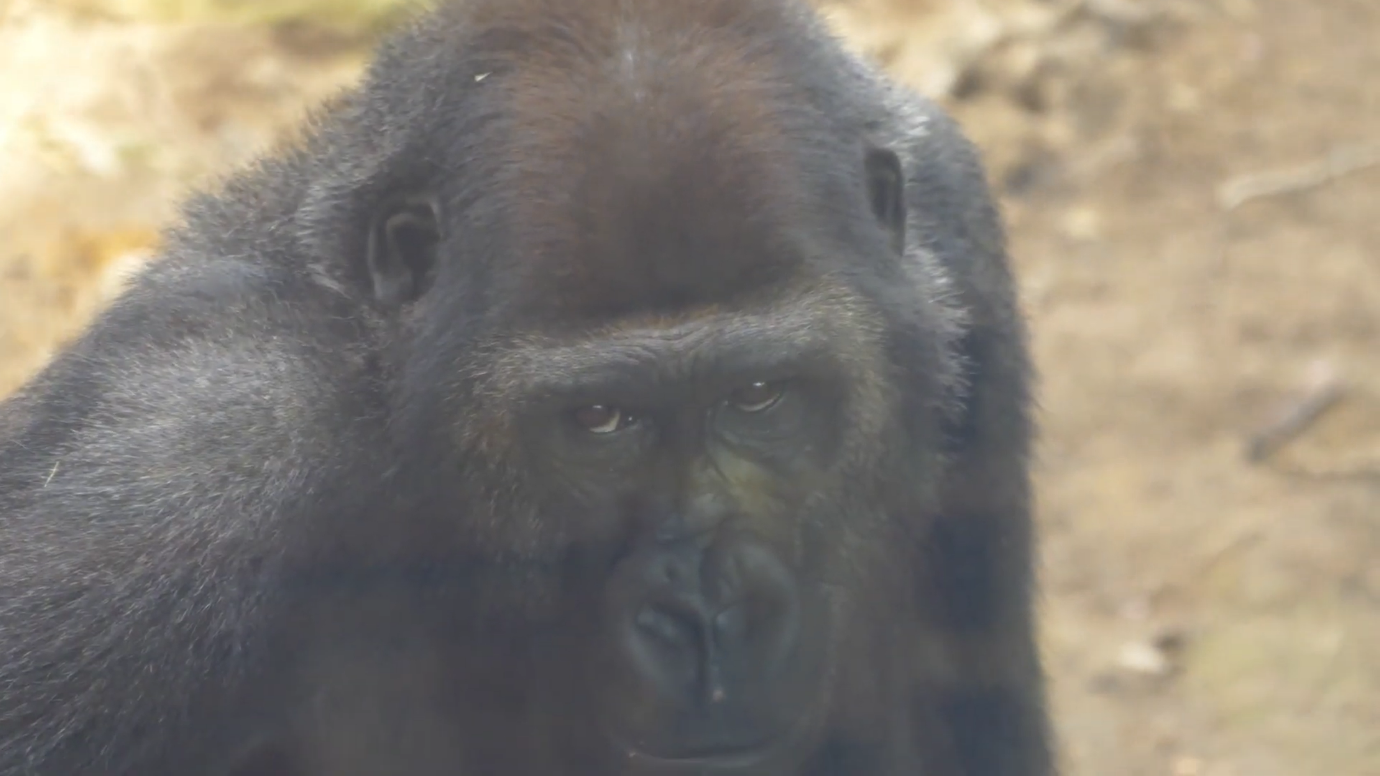

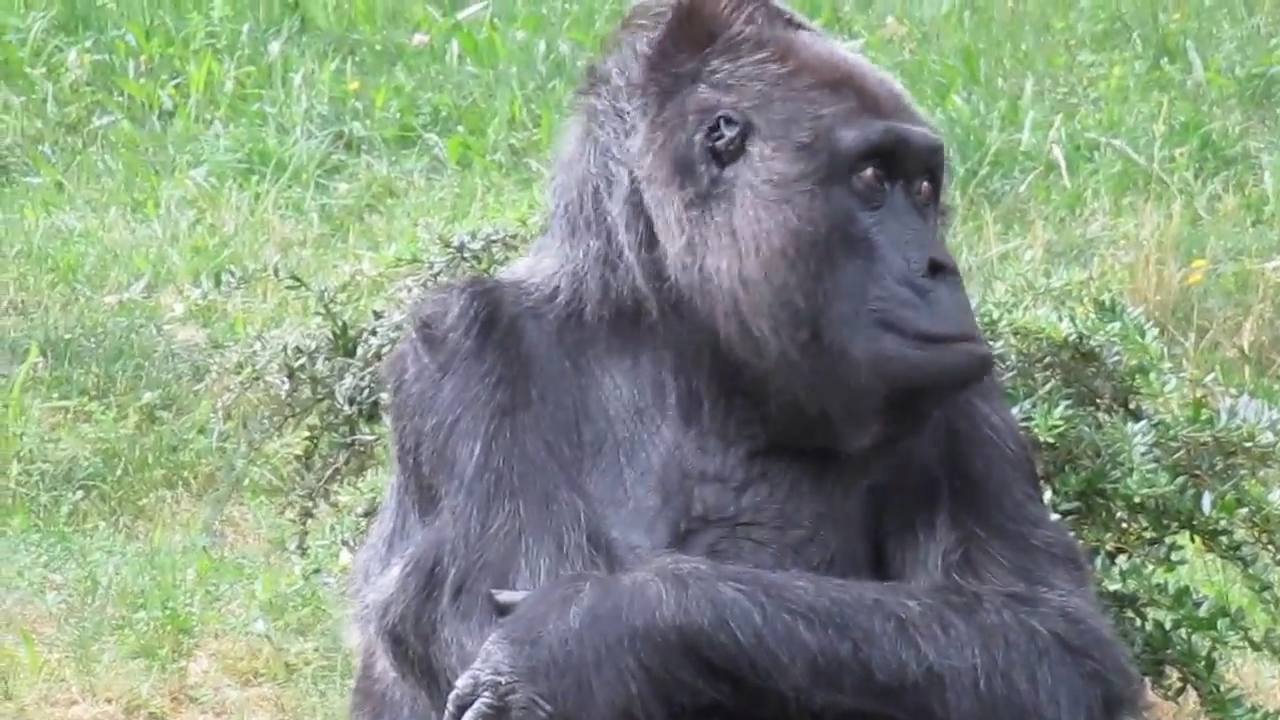


**Figure S1.** Left: AD63 - Eyes Up. Right: AD61 – Eyes Turn Left. Both movements expose sclera. Other AUs present.

**SI5: Gross behaviour codes:**

Gross behaviour codes are more general categories of behaviour that are not based on facial actions and may or may not involve the face. These codes are useful to score alongside AUs and other ADs as they can impact on or modify appearance changes.

**AD40 - Sniff:** This AD refers to the act of smelling, where global movement with varied intensity is observed around the nose and nostrils. It can be accompanied by nostril or nasal shield AUs, although these movements can also occur independently.

**AD50 - Vocalisations:** Gorillas display a range of vocalisations and most of them change the movement of the mouth and nose. This should be accounted for when coding facial behaviour.

**AD80 - Swallow:** Passing food, water or saliva from the mouth to the oesophagus through the action of a range of non-mimetic muscles. In gorillas, swallowing can produce movement on the fur on the anterior side of the neck and just below the neck.

**AD81 - Chewing:** Feeding-related behaviour when food is introduced into the mouth and the jaws are moved repeatedly to break down the food, involves several AUs and ADs described above and in the main text (e.g., AU25+AU26+AU30). However, AD81 should be coded instead of these mastication AUs and ADs whenever mouth movements are primarily due to feeding-related behaviours, as these movements are not considered related to communication or emotion.

**SI6: Visibility codes:**

**AD 70 - Frontal Region Not Visible:** When the browridge and frontal region cannot be clearly seen and coded.

**AD 71 - Eyes Not Visible:** When the eyes cannot be clearly seen and coded.

**AD 72 - Lower Face Not Visible:** When the lower face cannot be clearly seen and coded.

**AD 73 - Entire Face Not Visible:** When the entire face is out of view and cannot be clearly seen and coded.

**SI7: Ear movements not observed in gorillas:**

In **humans**, three external ear muscles (auricularis superior, posterior and anterior) are present, but movements are rarely or not observed in most individuals. When humans are able to move their ears voluntarily, these movements are usually of small amplitude and seem to be non-functional [6], with most ear muscle activity reflexive and non-observable [7]. In **gorillas**, ear muscles which are homologous to the human ones have been identified [8], but are described as hard to analyse and have not been identified in other recently published anatomical works [9]. Furthermore, ear movements have not been observed during this work. It is unclear if we did not see movement due to the position of the ears surrounded by hair or sometimes covered by hair, or if these movements are not present in gorillas.

**References**:

1. Reighard J. Scientific Books: Text-Book of Vertebrate Zoology. Science. 1900;11: 344–346. doi:10.1126/science.11.270.344

2. Huber E. Evolution of Facial Musculature and Cutaneous Field of Trigeminus. Part II. The Quarterly Review of Biology. 1930;5: 389–437. doi:10.1086/394364

3. Clark PR, Waller BM, Burrows AM, Julle‐Danière E, Agil M, Engelhardt A, et al. Morphological variants of silent bared-teeth displays have different social interaction outcomes in crested macaques (Macaca nigra). American Journal of Physical Anthropology. 2020;173: 411–422. doi:10.1002/ajpa.24129

4. Correia-Caeiro C, Holmes K, Miyabe-Nishiwaki T. Extending the MaqFACS to measure facial movement in Japanese macaques (Macaca fuscata) reveals a wide repertoire potential. PLOS ONE. 2021;16: e0245117. doi:10.1371/journal.pone.0245117

5. Otsuka Y, Clifford CWG. Influence of head orientation on perceived gaze direction and eye-region information. Journal of Vision. 2018;18: 15–15. doi:10.1167/18.12.15

6. Bérzin F, Fortinguerra CRH. EMG study of the anterior, superior and posterior auricular muscles in man. Annals of Anatomy - Anatomischer Anzeiger. 1993;175: 195–197. doi:10.1016/S0940-9602(11)80182-2

7. Hackley SA. Evidence for a vestigial pinna-orienting system in humans. Psychophysiology. 2015;52: 1263–1270. doi:10.1111/psyp.12501

8. Diogo R, Potau JM, Pastor JF, dePaz FJ, Ferrero EM, Bello G, et al. Photographic and Descriptive Musculoskeletal Atlas of Gorilla: With Notes on the Attachments, Variations, Innervation, Synonymy and Weight of the Muscles. Taylor & Francis; 2010.

9. Rotenstreich L, Marom A. “Untying the knot”: The primitive orofacial muscle architecture in the gorilla (Gorilla gorilla) as a key to the evolution of hominin facial movement. FASEB J. 2023;37: e23137. doi:10.1096/fj.202300927R
