# Supplementary material for: GorillaFACS: The Facial Action Coding System for the Gorilla spp
Source: PLoS One. 2025 Jan 28;20(1):e0308790. doi: 10.1371/journal.pone.0308790 (PMC11774405; doi:10.1371/journal.pone.0308790)
Supplement: S2 File — Captions for the Supporting Information Videos. https://doi.org/10.1371/journal.pone.0308790.s033 (DOCX) [file pone.0308790.s033.docx]

**Captions for the Supporting Information Videos**

**S1a and S1b Videos**. AU1+2 - Brow Raiser in normal speed and in slow motion, respectively. The AU1+2 can be seen immediately after the individual looks forward; AU1+2 becomes more intense then when they look up. The browridge returns to neutral in the last frames of the video. Other AUs present.

**S2a and S2b Videos**. AU1+2 - Brow Raiser in normal speed and in slow motion, respectively. The individual produces an AU1+2 in the first frames of the video. The skin of the browridge rolls upwards. The browridge then returns to neutral. Other AUs present.

**S3a and S3b Videos**. AU1+2 - Brow Raiser in normal speed and in slow motion, respectively, on the right side of the browridge (AU1+2R), indicated by the yellow circle. The right side of the browridge is pulled upwards, creating an angular shape. Other AUs present.

**S4a and S4b Videos**. AU1+2 - Brow Raiser relaxation in normal speed and in slow motion, respectively. The video starts with AU1+2 in action and then the brow returns to neutral. Other AUs present.

**S5a and S5b Videos**. AU1+2 - Brow Raiser in normal speed and in slow motion, respectively. Both sides of the browridge are puller upwards, and this movement is accompanied by frontal region hair going upwards as well. Other AUs present.

**S6a and S6b Videos**. AU1+2 - Brow Raiser in normal speed and in slow motion, respectively. The individual has an AU4 at the start of the video, then the browridge returns to neutral briefly, and then AU1+2 is produced. Other AUs present.

**S7a and S7b Videos**. AU4 - Brow Lowerer in normal speed and in slow motion, respectively. The individual produces an AU4 in the first few frames of the video, then the browridge returns to neutral briefly, and another AU4 is produced. Other AUs present.

**S8a and S8b Videos**. AU4 - Brow Lowerer in normal speed and in slow motion, respectively. The AU4 is more pronounced in the mid area of the browridge with the inner portions of the salient arches being pulled down and the skin of the browridge sliding down. The yellow arrow indicates the period in which AU4 is present. Other AUs present.

**S9a and S9b Videos**. AU4 - Brow Lowerer in normal speed and in slow motion, respectively. The AU4 can be seen in profile due to the salient browridge movement. AU138 - Nose Shield Expander also present. Other AUs present.

**S10a and S10b Videos**. AU1+2 - Brow Raiser and AU4 - Brow Lowerer in succession in normal speed and in slow motion, respectively. The individual produces an AU1+2 at around 2sec, then an AU4, and then another AU1+2 until the end of the video. Other AUs present.

**S11a and S11b Videos**. AU4 - Brow Lowerer and AU1+2 - Brow Raiser in succession in normal speed and in slow motion. The individual has an AU4 at the start of the video, and then produces an AU1+2R that alternates with return to a neutral brow. Other AUs present.

**S12a and S12b Videos**. AU6 - Cheek Raiser and AU7 - Lid Tightener in normal speed and in slow motion. The individual produces several AU6+AU7 during the video. Other AUs present.

**S13a and S13b Videos**. AU45 - Blink and AU43 - Eye Closure in normal speed and in slow motion, respectively. The individual produces an AU45 and then an AU43+AU6. Other AUs present.

**S14a and S14b Videos**. AU45 - Blink in normal speed and in slow motion, respectively. AD61 - Eyes Turn Left is also present. Individual presents elongation of both lips in a neutral face.

**S15 Video**. AU43 - Eye Closure in normal speed. AU43 is present at the start of the video, followed by AU45 - Blink.

**S16a and S16b Videos**. AU47 - Half-Blink in normal speed and in slow motion, respectively.

**S17a and S17b Videos**. AU9 - Nose Wrinkler in normal speed and in slow motion, respectively.

**S18a and S18b Videos**. AU9 - Nose Wrinkler in normal speed and in slow motion, respectively. Other AUs present.

**S19a and S19b Videos**. Unilateral AU9 - Nose Wrinkler in normal speed and in slow motion, respectively. The individual produces AU9R followed by AU9L. Other AUs present.

**S20a and S20b Videos**. AU10 - Lip Raiser in normal speed and in slow motion, respectively, produced by both individuals closer to the camera. The individual on the right produces an AU10 at around 5sec and the individual on the left produces an AU10 at around 7sec. Other AUs present.

**S21a and S21b Videos**. AU10 - Lip Raiser in normal speed and in slow motion, respectively. Other AUs present.

**S22a and S22b Videos**. AU10 - Lip Raiser in normal speed and in slow motion, respectively. Other AUs present.

**S23a and S23b Videos**. AU12 - Lip Corner Puller in normal speed and in slow motion, respectively. Other AUs present.

**S24a and S24b Videos**. Unilateral AU12 - Lip Corner Puller in normal speed and in slow motion, respectively. The individual produces an AU12R. Other AUs present.

**S25a and S25b Videos**. Unilateral AU12 - Lip Corner Puller in normal speed and in slow motion, respectively. The individual produces an AU12R. Other AUs present.

**S26a and S26b Videos**. Unilateral AU12 - Lip Corner Puller in normal speed and in slow motion, respectively. The individual produces an AU12L. Other AUs present.

**S27a and S27b Videos**. Example of false appearance change for AU12 - Lip Corner Puller in normal speed and in slow motion, respectively. The individual produces several AUs during mouth opening, including: AU16 - Lower Lip Depressor, AU25 - Lips Part, AU26 - Jaw Drop, AU18 - Lip Pucker, AU24 - Lip Presser; however, no AU12 is present.

**S28a and S28b Videos**. AU14 – Dimpler in normal speed and in slow motion, respectively. Lip corner is compressed and raises slightly almost at the end of the video. Red arrow indicates AU start and duration. Other AUs present.

**S29a and S29b Videos**. AU14 – Dimpler in normal speed and in slow motion, respectively. Other AUs present.

**S30a and S30b Videos**. AU14 – Dimpler in normal speed and in slow motion, respectively. Other AUs present.

**S31a and S31b Videos**. AU16 - Lower Lip Depressor in normal speed and in slow motion. Other AUs present.

**S32a and S32b Videos**. AU16 - Lower Lip Depressor in normal speed and in slow motion, respectively. Other AUs present.

**S33a and S33b Videos**. AU16 - Lower Lip Depressor in normal speed and in slow motion, respectively. Other AUs present.

**S34a and S34b Videos**. AU16 - Lower Lip Depressor in normal speed and in slow motion, respectively. Other AUs present.

**S35a and S35b Videos**. AU160 - Lower Lip Relax in normal speed and in slow motion, respectively. Other AUs present.

**S36a and S36b Videos**. AU160 - Lower Lip Relax in normal speed and in slow motion, respectively. Other AUs present.

**S37a and S37b Videos**. AU17 - Chin Raiser in normal speed and in slow motion, respectively. The individual produces several instances of AU17. Other AUs present.

**S38a and S38b Videos**. AU17 - Chin Raiser in normal speed and in slow motion, respectively. Other AUs present.

**S39a and S39b Videos**. Unilateral AU17 - Chin Raiser in normal speed and in slow motion, respectively. The individual produces an AU17R. Other AUs present.

**S40a and S40b Videos**. AU17 - Chin Raiser in normal speed and in slow motion, respectively. Other AUs present.

**S41a and S41b Videos**. AU17 - Chin Raiser in normal speed and in slow motion, respectively. Other AUs present.

**S42a and S42b Videos**. AU17 - Chin Raiser in normal speed and in slow motion, respectively. Other AUs present.

**S43a and S43b Videos**. AU18 - Lip Pucker in normal speed and in slow motion, respectively.

**S44a and S44b Videos**. AU18 - Lip Pucker in normal speed and in slow motion, respectively. Other AUs present.

**S45a and S45b Videos**. AU18 - Lip Pucker in normal speed and in slow motion. Other AUs present.

**S46a and S46b Videos**. AU18 - Lip Pucker in normal speed and in slow motion. Other AUs present.

**S47a and S47b Videos**. AU18 - Lip Pucker in normal speed and in slow motion, respectively. Other AUs present.

**S48a and S48b Videos**. AU22 - Lip Funneler in normal speed and in slow motion, respectively. Other AUs present.

**S49a and S49b Videos**. AU22 - Lip Funneler in normal speed and in slow motion, respectively.

**S50a and S50b Videos**. AU22 - Lip Funneler in normal speed and in slow motion, respectively. Other AUs present. Individual presents elongation of both lips in a neutral face.

**S51a and S51b Videos**. AU22 - Lip Funneler in normal speed and in slow motion. Other AUs present.

**S52a and S52b Videos**. AU22 - Lip Funneler in normal speed and in slow motion, respectively. Other AUs present.

**S53a and S53b Videos**. AU22 - Lip Funneler in normal speed and in slow motion. Other AUs present.

**S54a and S54b Videos**. AU22 - Lip Funneler in normal speed and in slow motion, respectively. Other AUs present.

**S55a and S55b Videos**. AU122 - Lower Lip Inner Curl in normal speed and in slow motion, respectively. Other AUs present.

**S56a and S56b Videos**. AU222 - Lower Lip Extension in normal speed and in slow motion, respectively. Other AUs present.

**S57a and S57b Videos**. AU222 - Lower Lip Extension in normal speed and in slow motion, respectively. Other AUs present.

**S58a and S58b Videos**. AU24 - Lip Presser in normal speed and in slow motion, respectively. Other AUs present.

**S59a and S59b Videos**. AU24 - Lip Presser in normal speed and in slow motion. Other AUs present.

**S60a and S60b Videos**. AU24 - Lip Presser in normal speed and in slow motion. AU18 - Lip Pucker also present. Other AUs present.

**S61a and S61b Videos**. AU24 - Lip Presser in normal speed and in slow motion, respectively. Other AUs present.

**S62a and S62b Videos**. AU24 - Lip Presser in normal speed and in slow motion, respectively. Other AUs present.

**S63a and S63b Videos**. AU25 - Lips Part in normal speed and in slow motion, respectively. The individual first produces AU25 alone and then produces AU25+AU26 - Jaw Drop.

**S64a and S64b Videos**. AU26 - Jaw Drop in normal speed and in slow motion, respectively. AU25 also present.

**S65a and S65b Videos**. AU26 – Jaw Drop without AU25 – Lips Part, in normal speed and in slow motion, respectively.

**S66a and S66b Videos**. AU27 - Mouth Stretch in normal speed and in slow motion, respectively. AU25 also present. Other AUs present.

**S67a and S67b Videos**. AU28 - Lips Suck in normal speed and in slow motion, respectively. Other AUs present.

**S68a and S68b Videos**. AU28 - Lips Suck in normal speed and in slow motion. The individual produces an AU28B (bottom lip only). Other AUs present.

**S69a and S69b Videos**. AU138 - Nose Shield Expander in normal speed and in slow motion, respectively.

**S70a and S70b Videos**. AU139 - Nose Shield Flattener in normal speed and in slow motion, respectively.

**S71a and S71b Videos**. AU139 - Nose Shield Flattener in normal speed. Other AUs present.

**S72a and S72b Videos**. AU238 - Nose Downwards in normal speed and in slow motion, respectively.

**S73a and S73b Videos**. AU38 - Nostril Dilator in normal speed and in slow motion, respectively.

**S74a and S74b Videos**. AU39 - Nostril Compressor in normal speed and in slow motion, respectively. The individual produces several instances of AU39. Other AUs present.

**S75a and S75b Videos**. Combination of AU38 - Nostril Dilator and AU138 - Nose Shield Expander in normal speed and in slow motion, respectively. Other AUs present.

**Video captions of ADs included in the SI:**

**S76a and S76b Videos**. AD19 - Tongue Show in normal speed and in slow motion, respectively. Other AUs present.

**S77a and S77b Videos**. AD19 - Tongue Show in normal speed and in slow motion, respectively. The individual also presents an AU43.

**S78a and S78b Videos**. AD191 - Tongue Curl in normal speed and in slow motion. The individual produces 2 instances of AD191 followed by AD19. Other AUs present.

**S79a S79b Videos**. AD119 - Lick in normal speed and in slow motion. Other AUs present.

**S80a and S80b Videos**. AD29 - Jaw Thrust and AD30 - Jaw Sideways in normal speed and in slow motion, respectively. The individual first produces AD30 immediately followed by AD29. Other AUs present.

**S81a and S81b Videos**. AD30 - Jaw Sideways in normal speed and in slow motion, respectively. The individual produces several instances of AD30. AU160 is also present, with the loose lower lip dangling due to the action of AD30. Other AUs present.

**S82a and S82b Videos**. AD34 - Puff and AD36 - Bulge in normal speed and in slow motion, respectively. AD36 is produced first in the video and is indicated by a red arrow. AD34 is produced right after AD36 and is indicated by a green arrow. Other AUs present.

**S83a and S83b Videos**. AD37 - Lip Wipe in normal speed and in slow motion, respectively. Other AUs present.

**S84a and S84b Videos**. AD101 - Scalp Retraction in normal speed and in slow motion, respectively. AU starts on the same frame that the individual's hand moves up. Arrow indicates start of AD101. The scalp is seen returning to neutral after the hand of the individual is lowered. Other AUs present.

**S85a and S85b Videos**. AD101 - Scalp Retraction in normal speed and in slow motion, respectively. First arrow indicates crest going back to neutral, and second arrow indicates a full Scalp Retraction and then return to neutral. Other AUs present.

**S86a and S86b Videos**. AD53 – Head Up in normal speed and in slow motion, respectively.

**S87a and S87b Videos**. AD55 – Head Tilt Left in normal speed and in slow motion, respectively.

**S88a and S88b Videos**. AD56 – Head Tilt Right in normal speed and in slow motion, respectively.

**S89a and S89b Videos**. Examples of several eye movements, including AD62 – Eyes Turn Right and AD64 – Eyes Down, in normal speed and in slow motion. Other AUs present.
